# Elderly rats fed with a high-fat high-sucrose diet developed sex-dependent metabolic syndrome regardless of long-term metformin and liraglutide treatment

**DOI:** 10.3389/fendo.2023.1181064

**Published:** 2023-10-20

**Authors:** Vedrana Ivić, Milorad Zjalić, Senka Blažetić, Matija Fenrich, Irena Labak, Rudolf Scitovski, Kálmán Ferenc Szűcs, Eszter Ducza, Tamás Tábi, Fruzsina Bagamery, Éva Szökő, Rosemary Vuković, Alen Rončević, Dario Mandić, Željko Debeljak, Monika Berecki, Marta Balog, Adrienn Seres-Bokor, Anita Sztojkov-Ivanov, Judit Hajagos-Tóth, Srećko Gajović, Alen Imširović, Marina Bakula, Solomiia Mahiiovych, Robert Gaspar, Sandor G. Vari, Marija Heffer

**Affiliations:** ^1^ Department of Medical Biology and Genetics, Faculty of Medicine Osijek, Josip Juraj Strossmayer University of Osijek, Osijek, Croatia; ^2^ Department of Biology, Josip Juraj Strossmayer University of Osijek, Osijek, Croatia; ^3^ School of Applied Mathematics and Computer Science, Josip Juraj Strossmayer University of Osijek, Osijek, Croatia; ^4^ Department of Pharmacology and Pharmacotherapy, Albert Szent-Györgyi Medical School, Interdisciplinary Excellence Centre, University of Szeged, Szeged, Hungary; ^5^ Department of Pharmacodynamics and Biopharmacy, Faculty of Pharmacy, University of Szeged, Szeged, Hungary; ^6^ Department of Pharmacodynamics, Faculty of Pharmacy, Semmelweis University, Budapest, Hungary; ^7^ Department of Neurosurgery, Osijek University Hospital, Osijek, Croatia; ^8^ Clinical Institute of Laboratory Diagnostics, Osijek University Hospital, Osijek, Croatia; ^9^ Department of Medical Chemistry, Biochemistry and Clinical Chemistry, Faculty of Medicine, Josip Juraj Strossmayer University of Osijek, Osijek, Croatia; ^10^ Department of Pharmacology, Faculty of Medicine, Josip Juraj Strossmayer University of Osijek, Osijek, Croatia; ^11^ Croatian Institute for Brain Research, and BIMIS - Biomedical Research Institute Šalata, University of Zagreb School of Medicine, Zagreb, Croatia; ^12^ Department of Clinical Pathology and Forensic Medicine, Osijek University Hospital, Osijek, Croatia; ^13^ Department of Therapy № 1 and Medical Diagnostics, Hematology and Transfusiology, Faculty of Postgraduate Education, Danylo Halytsky Lviv National Medical University, Lviv, Ukraine; ^14^ Cedars-Sinai Medical Center, International Research and Innovation in Medicine Program, Los Angeles, CA, United States

**Keywords:** high-fat high-sucrose diet, diabetes mellitus, metabolomics, insulin resistance, sex differences

## Abstract

**Aim/Introduction:**

The study aimed to determine the effectiveness of early antidiabetic therapy in reversing metabolic changes caused by high-fat and high-sucrose diet (HFHSD) in both sexes.

**Methods:**

Elderly Sprague–Dawley rats, 45 weeks old, were randomized into four groups: a control group fed on the standard diet (STD), one group fed the HFHSD, and two groups fed the HFHSD along with long-term treatment of either metformin (HFHSD+M) or liraglutide (HFHSD+L). Antidiabetic treatment started 5 weeks after the introduction of the diet and lasted 13 weeks until the animals were 64 weeks old.

**Results:**

Unexpectedly, HFHSD-fed animals did not gain weight but underwent significant metabolic changes. Both antidiabetic treatments produced sex-specific effects, but neither prevented the onset of prediabetes nor diabetes.

**Conclusion:**

Liraglutide vested benefits to liver and skeletal muscle tissue in males but induced signs of insulin resistance in females.

## Introduction

Obesity, characterized by the accumulation of excessive fat tissue, is a major contributor to early disability and mortality, and its prevalence is reaching pandemic levels. Besides serving as energy storage, fat tissue is an active endocrine organ. Moreover, it can trigger systemic low-grade inflammation by secreting inflammatory cytokines (1). A causative relationship between obesity-related inflammation and insulin resistance has been established (2). Affected individuals cope with progressive insulin resistance by ever-increasing insulin secretion, up to the point where this adaptive strategy becomes insufficient and type 2 diabetes mellitus (DM2) develops (3). DM2 and obesity are associated with higher risks for many life-threatening conditions, including cardiovascular disease and unfavorable outcomes in patients diagnosed with the novel coronavirus disease (COVID-19) (4, 5). Therefore, an effort to decelerate or stop the progression of obesity-triggered metabolic syndrome in its early stages is warranted.

Metformin, the gold standard in the treatment of DM2, is implicated in the slowed progression of insulin resistance to DM2 (6) but is also discussed as a potential senescence therapy in apparently healthy people (7). In addition, liraglutide (a glucagon-like peptide 1 analog with euglycemic and weight-reducing effects) has been approved for clinical use in obese diabetic individuals (8). Some studies suggest that weight reduction alone might be sufficient to prevent the progression of initial insulin resistance to full-blown DM2 (9). However, since liraglutide has been mostly studied in previously diagnosed diabetic and obese patients, little is known about its preventive potential.

As obesity and DM2 are mainly caused by chronic caloric surplus (2), rodent dietary models of high-fat diet, high-fat and high-fructose diet, or high-fat and high-sucrose diet (HFHSD) exhibit characteristics observed in human metabolic syndrome (10), and the latter (HFHSD) is the closest to the modern Western diet. Although these diets can induce (pre)diabetes in rodents, most of the studies are not prolonged enough to adequately reflect the chronic setting in which dietary effects normally take place in humans (11–15).

In humans, DM2 predominantly develops in elderly populations. Chronic low-grade systemic inflammation, underlying both aging and obesity, may be the culprit behind many age-related conditions, including insulin resistance (16). Despite this, most HFHSD rodent studies were conducted on young adult animals (14, 15, 17–20). Furthermore, females and males differ in body composition, adipose tissue metabolism, weight gain susceptibility, as well as cardiometabolic and dysglycemic risks (21–23). Yet, the available HFHSD rodent studies have included either male or female animals (13, 14, 17, 18). Finally, the evaluation of dietary animal models warrants whole-body analyses, since obesity and DM2 influence the brain as well as peripheral tissues (1). Still, most available studies focused solely on either the central or the peripheral phenomena (10, 13, 15, 18).

This study was conducted on male and female aged rats to address the possibility of sex-specific effects using whole-body analyses. It assessed the consequences of a long-term, obesity-inducing diet as well as the potential of early and long-term pharmacologic interventions to prevent the development and progression of DM2.

## Results

The experimental design included rats of both sexes (32 males and 32 females). When they were 45 weeks old (middle-aged), they underwent either a standard or HFHS diet (16 vs. 48 rats, experimental weeks 1–18). After 5 weeks of the HFHS diet, metformin or liraglutide medication was initiated, and it lasted a further 13 weeks (32 treated rats, experimental weeks 6–18). There were in total four experimental groups, each consisting of 16 rats (eight males and eight females): standard diet (STD), HFHS diet only (HFHSD), HFHSD and subsequent metformin medication (HFHSD+M), and HFHSD and subsequent liraglutide medication (HFHSD+L). At the end of the study, the animals were 64 weeks old (i.e., the onset of senescence) (24, 25). They ate a specific diet for 18 weeks (throughout the entire middle-age period), and those treated received medication for 13 weeks (**Extended Data Figure 1**). Senescence of females at the end of the study was proven by measuring estrogen values (6–12 pg/mL in all female rat groups).

### Lack of weight gain in liraglutide-treated animals on HFHSD was accompanied by increased caloric intake and a loss of visceral fat in females

**Figure 1:**
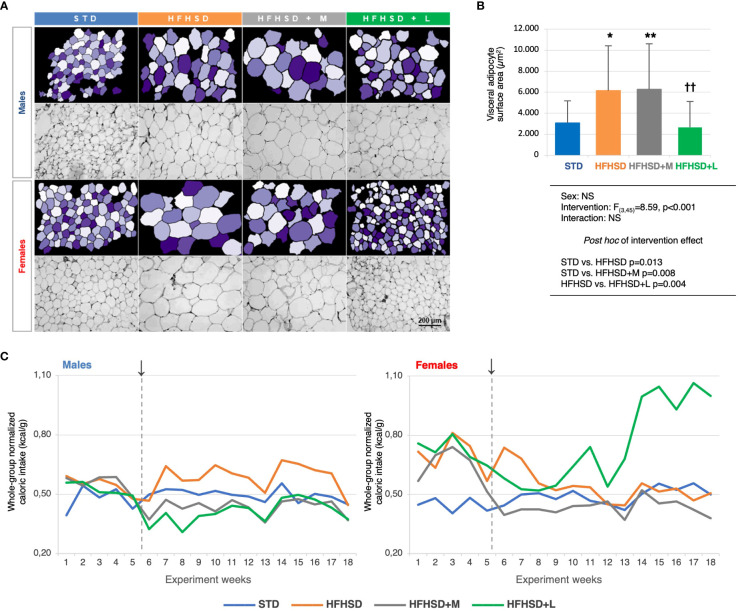
Liraglutide treatment in elderly rats on a high-fat high-sucrose diet reduced the surface areas of the visceral adipocytes, but triggered polyphagy in the female animals. **A)** Micrographs of the hematoxylin-eosin-stained visceral adipose tissue samples (magnification 200×) (bottom rows) and matching images obtained in CellProfiler (upper rows). **B)** Between-group comparison of average visceral adipocyte surface areas (μm2), two-way ANOVA and Games-Howell post hoc test for between-group comparisons: *compared to STD, †compared to HFHSD; *p < 0.05, **/††p < 0.01, NS – not significant. **C)** Whole-group caloric intake normalized for the whole-group body mass (kcal/g) per experimental week. ↓ – introduction of the antidiabetic drugs. Abbreviations: STD – standard diet group, HFHSD – high-fat high-sucrose diet group, HFHSD+M – HFHSD treated with metformin, HFHSD+L – HFHSD treated with liraglutide.

To explore the effects of the diet and medication (subsequently referred to as “intervention”) on the obesity-induced features, body mass was measured (**Extended Data Figure 2A**), and the visceral adipose tissue was characterized in detail (**Figures 1A, B**; **Extended Data Figure 2B**). Diet and treatment had no influence on body weight. Significantly larger visceral adipocyte surface area were detected in the HFHSD and HFHSD+M animals compared to those in the STD groups, while animals treated with liraglutide did not significantly differ from the STD animals.

To get insight into the overall metabolic change reflected in the polyphagia as a symptom of diabetes, a normalized approximation of the weekly caloric intake for each group was calculated as the ratio of the whole-group caloric intake and the whole-group body mass. By using kcal instead of g of food and animal mass instead of the number of animals, we nullified the difference between the two diets and the loss of animals during the study. Unexpectedly, the females treated with liraglutide experienced an abrupt increase in caloric intake after the experimental week 13, reaching almost twofold higher values relative to other experimental groups (**Figure 1C**). To quantify the observed changes, marginal means of caloric intakes were estimated for the period prior to the intervention (weeks 1–5), of the early intervention (weeks 6–10), and the long-term intervention (weeks 10–18). As expected, metformin significantly decreased the caloric intake in both sexes, while liraglutide did it only in males. In females, liraglutide paradoxically increased the caloric intake after long-term intervention, indicating development of metabolic inefficiency (**Extended Data Figure 2C**).

### Females tolerated acute hyperglycemic challenges less efficiently and exhibited decreased insulin sensitivity

Improved glucose tolerance and low glucose variability were expected to be the primary outcomes of antidiabetic treatment. Average values of areas under the curves (AUC) of glucose blood levels for each group during the glucose tolerance test (GTT) were calculated (**Extended Data Figure 3A**). In the experimental week 5, all HFHSD animal groups had significantly higher AUC values in comparison to the STD groups, revealing the decreased glucose tolerance. The same was observed in the experimental weeks 12 and 18 in the nonmedicated animals under HFHSD. In week 12, the AUC values of groups receiving medication approached STD group values, showing the acute benefits of antidiabetics. The males showed analogous results at experimental week 18; however, females of all groups (including the STD group) decreased glucose tolerance at this time point. With the onset of reproductive senescence, glucose tolerance worsened, particularly in the HFHSD female group, while the antidiabetic-receiving groups still benefited from the treatment. This result did not agree with the finding of polyphagia only in HFHSD+L females, especially because HFHSD females had by far the worst glucose tolerance of all the other groups.

**Figure 2:**
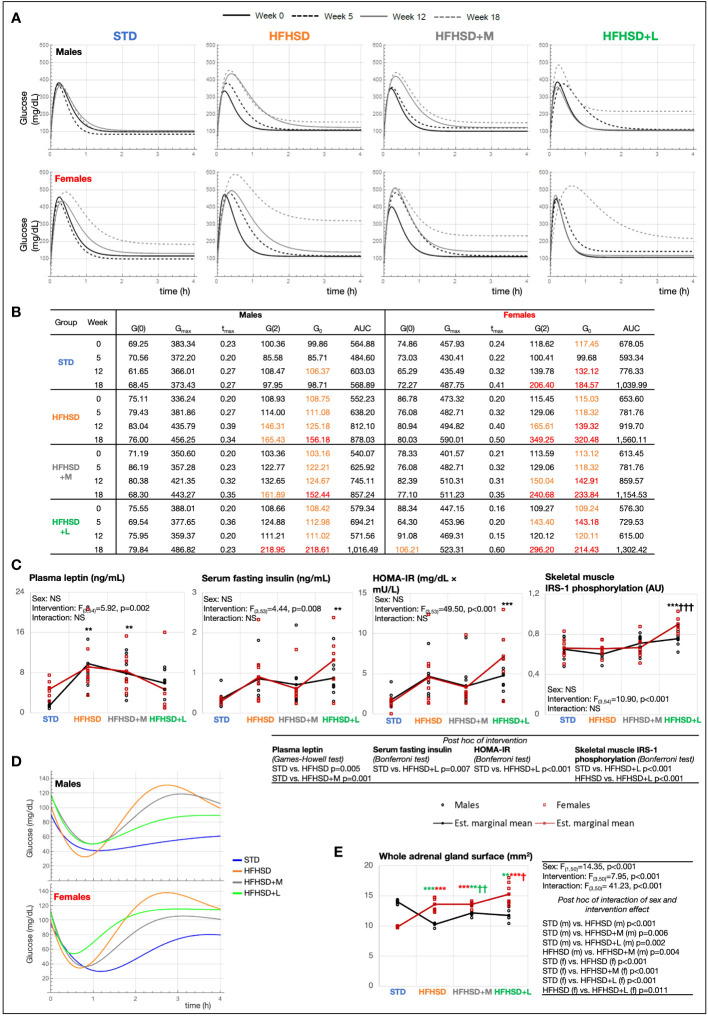
Aging in women impaired glucose metabolism more than in men, while long-term treatment with liraglutide exacerbated hyperinsulinemia and insulin resistance. **(A)** Model function [*G*(*t*)] of blood glucose concentration (mg/dL) based on measurements from glucose tolerance test (GTT) in experimental weeks 0, 5, 12, and 18. **(B)** Characteristics of model function: fasting blood glucose concentration [mg/dL) (*G*(0)], maximal glucose concentration (mg/dL) (*G*_max_), the moment at which *G*_max_ is reached (h) (*t*_max_), 2-h blood glucose at GTT [mg/dL) (G(2)], blood glucose setpoint (asymptote) (mg/dL) (*G*_0_), and area under the curve (AUC). Values in orange, prediabetes (100 mg/dL < *G*(0) < 125 mg/dL, 140 mg/dL < *G*(2) < 199 mg/dL); values in red, diabetes (*G*(0) ≥ 126 mg/dL, *G*(2) ≥ 200 mg/dL). **(C)** Interaction plots of intervention and sex effects on plasma leptin (ng/mL), serum fasting insulin (ng/mL), Homeostatic Model Assessment for Insulin Resistance (HOMA-IR) (mg/dL × mU/L), insulin receptor substrate 1 (IRS-1) phosphorylation in the skeletal muscle [arbitrary units (AU)]. **(D)** Model function [*H*(*t*)] of blood glucose (mg/dL) based on measurements from insulin tolerance test (ITT) in experimental week 18. **(E)** The adrenal gland surface. Two-way ANOVA; black symbol, experimental groups including both sexes; green symbol, male groups; and red symbol, female groups; */†*p* < 0.05, ^**/††^ *p* < 0.01, ^***/†††^ *p* < 0.001 (^*^compared to STD, ^†^compared to HFHSD). NS, not significant; f, female; m, male; STD, standard diet group; HFHSD, high-fat and high-sucrose diet group; HFHSD + M, HFHSD treated with metformin; HFHSD + L, HFHSD treated with liraglutide.

To get more detailed insight into sex-based differences in glucose tolerance, we used mathematical modeling of GTT data (**Figures 2A, B**). Derived parameters describing curves explained group progression in glucose variability (0-, 5-, 12-, and 18-week time points). The females belonging to all groups reached significantly higher glucose concentrations during the GTT (maximal glucose concentration (mg/dL) (*G*_max_)) compared to the males and were slower in reestablishing normoglycemia than their male counterparts. Blood glucose set points described by *G*_0_ followed by plasma glucose 2 h after load (*G*(2)), and fasting glucose (*G*(0)) were the best biomarkers of progressive metabolic failure. The *G*_0_ parameter describes the base value to which the function returns; that is why we assumed that this parameter can be physiologically best translated into the centrally given glucose set point. In our case, we calculated it based on the value of the entire group. **Figure 2A** shows that STD males at the beginning of the study reach the *G*_0_ value in just 1 h, while the females of the HFHSD+L group at the end of the study do not reach *G*_0_ even in 3 h, so the value of the function period (*T*) is also the highest in them. According to plasma glucose 2 h after load (*G*(2)), all examined groups, except STD males, developed prediabetes (HFHSD and HFHSD+M males) or diabetes (all the rest) according to official DM2 diagnostic criteria (140–199 mg/dL for prediabetes and ≥ 200 mg/dL for diabetes) (26, 27). HFHSD+L females also met the diagnostic criteria for the fasting glucose dysglycemia biomarker (100–125 mg/dL). Mathematical modeling revealed five additional parameters that were the lowest (coefficient of oscillation amplitude decline (α), and initial speed of blood glucose increase (*G*′(0)) or the highest (the basic period of function (*T*), maximal speed of glucose concentration decrease (*G*′_I_), and the moment at which *G*′_I_ is attained (*t*_I_)) in HFHSD+L females reflecting changes in the glycemia regulation (**Extended Data Figure 3B**). An additional proof of the credibility of the mathematical model is that the AUC values obtained by mathematical modeling correlated well with the AUC values obtained from real measurements.

To identify hormones underlying the observed GTT changes, leptin, insulin, corticosterone, and adiponectin were measured at the endpoint of the study (**Figure 2C**; **Extended Data Figure 3C**). As expected, the HFHSD and HFHSD+M groups had significantly higher leptin plasma levels relative to the STD group, whereas the plasma leptin levels in the HFHSD+L animals did not differ when compared to those in the STD and HFHSD groups. Observing the insulin serum levels, the HFHSD+L females had significantly higher fasting insulinemia compared to the STD females, but the same trend was not statistically significant in the male groups. The Homeostatic Model Assessment for Insulin Resistance (HOMA-IR) score was also calculated. The highest and statistically significant score was achieved by HFHSD+L females. Furthermore, phosphorylated tyrosine moieties of the insulin receptor substrate 1 (IRS-1) increased significantly in the skeletal muscle of all treated groups, but especially in HFHSD+L groups. Plasma corticosterone levels were not informative, while females in general exhibited higher adiponectin levels than males.

Improved insulin sensitivity was the expected secondary outcome of antidiabetic treatment; hence, the insulin tolerance test (ITT) was performed, and mathematically modeled ITT function was calculated in the experimental week 18 (**Figure 2D**; **Extended Data Figures 3D, E**) when we assumed insulin resistance could be developed. All the groups fed the HFHSD had significantly higher AUCs of glucose blood levels than the STD group. Mathematically modeled ITT functions (**Figure 2D**) revealed that the response to hyperinsulinemic challenge was highest in STD and lowest in HFHSD+L (minimal glucose concentration (*H*_min_)), indicating low insulin sensitivity under liraglutide treatment. STD group exhibited prolonged hypoglycemic levels lasting longer than 2 h. Animals fed the HFHSD had exaggerated glycemic compensatory responses in the ITT postacute recovery period, but both metformin-treated groups and males on liraglutide regained normoglycemia (*H*_0_). However, females in HFHSD+L that resisted acute hypoglycemia the best also remained in reactive hyperglycemia for the longest time, which can be explained by their highest tendency to develop insulin resistance relative to other animal groups.

Corticosterone, a potent insulin-antagonizing hormone, is commonly negatively associated with insulin sensitivity. Measuring its levels could provide a possible explanation for the dysglycemia observed in HFHSD+L females. Because a one-point measurement of corticosterone level is a low presentation for overall daily corticosterone fluctuations, we used Hans Selye’s historical finding of an association between adrenal gland size and cumulative corticosterone levels (28). The surface areas of mid-sections of adrenal glands were analyzed (**Figure 2E**). The male HFHSD animals and male groups receiving medication had significantly smaller adrenal glands than the STD animals, whereas exactly the opposite finding was present in females. The biggest adrenal gland surface in HFHSD+L females indicates the highest cumulative corticosterone levels in these animals, which may be related to metabolic disbalance and a shift in normoglycemia set point.

### Both antidiabetic treatments increased leptin sensitivity in hypothalamic satiety nuclei, but only liraglutide had a peripheral anti-inflammatory effect

**Figure 3:**
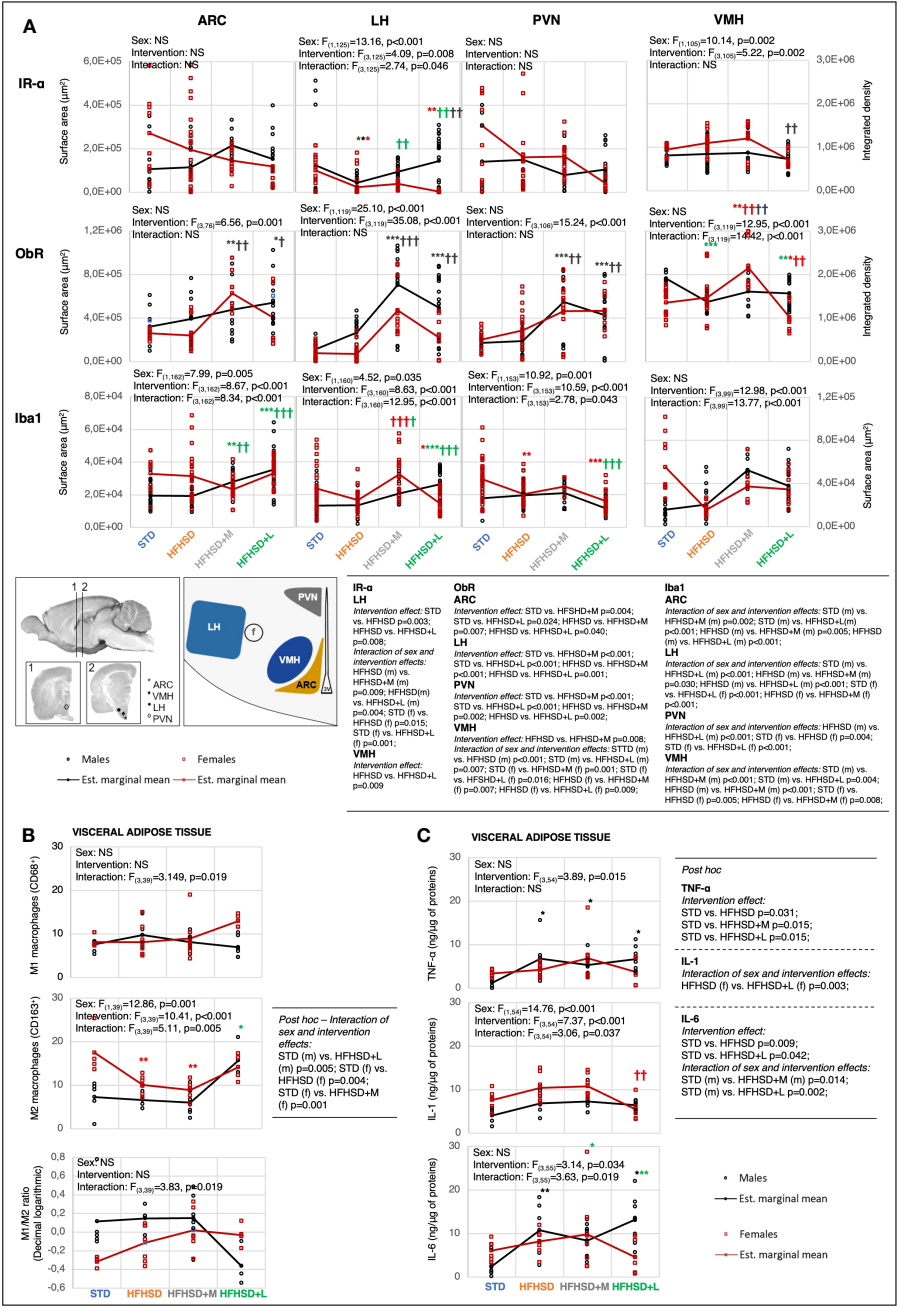
Metformin and liraglutide influenced high-fat high-sucrose diet-associated microinflammation in the hypothalamus and visceral adipose tissue in a sex-specific manner. **(A)** Interaction plots of intervention and sex effects on the expression level of the insulin receptor α-subunit (IR-α), leptin receptor (ObR), and ionized calcium-binding adapter molecule 1 (Iba1) in the following hypothalamic nuclei: arcuate nucleus (ARC), lateral nucleus of hypothalamus (LH), paraventricular nucleus (PVN), and ventromedial nucleus (VMH). The location of nuclei within the hypothalamus is indicated on the scheme below the interaction plots (f, fornix; 3V, third ventricle). Two-way ANOVA and Games–Howell *post-hoc* test for between-group comparisons. **(B)** Interaction plots of intervention and sex effects on the number of M1 macrophages (CD68) and M2 macrophages (CD163) in visceral adipose tissue per field of view at 100× magnification, and decimal logarithmic representation of M1 to M2 macrophage ratio in visceral adipose tissue. Two-way ANOVA and Games–Howell *post-hoc* test for between-group comparisons. **(C)** Interaction plots of intervention and sex effects on the TNF-α, IL-1, and IL-6 in the visceral adipose tissue. Two-way ANOVA and Games–Howell *post-hoc* test for between-group comparisons. Black symbol, experimental groups including both sexes; green symbol, male groups; red symbol, female groups. ^*/†^ *p* < 0.05, ^**/††^ *p* < 0.01, ^***/†††^ *p* < 0.001 (^*^compared to STD, ^†^compared to HFHSD). NS, not significant; f, female; m, male; STD, standard diet group; HFHSD, high-fat and high-sucrose diet group; HFHSD+M, HFHSD treated with metformin; HFHSD+L, HFHSD treated with liraglutide.

Improved insulin and leptin sensitivity in hypothalamic satiety nuclei, related to reduced food intake, was the expected tertiary outcome of antidiabetic treatment. Insulin serves as an acute satiety signal, leptin as a chronic one, and insulin-like growth factor (IGF) as a sub-acute signal that adjusts body temperature to energy resources. Their receptors initiate the signal responses in the brain and subsequently reduce feeding. The expression of insulin receptor α (IR-α), leptin receptor (ObR), and insulin-like growth factor 1 receptor β (IGF-1Rβ) was measured as markers of energy-sensing signaling pathways in the following brain nuclei: the arcuate (ARC), lateral hypothalamic (LH), paraventricular (PVN), and ventromedial hypothalamic (VMH) nuclei (**Figure 3A**; **Extended Data Figures 4.1A**, **4.2**, **4.3**, **4.4**). Selected hypothalamic nuclei are part of the neural network that controls feeding, and previous studies have shown that they are not equally prone to developing insulin/leptin resistance (29).

The high IR expression in the brain nuclei was associated with low serum fasting insulin levels in STD animals (in females in particular) and their potentially better central insulin response. Long-lasting HFHSD decreased the expression of IR in the LH nucleus, potentially due to increased serum insulin levels. The antidiabetic treatment reversed the receptor decrease in LH nuclei of males but not in females. The animal group with the lowest expression of IR relative to all other groups in all satiety nuclei (HFHSD+L females) also had the highest serum fasting insulin levels. This potential central insulin resistance indicated a small potential of insulin on the feeding switching function. This was also in agreement with the low whole-body sensitivity of insulin receptors (as measured by the ITT response) in HFHSD+L females.

HFHSD did not significantly affect ObR expression in individual nuclei, regardless of the increased plasma leptin levels. On the other hand, antidiabetic treatment was associated with a profound central effect: increased ObR expression was observed in all satiety nuclei in both males and females—more in the case of metformin, than liraglutide. It explained the major metformin beneficial effect: quick reaching satiety and no gain of weight despite an increase in adipocyte surface area. Contrary to HFHSD+M animals, the medication effect was lower in HFHSD+L groups, in particular females, probably due to cross-downregulation of ObR with increased insulin levels. HFHSD+L males, but not females, had increased expression of IGF-1R, which could provide them with additional relief from high-caloric HFHSD (**Extended Data Figures 4.1A**, **4.4**) by its ability to increase body temperature.

The fourth expected outcome of antidiabetic treatment was a reduction in low-grade inflammation. Neuro-inflammation was investigated in the same brain nuclei with the help of two markers: ionized calcium-binding adaptor molecule 1 (Iba1), a microglia marker (**Figure 3A**; **Extended Data Figure 4.5**), and the glial fibrillary acidic protein (GFAP), an astrocyte marker (**Extended Data Figures 4.1A**, **4.6**). Although some significant changes, mostly provoked by medication rather than HFHSD itself were shown, there was no clear correlation between hormonal changes and neuro-inflammatory status.

To investigate peripheral aspects of low-grade inflammation, proinflammatory (M1) and anti-inflammatory (M2) macrophages were analyzed in visceral adipose tissue using CD68 and CD163 markers, respectively (**Figure 3B**; **Extended Data Figure 4.7**). In addition to macrophages, the expression of the tumor necrosis factor α (TNF-α), interleukin 1 (IL-1), and interleukin 6 (IL-6) was analyzed in the visceral and subcutaneous adipose tissue samples (**Figure 3C**; **Extended Data Figure 4.1B**). Visceral adipose tissue was chosen for additional research because it appeared to be more related to inflammatory response.

Sex and intervention did not affect the M1 phenotype but did affect M2 in the adipose tissue. STD females had a significantly higher number of M2 macrophages compared to males (beneficial inflammatory response). However, when fed the HFHSD or treated with antidiabetics, both aspects of inflammatory responses were comparable between sexes. A large adipocyte size is a challenge for classical phagocytosis, whose inefficiency is reflected in the secretion of proinflammatory cytokines. In support of this, the TNF-α and IL-6 increase was interconnected with the downregulation of M2 lineages observed in groups with the highest adipocyte sizes, HFHSD and HFHSD+M. Liraglutide treatment reduced adipocyte size more in females than in males, and this resulted in consistently reduced secretion of inflammatory cytokines. A marked decrease in adipocyte size in liraglutide-treated females ultimately resulted in the proinflammatory response and highest M1/M2 ratio.

These results indicated that metformin was less able to alter the peripheral inflammatory response of animals exposed to HFHSD, whereas liraglutide had anti-inflammatory consequences only in males, but in females, liraglutide treatment led to an excessive reduction in adipocyte size and a reversal of the favorable treatment effect.

### Sex-specific metabolic difference in liver and skeletal muscle was enhanced by a high-fat and high-sucrose diet and antidiabetic drugs

**Figure 4:**
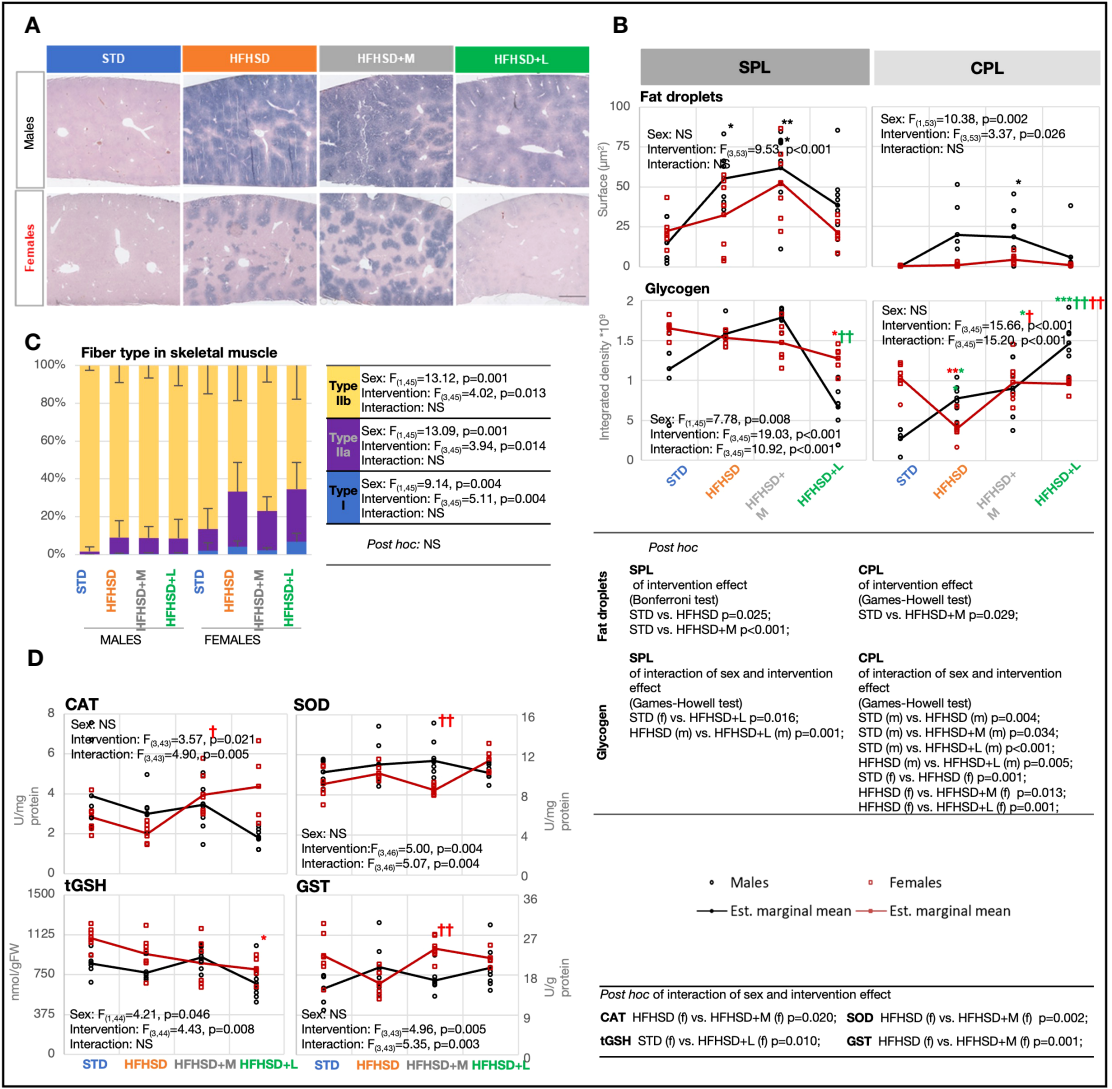
Male rats fed the high-fat and high-sucrose diet are more prone to excessive hepatic and skeletal muscle lipid accumulation. **(A)** Cross-section of liver stained using Sudan black, with annotated central (CPL) and subcapsular (SPL) parts. Scale, 500 μm. **(B)** Fat droplet measurement based on Oil red O staining and glycogen measurement based on metachromatic toluidin stain. Two-way ANOVA and Bonferroni or Games–Howell *post-hoc* test for between-group comparisons. **(C)** Percentage of fiber types I, IIa, and IIb in skeletal muscle based on staining with succinate dehydrogenase. Two-way ANOVA and Games–Howell *post-hoc* test for between-group comparisons. **(D)** Interaction plots of intervention and sex effects on the following parameters for determination of the oxidative-antioxidative status of skeletal muscle: catalase (CAT) (U/mg protein); superoxide dismutase (SOD) (U/mg protein); total glutathione (tGSH) (nmol/g of fresh tissue weight (FW)); glutathione *S*-transferase (GST) (U/g protein). Two-way ANOVA and Games–Howell *post-hoc* test for between-group comparisons; black symbol, experimental groups including both sexes; green symbol, male groups; red symbol, female groups; ^*/†^ *p* < 0.05, ^**/††^ *p* < 0.01, ^***/†††^ *p* < 0.001 (^*^compared to STD, ^†^compared to HFHSD). NS, not significant; f, female; m, male; STD, standard diet group; HFHSD, high-fat and high-sucrose diet group; HFHSD+M, HFHSD treated with metformin; HFHSD+L, HFHSD treated with liraglutide.

The fifth expected outcome of antidiabetic treatment was a slower progression of metabolic-dysfunction-associated fatty liver disease (MAFLD) caused by HFHSD. In normal-weight subjects, the presence of hepatic steatosis accompanied by at least two metabolic risk abnormalities is required for MAFLD diagnosis. With the exception of STD males, all animal groups fulfilled metabolic criteria for MAFLD and the presence of prediabetes or diabetes (**Figures 2B, C**). Nevertheless, pronounced liver steatosis was recorded just in the HFHSD and HFHSD+M groups (**Figure 4A**), as visualized by Sudan black staining. Due to their hydrophobicity, fat droplets were compact, and we used Oil Red staining to calculate their surface (**Figure 4B**; **Extended Data Figure 5.2**). The extent of lipid accumulation varied in subcapsular (SPL) and deeper parenchymal portions [central part (CPL)] of the liver (**Figures 4A, B**). Therefore, these regions were analyzed separately. In all groups, the subcapsular part accumulated more fat droplets, and steatosis was more pronounced in males than in females due to central part involvement. Male groups with the highest steatosis also had the highest levels of serum cholesterol and triglycerides (**Extended Data Figure 5.1A**), but without an increase of liver damage markers (**Extended Data Figure 5.1B**)—aspartate transaminase (AST) and alanine transaminase (ALT). Fat droplet accumulation in HFHSD+L groups was comparable to that in STD groups; that is, liraglutide successfully resolved hepatic steatosis. Also, HFHSD+L females significantly increased the liver mass to body mass ratio, probably due to both the loss of body mass and the loss of hepatic lipids **(Extended data Figure 5.1C**).

To determine whether lipid accumulation led to decreased glycogen storage, liver sections were stained with metachromatic toluidine (**Figure 4B**; **Extended Data Figure 5.3**). Surprisingly, the male groups with the highest steatosis, HFHSD and HFHSD+M, also had the highest glycogen content, both subcapsular and within the parenchyma. Contrary to that, liraglutide treatment depleted glycogen stores, especially in subcapsular hepatocytes. In conclusion, liraglutide treatment led to the depletion of glycogen stores.

The sixth expected outcome of antidiabetic treatment was a positive effect on HFHSD-induced skeletal muscle mitochondrial dynamics and antioxidant capacity. The content of mitochondria by skeletal muscle fiber types varies from high (type I), through intermediate (type IIa), to low (type IIb), while oxidative capacity correlates with the number of mitochondria in physiological conditions. Mitochondrial mass per fiber was determined by succinate dehydrogenase (Complex II) histological staining of skeletal muscles from the nuchal region (**Figure 4C**; **Extended Data Figure 5.4**). A large sex difference was already visible in animals on STD; females showed a higher percentage of red fibers (I and IIb) than males. Consumption of HFHSD led to a significant increase in mitochondrial mass in both sexes, but the ratio between red and white fibers (IIb) increased to a greater extent in females. Contrary to the diet, both antidiabetic treatments were unremarkable in the skeletal muscles of males. Nevertheless, metformin, known to affect mitochondrial efficiency by inhibiting Complex I, decreased the proportion of red fibers in females. Interestingly, metformin treatment in females had also the greatest effect on the antioxidant capacity of skeletal muscle (**Figure 4D**), leading to a significant increase in enzyme catalase (CAT) and glutathione *S*-transferase (GST). Medication with liraglutide did not affect mitochondrial mass and was associated with lower total glutathione (tGSH), but higher SOD in females.

The lipid droplet content of skeletal muscle was an indirect indicator of blunted inhibition of adipose tissue lipolysis in the development of insulin resistance, so we measured the average size of fat droplets using Oil Red staining (**Extended Data Figures 5.1D**, **5.5**). As expected, female groups had a higher average size of fat droplets than males, with the exception of metformin-treated males. Nevertheless, the accumulation of lipids was not accompanied by an increased risk for lipid peroxidation (**Extended Data Figure 5.1E**).

When these results are considered together, increased mitochondrial volume in HFHSD is a sign of serious changes in mitochondrial dynamic that are not matched with antioxidant capacity and pose a challenge to the quality control of mitochondria. The observed changes in skeletal muscle tissue deserved a more careful analysis.

### Diet and antidiabetic drugs have a significant effect on the metabolic profile of skeletal muscles in males but in less regard in females

Skeletal muscle tissue is an insulin-dependent organ, and its insulin resistance triggers diabetes (30). The expected outcome of antidiabetic treatment was a beneficial metabolic response opposing skeletal muscle insulin resistance. Therefore, the fresh-frozen samples of muscles from the nuchal region were subjected to the MALDI-TOF imaging mass spectrometry (IMS) that generated big data with the least loss of relevant molecules. Mass spectra were recorded in the range 300–700 Da (**Extended Data Figure 6.1**) in order to analyze metabolites and in the range 700–1,000 Da (**Extended Data Figure 6.2**) in order to analyze lipids.

**Figure 5:**
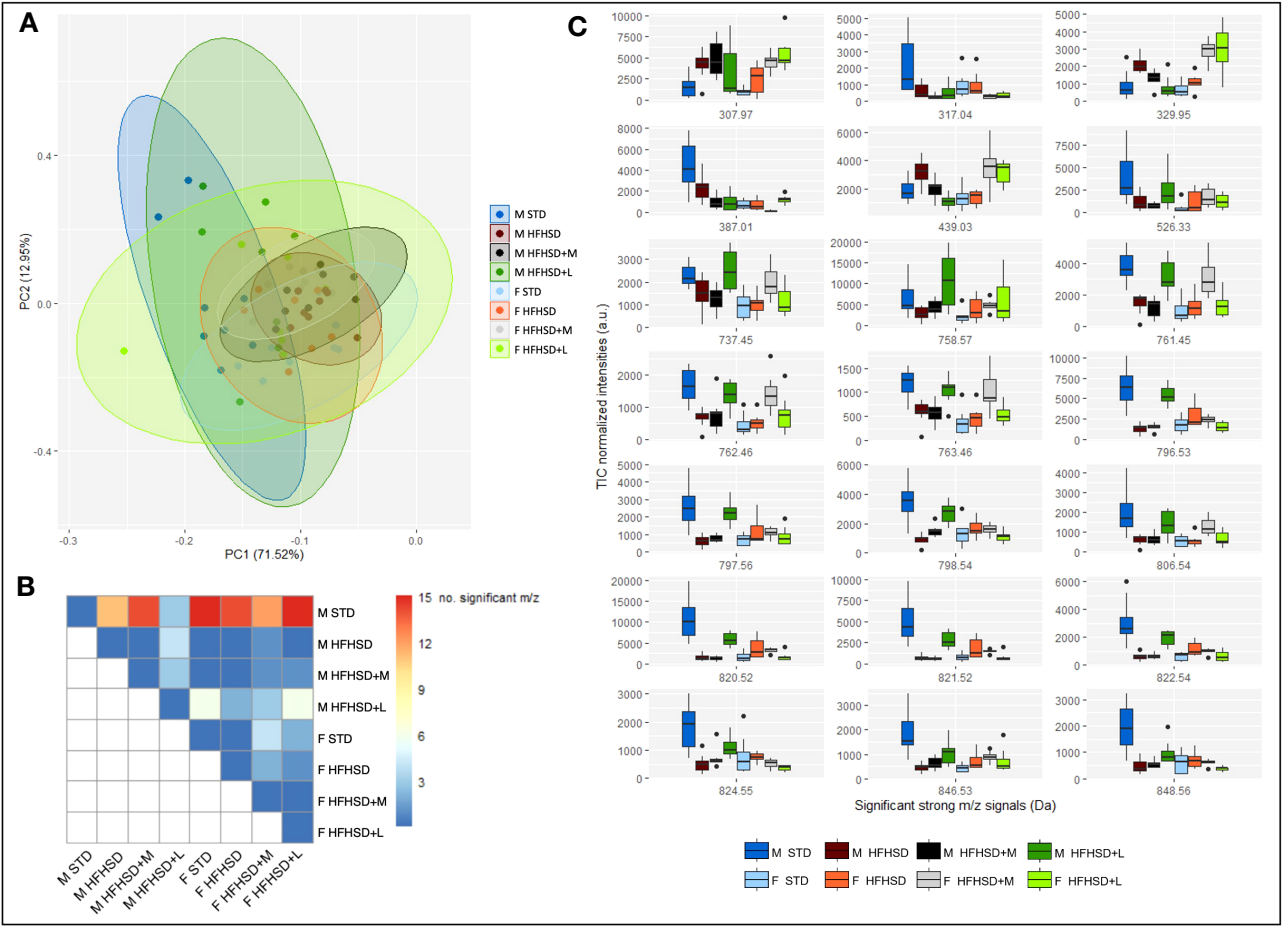
MALDI-TOF IMS analysis of Sprague–Dawley rats’ nuchal skeletal muscle shows sex-specific metabolic responses to HFHSD, liraglutide, and metformin treatments. **(A)** Principal component analysis (PCA) using strong m/z signals (signal intensity > 5% of the strongest signal) coming from the average total-ion-current (TIC)-normalized mass spectra recorded in the range 300–1,000 Da explains 84.47% variance. **(B)** Distribution of the significantly altered m/z signals by treatment pairs. **(C)** Box and whisker plots of significantly altered m/z signals. Each group sample size was eight (4 biological replicates × 2 technical replicates). F, female; M, male; STD, standard diet group; HFHSD, high-fat and high-sugar diet group; HFHSD+M, rats on HFHSD treated with metformin; HFHSD+L, rats on HFHSD treated with liraglutide; m/z, mass-to-charge ratio.

To identify patterns and trends or extract the most important features, principal component analysis (PCA) was used for big data visualization. Overlapping metabolic profiles were observed using the assumption that the sets of metabolic profiles may be represented by the chemical fingerprint containing strong signals (signal intensity > 5% of the strongest signal, *N* = 74) coming from the average TIC-normalized mass spectra in the range 300–1,000 Da (**Figure 5A**). However, out of 74 strongest m/z signals, 21 were significantly altered in at least one treatment pair selected by the omnibus false discovery rate (FDR) Kruskal–Wallis (KW) ANOVA followed by pairwise Dunn–Bonferroni test (**Figure 5B**). STD males were the most different (12–15 compounds) in relation to all other animal groups of both sexes. Nevertheless, liraglutide-treated males (HFHSD+L) were closest to STD males in muscle metabolic profile. In all-female groups, the muscle metabolic profile was similar (especially between the STD and HFHSD groups), and metformin treatment had a slightly larger effect than the liraglutide treatment. This result speaks in favor of pre-existing gender-specific differences in muscle metabolic profiles, and their different response to antidiabetic drugs.

To putatively identify significant m/z signals, METASPACE and HMDB databases were used (**Table 1**; **Extended Data Figure 6.3**). They included xanthurenic acid 8-*O*-sulfate, inosine monophosphate (IMP), phosphatidic acids (PA), and different types of phospholipids. Xanthurenic acide 8-*O*-sulfate (m/z 307. 97), considered to serve as a natriuretic hormone that enhances glycosuria, was lowest in STD groups (**Figure 5C**). IMP (m/z 387. 01), recently introduced as a beneficial nutriceutical affecting the energetic and antioxidant status of the liver and muscles (31) and previously connected with high physical activity in untrained animals, was highest in STD males. Levels of PA (m/z 737.45/761.45/763.46), precursors of phospholipids with positive effects on mitochondrial dynamics, were highest in STD males, liraglutide-treated males, and metformin-treated females.

**Table 1:** Statistically significant alterations in the strong m/z signal intensities of male and female rat nuchal skeletal muscle with tentative annotations.

m/z (Da)^a^	Adduct	Treatment pairs	Tentative endogenous metabolite annotation^b^	Metabolic and physiological role	Comments
**307.97**	M+Na	F STD/F HFHSD+L	Xanthurenic acid 8-*O*-sulfate	Trp/Kynurenine metabolism Natriuresis	*N*-Acetyl-l-glutamyl 5-phosphate is a result of database searches that is less likely to be present in muscles (1–3).
**317.04**	–	M STD/F HFHSD M STD/F HFHSD+M M STD/M HFHSD+M	–	–	Quinolinic acid is a result of the HMDB search but not of the METASPACE search. Database searches provide two more hits that are less likely to be present in muscles: methoxybrassenin B/wasalexin A, B, and homocarnosine.
**329.95**	–	F HFHSD+L/F HFHSD F HFHSD+M/F HFHSD F STD/F HFHSD+L M HFHSD+L/F HFHSD+L M HFHSD+M/F HFHSD+L M STD/F HFHSD+L F STD/F HFHSD+M M HFHSD+L/F HFHSD+M M STD/F HFHSD+M	–	–	3-Iodotyrosine is a result of HMDB and METASPACE search, but this compound is not likely to be present in muscles: instead, xanthurenic acid 8-*O*-sulfate adduct of type M+2Na-H is a more likely annotation.
**387.01**	M+K	M STD/F HFHSD M STD/F HFHSD+L M HFHSD/F HFHSD+M M STD/F HFHSD+M M STD/F STD M STD/M HFHSD+L M STD/M HFHSD+M	IMP	Impaired ATP biosynthesis during physical activity	– (4, 5)
**439.03**	–	F HFHSD+M/F HFHSD M HFHSD+L/F HFHSD+L F STD/F HFHSD+M M HFHSD+L/F HFHSD+M M HFHSD+L/M HFHSD	–		Glucocheirolin is a result of HDMB and METASPACE search, but this nutrient cannot be present due to controlled diets. Maleylacetoacetic/4-fumarylacetoacetic acids are results of HMDB but not of the METASPACE search. This m/z signal also corresponds to the matrix adduct 2CHCA+Na+K-H and may reflect the variable cellular K content.
**526.33**	–	M STD/F SD M STD/M HFHSD+M	–	–	LysoPC C20:4 adduct of type M-H_2_O+H is a result of HMDB search, but it is not likely to be produced in MALDI source.
**737.45/761.45/763.46^c^**	M+K	M HFHSD+L/F HFHSD M STD/F HFHSD M HFHSD+L/F HFHSD+L M STD/F HFHSD+L F STD/F HFHSD+M M HFHSD+M/F HFHSD+M M HFHSD+L/F SD M STD/F STD M SD/M HFHSD M HFHSD+M/M HFHSD+L M STD/M HFHSD+M	PA C36:3/PA C38:5/PA C38:4	Triglyceride/phospholipid biosynthesis PIP_2_/DAG signaling Insulin sensitivity	DG C38:4;O, DG C40:5;O, PG C31;O2, and PG C36:6;O2 are results of HMDB search, which are not present in the METASPACE database due to adduct types not likely to be produced in MALDI source (6).
**762.46**	–	M HFHSD+L/F HFHSD M HFHSD+L/F STD	–	–	PS C29:0 adduct of type M+H+HCOONa is a result of HMDB search only, but it is not likely to be produced in MALDI source. PG(PGJ2/i-12:0) adduct of type M+NH4 is also a result of HMDB search only, but it is not likely to be present in muscles. This m/z may correspond to PA C38:5 containing a (13)C atom.
**758.57; 796.53, 797.56, 798.54, 806.54, 820.52, 821.52, 822.54, 824.55, 846.53, 848.56^c^**	Different types	M STD/F HFHSD M HFHSD+L/F HFHSD+L M STD/F HFHSD+L M HFHSD+L/F HFHSD+M M STD/F HFHSD+M M HFHSD+L/F STD M STD/F STD M HFHSD+L/M HFHSD M STD/M HFHSD M HFHSD+M/M HFHSD+L M STD/M HFHSD+M	Different phospholipids	Insulin sensitivity	– (6, 7)

^a^FDR corrected pairwise Dunn–Bonferroni test applied on strong m/z signals (p < 0.05). ^b^METASPACE (8) (https://metaspace2020.eu) and HMDB (9) search using ±10 ppm acceptance limit. ^c^Some of the listed m/z signals are not significantly altered in all treatment pairs. F, female; HFHSD, high-fat and high-sucrose diet group; HFHSD+M, HFHSD treated with metformin; HFHSD+L, HFHSD treated with liraglutide; STD, standard diet group; M, male; m/z, mass-to-charge ratio. Each group sample size was eight (4 biological replicates × 2 technical replicates), and the m/z range was set to 300–1,000 Da.

In order to graphically represent major clusters, a heatmap was constructed using Euclidean distance, and Ward’s method was applied to the scaled significant m/z signals (**Extended Data Figure 6.4**). In concordance with PCA, all treatment groups were clustered together, which implied a large inter-individual variability. According to the samples’ dendrogram, liraglutide treatment achieved the expected effects in the muscles of most males (populations of STD and HFHSD+L males were grouped together). Metformin effects were shared between sexes (males and females on metformin were grouped together). The most dispersed classes were HFHSD and HFHSD+L females. m/z’s dendrogram showed clustering of phospholipids and their partial overlap with the PA. Conspicuously, IMP was between phospholipids and PA clusters. It was interesting to notice that m/z = 329.95 and 439.03 Da, which we were not able to uniquely identify, formed a cluster with the xanthurenic acid 8-*O*-sulfate.

Taken together, the skeletal muscle metabolic profile of STD males was different from all other groups, and the closest to it was HFHSD+L males. Also, HFHSD had a significant effect on males but not on females, which spoke in favor of developing muscle insulin resistance caused by menopause itself. Aging was a probable basis for large inter-individual differences (since biological and chronological age may mismatch) (32, 33), so it was not unexpected that the overall effect of antidiabetic drugs in female groups was negligible.

## Discussion

Glucotoxicity and lipotoxicity are the major two drivers of hyperinsulinemia and the concomitant development of multiorgan insulin resistance, culminating with the loss of β-cells as an ultimate deficit in DM2, independent of sex difference and aging (34). In this study, we developed the preclinical rat model to address the long-term effects of diet, aging, and sex in development of the DM2, which proved to be successful in recapitulating the whole sequence of its pathogenesis, from metabolic disorder to prediabetes and finally diabetes. As our motive was to increase the translational relevance, animals of both sexes were used, and the HFHSD was introduced in reproductive senescence (at 45 weeks). HFSHD indeed quickly leads to a metabolic disorder; 5 weeks after its introduction, the animals had early signs of prediabetes (i.e., elevated *G*_0_ parameter derived from the mathematical model), 7 weeks later, they reached clinical prediabetes, and in the next 6 weeks, clinical diabetes. The following mathematical parameters derived from the glucose tolerance test (GTT) were informative in monitoring the progression of metabolic disorder of the studied rats: *G*_max_, *G*_0_, *G*(2), and *G*(0). The length of the study (18 weeks) provided insight into the transition from prediabetes to diabetes. Subsequently, this allowed evaluation of the effects of the therapeutic interventions by metformin and liraglutide introduced at week 5. The long-term monitoring of the medications (for 13 weeks) distinguished early and late effects of therapy and revealed sex differences due to aging, diet, and antidiabetic treatment.

### Aging was the primary metabolic culprit in the DM2 pathology of females

Thanks to the fact that the study was initiated in middle-aged animals, it demonstrated the importance of aging as the primary metabolic culprit in DM2 pathology, which was more prominent in females. The females developed already on STD the indicators of metabolic disorder; an increase in parameters describing GTT-provoked glucose disposal [*G*_max_, *G*_0_, and *G*(2)], and higher skeletal muscle mitochondrial mass. Similarly, in our previous study (35) on young (3.5 months) and mature (12 months) rats, STD-fed perimenopausal females had a higher *G*_max_ than males. Therefore, an increase in *G*_max_ can be considered a prodromal sign of metabolic risk associated with aging, at least in females.

### The response to HFHSD was sex-specific implying that females may develop skeletal muscle insulin resistance, while males may develop hepatic insulin resistance

HFHSD exacerbates the female tendency toward glucose intolerance, dramatically increases skeletal muscle mitochondrial mass, and increases the associated potential development of insulin resistance. Glucose tolerance reflects the β-cell ability to offset insulin resistance by increased insulin secretion, and as long as this balance holds, glucose tolerance remains the same (36). At the end of the experiment, HFHSD females had glucose tolerance almost twice lower than males (elevated AUC results in GTT obtained with and without mathematical modeling). Relatively low AUC values in weeks 5 and 12 in SD and HFHSD indicate a prediabetes period. Our result speaks in favor of the hypothesis that prediabetes is a reversible condition, so we see a small difference between STD and HFHSD. Decompensation in glucose tolerance occurs somewhere between 12 and 18 weeks after prolonged feeding with HFHSD (20). Diminished glucose tolerance as a sign of β-cell loss was considerably higher in females than in males, and it supported earlier onset of diabetes in females compared to males. The increased number of mitochondria by itself posed a female-specific risk for the development of skeletal muscle insulin resistance due to increased reactive oxygen species (ROS) production (37, 38). Nevertheless, the increased mitochondria mitogenesis and their decreased efficacy represent potentially a female-specific protective response to HFHSD oriented toward the dissipation of excess energy.

On the other hand, HFHSD males developed liver steatosis accompanied by elevated levels of cholesterol and triglycerides in the blood, while the pathological changes of skeletal muscles included fat droplets and a significant decrease in IMP. The hepatic findings were consistent with research results for nonalcoholic fatty liver disease, recently renamed metabolic-associated fatty liver disease in order to reflect its association with metabolic syndrome morbidity, where men were more often affected with the disease than women (39–43). The skeletal muscle findings could be interpreted as a sign of reduced physical activity accompanied by a change in diet (44) and diminished supplies for ATP biosynthesis (31).

### Potential HFHSD-induced leptin and insulin resistance of satiety centers contributed to multiorgan disorder in prediabetes

**Figure 6:**
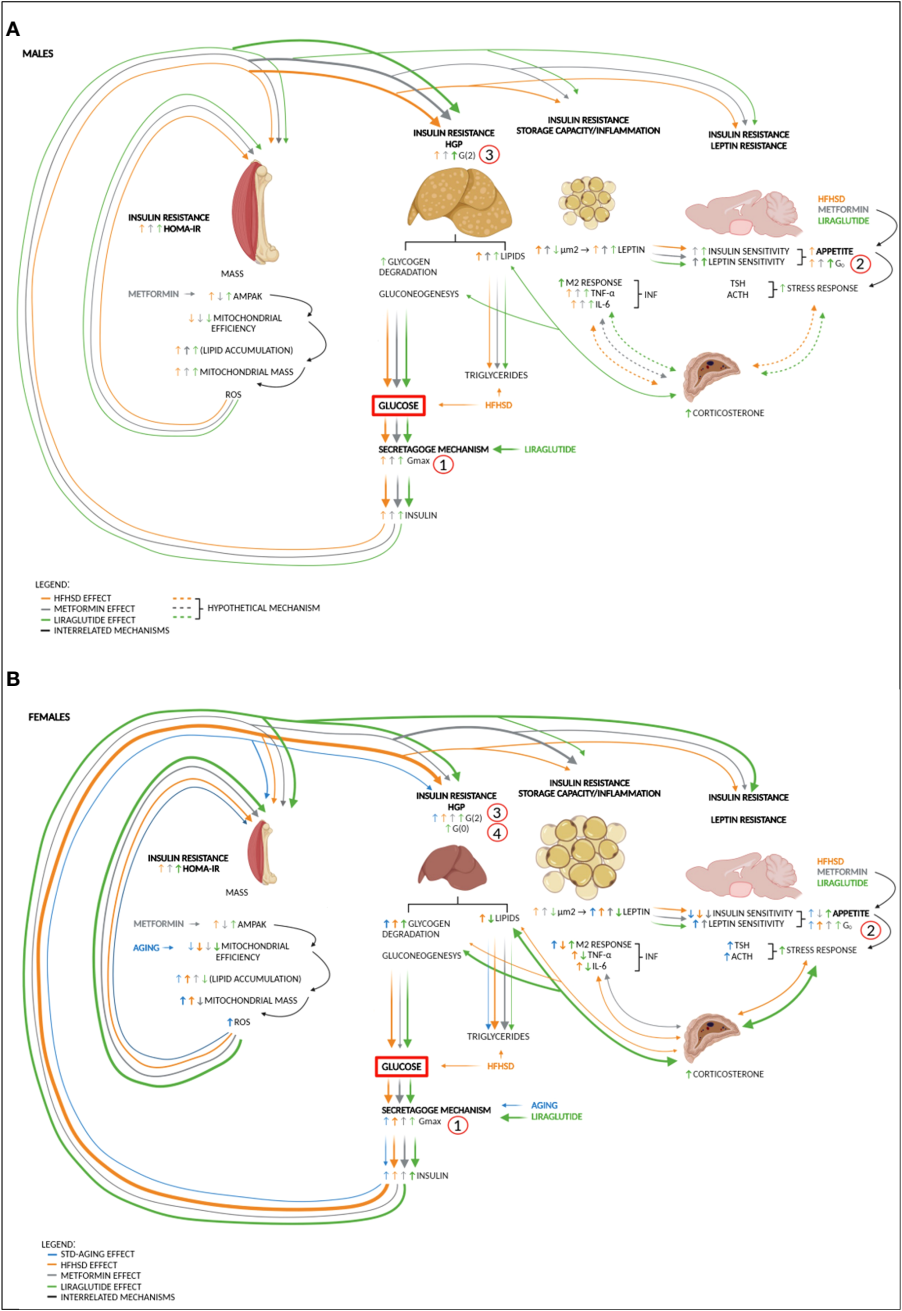
Sex-specific impairments in different organs contribute to DM2 to varying degrees—proposed mechanism. **(A)** High-fat and high-sucrose diet (HFHSD) is the major trigger of metabolic changes in males. Diet-induced hyperglycemia and hyperlipidemia burden the secretagogue mechanism, which leads to an increase in glucose variability (*G*_max_) and hyperinsulinemia. Hyperglycaemia and hyperinsulinemia increase hunger drive and gradually lead to insulin resistance of skeletal muscle (HOMA index), liver (hepatic glucose production (HGP), *G*(2)), and adipose tissue (fat storage capacity and the appearance of inflammation: M2 macrophages, TNF-α, and IL-6). In males, the liver is more sensitive to hyperinsulinemia than skeletal muscle, so metabolic-dysfunction-associated fatty liver disease (MAFLD) caused by HFHSD occurs more quickly. Under the action of antidiabetic drugs metformin and liraglutide, the sensitivity of the hypothalamic nuclei to insulin and leptin increases. Although both antidiabetics are anorexigenic, there is a gradual increase in the glucose set point (*G*_0_) under the influence of HFHSD. Potentially, further degradation of the satiety mechanism and low-grade inflammation in adipose tissue can lead to an increase in the stress response and entry into a vicious circle in which the stress response increases HGP (reflected in the increase of *G*(2) mathematical parameter) and contributes to insulin resistance of other organs. **(B)** In females, aging and especially HFHSD increase glucose variability. HFHSD-induced hyperinsulinemia affects skeletal muscle more than in males, which is seen as increased mitogenesis, reduced mitochondrial efficiency, and a less-efficient ROS response, which by itself increases muscle insulin resistance. The introduction of antidiabetic drugs only increases sensitivity to leptin but not to insulin. In liraglutide-treated females, plasma leptin and insulin sensitivity in the satiety centers are so low that both no longer contribute to extinguishing the stress response. The increased stress response further increases HGP, and in females treated with liraglutide, this leads to the full picture of DM2 (increased *G*(0) (fasting glucose) and hyperphagia). Metformin, as an insulin sensitizer, stops the activation of the stress response and reduces the mass of mitochondria in skeletal muscle, thereby delaying the onset of DM2. AMPAK, AMP-activated protein kinase pathway; DM2, diabetes type 2; *G*_0_, blood glucose set point; *G*(0), fasting glucose; *G*(2), plasma glucose 2 h after load; *G*_max_, maximal glucose concentration after glucose load; HFHSD, high-fat and high-sucrose diet; HGP, hepatic glucose production; HOMA-IR, Homeostatic Model Assessment for Insulin Resistance; IL-6, interleukin 6; M2, macrophage lineage; TNF-α, tumor necrosis factor-alpha. Numbers (1, 2, 3, and 4) indicate the sequence of events in DM2 development. Note that the thickness of the line indicates the strength of the effect. Dashed lines represent hypothetical mechanisms.

The disturbance of the satiety nuclei in the brain (reflected in the *G*_0_ parameter) was obligatory in the HFHSD-induced pathophysiology of DM2 in both sexes. Moreover, this aspect of diabetes development was documented through increased caloric intake. Satiety centers are influenced by a variety of nutritional factors (glucose, fatty acids, amino acids) and different hormones—insulin and leptin being the most important. In fact, the opposing action of insulin and leptin can explain why animals with HFHSD did not develop polyphagia despite the potential development of central insulin resistance (45). However, the satiety nuclei are not only involved in feeding control but also in various autonomous reactions, such as sympathetic activity, cardiovascular output, stress response, etc. What is important for this study is that the lateral hypothalamic satiety nucleus (LH) inhibits the activity of the paraventricular (PVN) (46) stress response-mediating nucleus and thus exerts insulin-mediated negative feedback regulation of the stress response. Judging by the size of the adrenal gland, which was significantly larger in females than in males on the HFHSD, the central stress homeostasis regulation was more efficient in males than in females. Nevertheless, the development of insulin and leptin resistance in the satiety centers explained how malfunction of the hypothalamic neuronal network contributed to a multiorgan disorder (**Figure 6**). Clinically relevant, glucose set point (as a functional output of satiety nuclei) which was reflected in mathematical parameter G_0_, proved to be reversible in STD groups, while caloric intake returned to the starting level over time in HFHSD groups.

### HFHSD increased the production of proinflammatory metabolites of the kynurenine pathway

Another sign of the onset of diabetes in both sexes due to HFHSD was increased production of xanthurenic acid 8-*O*-sulfate and quinolinic acid (putatively identified) in skeletal muscle tissue. These metabolites were generated in the kynurenine pathway, a major pathway for tryptophan metabolism, which is activated in diabetes and shown to contribute to inflammation, oxidative stress, and beta-cell dysfunction (47).

### Can prediabetic therapy slow the development of diabetes despite a diabetogenic diet?

The sex-specific effects induced by HFHSD included diminished glucose tolerance and a higher stress response in females, and they may have several important translational connotations. If aging is the main cause of metabolic deterioration, accelerated by HFHSD, then a dietary lifestyle intervention would be necessary but may not be sufficient for metabolic correction. From the example of STD females, an early intervention is desirable and should aim to correct glucose variability. Metformin and liraglutide are obvious choices for medication due to their anti-senescence or anti-obesogenic effects, respectively.

### Metformin had more benefits for females than for males due to the systemic disturbance of lipid metabolism

The two antidiabetic drugs had different sex-specific effects when tested in this long-term study. Metformin proved to be effective in the treatment of diabetes in both sexes. The beneficial effect was observed after the first week of treatment, in which animals reduced their food intake. The reduced food intake remained visible until the end of the study, and subsequent analysis of the hypothalamic satiety nuclei showed in three out of the four observed nuclei an increased level of leptin receptors (what could be interpreted as increased sensitivity to leptin). Changes in ObR expression are associated with changes in feeding behavior (48, 49). Therefore, a more precise assessment of sensitivity to leptin could be obtained by a functional study, i.e., administration of recombinant leptin and subsequent assessment of the amount of food consumed, as well as immunochemical determination of downstream molecules in the ObR signaling pathway (pSTAT3 and cFos) of the satiety nuclei (50).

Although metformin reduced food intake, this was not reflected in the morphology of visceral adipose tissue or plasma leptin levels. Just the opposite, HFHSD+M males had increased the surface area of adipocytes and fat droplets in adipocytes, liver, and skeletal muscle when compared to HFHSD males. Subsequently, the inflammation in adipose tissue increased, and it promoted systemic insulin resistance, which reduced the overall beneficial effects of metformin. As already described, enlarged adipocytes become dysfunctional in diabetes and secrete less protective and more inflammatory adipocytokines. When their fat storage capacity is exceeded, fats are stored in other tissues such as the liver, skeletal muscle, and pancreas, and they contribute to their insulin resistance or insulin secretion, as shown in this study as well (51). Metformin is known as an insulin-sensitizing medication, whose effect is achieved by activation of AMP-activated protein kinase (AMPK) and further inactivation of acetyl–CoA carboxylase (Acc1 and Acc2) by phosphorylation (52). The positive effects of metformin treatment come from the very blocking of lipolysis and lowering of the amount of free fatty acids whose lipotoxicity promotes insulin resistance of the liver and skeletal muscle (53).

Females responded better to metformin treatment than males. The beneficial effects, contrary to males, have been observed as improved glucose tolerance: parameters relevant for diabetes (*G*_max_, *G*_0_, and *G*(2)) decreased in comparison to the HFHSD-untreated females. Although serum fasting insulin and HOMA-IR, as signs of peripheral insulin resistance, did not decrease significantly, mitochondrial mass in skeletal muscle decreased. The reduction in mitochondrial mass corresponded with the finding of elevated levels of PA in the metabolic profile of the skeletal muscle of HFHSD+M females and its positive effect on mitochondrial fusion (54). Moreover, metformin in females was associated with decreased expression of superoxide dismutase (SOD), a central redox enzyme, which was also a bifurcation point between two signaling pathways that were involved in matching the efficacy of mitochondria with metabolic energy needs. Diminished levels of superoxide dismutase (SOD) enable the targeted propagation of superoxide signaling toward aconitase, an enzyme from the citric acid cycle serving as a metabolic switch in mitochondrial uncoupling and safe deciphering of energy (55–57). These two, elevated PA and downregulated SOD, can be related to the eventual counteracting of HFHSD-induced H_2_O_2_ production and lipid peroxidation.

In conclusion, the group of males treated with metformin maintained their prediabetic status, and the group of females maintained their diabetic status. The numerous beneficial effects justified the use of metformin as a prediabetic drug, especially in females. It would certainly be worth checking its effectiveness in combination with dietary measures and physical activity in future animal studies.

### The short-term positive effects of liraglutide are lost in long-term treatment due to hyperinsulinemia

The liraglutide treatment in both sexes was associated with initially reduced food intake, a significant reduction in the surface area of visceral adipocytes, and lower leptin levels. In addition, liraglutide also showed remarkable effects in reversing fatty liver changes and reducing peripheral inflammation. These findings were in accordance with the observed weight-reducing effects of liraglutide, where a significant overall decrease in the percentage of adipose tissue was frequently reported (8, 58). Without any doubt, these effects of liraglutide contributed to the almost complete normalization of glucose tolerance in both sexes after 7 weeks of treatment. Unexpectedly, the effect disappeared during the following 7 weeks of treatment: males outperformed their control HFHSD group and increased *G*_0_ and *G*(2) (which correspond to blood glucose set points and plasma glucose 2 h after load) up to diabetic values while females developed full clinical picture of DM2 (elevated *G*(0), fasting glucose).

We tried to identify what led to this unexpected deterioration in both sexes. Liraglutide treatment in males increased insulin and leptin sensitivity in satiety nuclei. Still, the *G*_0_ in this group surpassed that of other male groups. Conspicuously, the glycogen storage in the subcapsular part of the liver was depleted together with the fat droplets, which we interpreted as a sign of hepatic insulin resistance despite its recovery from steatosis. This was supported by the fact that serum fasting insulin and HOMA-IR were at the same level as in the HFHSD group, probably due to the major liraglutide effect of hyperinsulinemia. Despite the mentioned negative effects, IMS analysis of the skeletal muscle showed elevated levels of PA and various phospholipids, which contributed to regaining characteristics of STD males. Namely, in addition to the positive effect of PA on mitochondrial dynamics, phospholipid levels were previously positively associated with mitochondrial efficiency and skeletal muscle sensitivity to insulin (59).

In the case of liraglutide treatment of females, despite short-term positive effects, long-term treatment turned out to be deleterious. Not only was glucose tolerance not significantly improved, but the treated females had the highest serum basal insulin levels, the most pronounced peripheral insulin resistance, and the largest adrenal glands (suggesting the most hyperactive stress response). The sex differences in liraglutide response could be partially explained by human data showing a 24% lower weight-adjusted clearance in women compared to men (60). In accordance with previous literature (48), our results suggested that animals treated with liraglutide had profound central effects of treatment. Glucagon-like peptide 1 receptor (GLP1R) was expressed on satiety nuclei in the brain, and therefore the central action of its agonist liraglutide was expected. Also, polyphagia and high glucose set-point in HFHSD+L females can be explained by the main peripheral action of liraglutide, stimulating insulin secretion and concomitant development of insulin resistance primarily in the satiety nuclei. The argument about insulin resistance of satiety nuclei was based on the reduced expression of IR, a phenomenon associated with hyperphagia (61). For final proof of insulin resistance, it would be necessary to perform a functional study—to apply insulin and determine the values of pAkt and pGSK3 in the tissue 1 h after insulin application (62). In addition, hyperinsulinemia was combined with a loss of protective leptin signaling due to a decrease in adipocyte surface area and a consequent decrease in their ability to excrete leptin and adiponectin. All these results suggested that the females treated with liraglutide have a high tendency to develop adipocyte insulin resistance—an inability to store lipids (or blocked lipolysis) and excrete leptin at sufficient levels to counteract central insulin resistance. Moreover, they concomitantly developed exaggerated stress responses. Sympathetic activation is demonstrated to promote the conversion of stored lipids into energy metabolism pathways (63). Intriguingly, the liraglutide-treated animals exhibited significantly smaller visceral adipocytes compared to other HFHSD-fed animals, which could also be due to the centrally dysregulated sympathetic activity.

Unlike males, liraglutide did not increase PA or phospholipid levels in female skeletal muscle, but it did increase xanthurenic acid 8-*O*-sulfate, which was expected to increase natriuresis (64) and consequent glucouresis. This may explain previously reported liraglutide-induced natriuresis that has not been mediated by natriuretic peptides (65). Given that xanthurenic acid interferes with the synthesis of insulin in β-cells and creates inactive complexes with insulin (47, 66, 67), it could be part of the last protective mechanism that acts against hyperinsulinemia and insulin resistance and reflects the severity of the diabetic disorder. In these circumstances, the development of insulin resistance in female skeletal muscle after liraglutide treatment could be considered a protective mechanism that saves energy-hungry muscle (loaded with mitochondria) from glucose loss in postprandial hypoglycemia that occurred after hyperinsulinemia following hyperphagia in conditions of gradual exhaustion of all energy stores.

We do not know if longer follow-up would result in a similar effect of liraglutide in males. It is worth noting that HFHSD+L males had the lowest glucose tolerance among all male groups, which meant that they had a significant loss of β-cells and were close to decompensation. Yet, liraglutide has its place in prediabetic therapy, especially in men, under the condition of personalized dosing and strict control of hyperinsulinemia.

### The strengths and limitations of the study

The strength of our study is that it included animals of both sexes, the use of a mathematical model that sheds light on the sequence of metabolic deterioration from prediabetes to diabetes, and finally whole-body analysis. In translational studies, the female gender is less represented due to difficulties in achieving synchronization in the estrous cycle (typical for rats) and the expectation of large variations in biochemical parameters influenced by sex hormones. Recent studies show that the variation among females (unstaged for cycle) is not greater than the variation among males (68), so excluding females is one of the obstacles to gender-sensitive personalized medicine. The use of mathematical modeling is still rare in biological studies, although it can reveal parameters that have a higher sensitivity than those used in clinical practice. Finally, whole-body analysis is complex, but it can reveal the mutual connection of pathophysiological mechanisms.

We recognize the following limitations of our study: (1) variability in female animals could be smaller if animals were synchronized for their estrus cycle (which was omitted because of animal age, duration of the study, and low variability among females, independent of cycle stage); (2) due to the high number of animals handled at the same time points, even if experiments were performed at the same time of day, certain parameters (like hormones) could be influenced by the circadian rhythm; (3) handling of animals, even if done with special care and by properly trained technicians, could be a source of stress that could be avoided by using metabolic cages and continuous glucose monitoring (which were unavailable due to limited resources); (4) dosage of treatment medications was calculated based on the current human therapy guidelines; however, other doses should also be tested; (5) murine models are not well tested for metabolic syndrome in aged animals, and female predisposition toward development of diabetes might be strain-dependent; (6) assessment of insulin/leptin resistance at the level of the hypothalamic nuclei or skeletal muscle would be more precise if it was done within the framework of a functional study, i.e., after insulin/leptin administration followed by assessment of GLUT4 and STAT3 expression; (7) in addition to IRS-1 phosphorylation, GLUT4 translocation should be determined 30–60 min after insulin challenge; and (8) the use of additional methods directed at whole-body changes in mitochondrial function (indirect whole-body calorimetry) should be recommended. The finding of liraglutide side effects was unexpected but was reproduced in a replicated study (data not yet published).

### Prediabetic interventions should start earlier and become sex-specific

In conclusion, the pathophysiology of DM2 is very complex and requires the monitoring of several clinical parameters instead of focusing solely on insulin insensitivity. A plethora of impairments in many different tissues and organ systems contribute to DM2 in various degrees, including the liver (impaired carbohydrate metabolism, hyperreactive gluconeogenesis), skeletal muscle (impaired glucose uptake and energy metabolism), adrenal glands (impaired stress responses), adipose tissue (impaired secretion of adipocytokines), brain (impaired central regulation of energy homeostasis and stress reaction), and possibly many other organs (69, 70). While this study undoubtedly confirmed the complexity of multiple organ involvement in the development and progression of DM2, it also shed light on the role of sex differences, aging, and the relative contribution of various organs or tissues to disease severity. Moreover, significant sex differences were noted even in response to antidiabetic medication, which was strikingly obvious from the MALDI-TOF skeletal muscle analysis of the treated animals. Although neither of the two antidiabetic drugs used as prediabetes treatment, metformin and liraglutide, were able to reverse HFHSD-induced DM2, metformin was the superior intervention over liraglutide due to improved central leptin sensitivity and peripheral insulin sensitivity in females. The short-term success and long-term failure of liraglutide therapy can be explained by its central effect on satiety nuclei and hyperinsulinemia, which ultimately lead to insulin resistance. Thanks to its positive effects on the metabolism of skeletal muscle, liver, and adipocytes, with good titration, liraglutide can find its place, especially in the treatment of males. If some future prediabetic therapy is to be considered, this study suggests that its success will depend on the correct identification of early biomarkers of prediabetes and, equally early, the application and monitoring of a well-targeted sex-specific approach.

## Methods

### Animal model and study design

The animal study was approved by the National Scientific Ethical Committee on Animal Experimentation (Hungary, registration number: IV/3084/2016). The animals were treated in accordance with the European Communities Council Directives (86/609/ECC) and the Hungarian Act for the Protection of Animals in Research (XXVIII. tv. 32.§).

The study was carried out on 32 male and 32 female Sprague–Dawley rats (Innovo Ltd, Gödöllő, Hungary). Three-week-old rats were fed *ad libitum* with standard rodent chow (Innovo Ltd, Gödöllő, Hungary) and water. They were kept in cages in a room with controlled temperature (20°C–23°C), humidity (40%–60%), and light/dark cycle (12 h light/12 h dark). STD consisted of 65% carbohydrate (5% disaccharide, 39% polysaccharide), 11% fat, and 24% protein. When the rats reached 45 weeks of age (week 0 of the experiment), they were randomly separated into four groups, each composed of eight males and eight females: (1) STD, (2) HFHSD, (3) HFHSD+M, and (4) HFHSD+L. The STD group continued consuming standard food until the end of the experiment, while others were transferred to HFHSD (Altromin Spezialfutter GmbH & Co, Lage, Germany, Cat. No. C-1101) consisting of 56% carbohydrate (18% disaccharide, 36% polysaccharide), 28% fat, and 16% protein. From experimental week 6 (when the rats were 51 weeks old), HFHSD+M group was treated subcutaneously with 50 mg/kg/day of metformin [resuspended in sterile/distilled water (50 mg/mL)]; Sigma Aldrich, Budapest, Hungary) and the HFHSD+L group was injected subcutaneously with 0.3 mg/kg/day of liraglutide (resuspended in sterile/distilled water (50 mg/mL)); Creative Peptides Inc., Shirley, NY, USA). STD and HFHSD groups were administered with vehicle (sterile/distilled water) only. The dose was determined according to reports in the literature (71, 72). Antidiabetic treatment lasted for 13 weeks, until the end of the experiment when rats reached 64 weeks of age.

Body mass and food consumption were measured weekly (every morning of the first day of each experimental week) using a digital scale (SPX621, Ohaus Corp., Parsippany, NJ, USA). Food consumption was calculated weekly by the rodent pellet reduction in the feeder rack of cages and expressed as caloric intake (kcal/g of body mass) per animal group. The metabolic energy of the STD was 2.84 kcal/g and of the HFHSD was 3.89 kcal/g.

During the experiment, several animals succumbed, as follows: three males and one female from the STD group died during or after the GTT due to aortic aneurysm; one female from the HFHSD+M group died due to pulmonary edema; and one female from the HFHSD+L group died due to abdominal tumor and vaginal bleeding.

At the end of the experiment, animals were sacrificed during deep isoflurane anesthesia (Forane) (Baxter Healthcare Corp. Deerfield, IL, USA) by cardiac puncture, followed by the collection of whole blood. Prepared serum and plasma samples were stored at −20°C for later analysis. The organs (brain, liver, adrenal glands, adipose tissue) were weighed, snap frozen, or fixed with 4% paraformaldehyde, as previously described (73), and stored at −80°C for further molecular studies. Nuchal region skeletal muscles (*Semispinalis capitis*, *Splenius capitis*, and *Splenius cervicis*) were cryoprotected and embedded in methylcellulose that does not interfere with iMS. Histology was performed on fixed cryoprotected sections, and iMS on fresh frozen. Part of the tissue necessary for histological staining was embedded in paraffin (adipose tissue, adrenal glands, liver). Liver mass to body mass ratio was calculated and shown as a percentage.

### Glucose and insulin tolerance tests

The GTT was carried out four times, as follows: at the beginning of the experiment (week 0), immediately before beginning treatments with antidiabetics (metformin, liraglutide; week 5), after 6 weeks of treatments (week 12), and the week before animals were sacrificed (week 18). Animals fasted for 16 h before the GTT. Fasting glucose level was measured first, followed by intraperitoneally injection of glucose solution (25%) at a 2-mg/kg dose. Blood glucose levels were determined 15, 30, 45, 60, 90, 120, and 240 min after the injection. Blood samples were obtained from a tail vein using a needle to collect one drop of blood (5 µL) and place it on a test strip. Blood glucose levels were determined using a glucometer (OneTouch UltraMini, Milpitas, CA, USA), and glucose concentration curves were plotted. The ITT was carried out during the week the animals were sacrificed (week 18). Animals fasted for 4 h before the ITT. The fasting glucose level was measured first, and then each rat was injected intraperitoneally with 0.5 U/kg of Humulin R insulin (Eli Lilly, Indianapolis, IN, USA). Blood glucose levels were determined 15, 30, 45, 60, 90, 120, and 180 min after the injection.

In addition to calculating the AUC, the measurements were also used for plotting the model function of glucose concentration during GTT and ITT. AUC determination and modeling were performed as previously described (35). NonlineraModelFit module of Mathematica (ver. 11.0, Wolfram Research, Inc., Champaign, IL, USA) was used to solve functions describing glucose concentration fluctuation during GTT and ITT. The following obtained parameters revealed alterations in glucose dynamics: fasting blood glucose concentration (*G*(0) in GTT/H(0) in ITT), maximal/minimal glucose concentration (*G*_max_/*H*_min_) and corresponding moment (*t*_max_/*t*_min_), 2-h blood glucose (*G*(2)), coefficient of oscillation amplitude decline (α), basic period of function (*T*), blood glucose setpoint (*G*_0_/*H*_0_), initial speed of blood glucose increase/decrease (*G*′(0))/(*H*′(0)), blood glucose concentration at which maximal speed of glucose concentration decrease/increase is attained (*G*_I_), maximal speed of glucose concentration decrease (*G*′_I_) and corresponding moment (*t*_I_). To evaluate the goodness-of-fit of the model, a determination coefficient (*R* ^2^) was calculated.

### Tissue, serum, and plasma measurements

Serum fasting insulin was measured using a Rat Ultrasensitive Insulin ELISA kit (ALPCO, Salem, NH, USA). Plasma leptin, adiponectin, corticosterone, and TNF-α, IL-1, and IL-6 from subcutaneous and visceral adipose tissues were measured using appropriate ELISA kits from R&D Systems (Minneapolis, MN, USA). Phosphorylated IRS-1 from the liver, visceral adipose tissue, and muscles were measured using a Phospho-IRS-1 (panTyr) ELISA kit (Cell Signaling Technology, Danvers, MA, USA). The enzymes AST and ALT were measured using standard clinical laboratory methods. HOMA-IR was calculated using insulin and glucose data with equation HOMA-IR = {fasting glucose (mg/dL) × fasting insulin (µU/mL)}/405.

### Adipose tissue histomorphometry

Visceral adipocyte surface areas were measured based on hematoxylin and eosin histological staining (HE) of 5-µm-thick sections of paraffin-embedded tissue using following protocol (xylene for 10 min; 100% ethanol (EtOH), 100% EtOH, 96% EtOH, 70% EtOH for 5 min each; distilled water (dH_2_O) for 5 min; Mayer’s hematoxylin for 10 min; dH_2_O for 1 min; tap water for 10 min; dH_2_O for 1 min; eosin Y for 30 s; dH_2_O for 5 s; 70% EtOH, 96% EtOH, 100% EtOH at 5 dips in each, and 100% EtOH for 3 min; xylene for 5 min and covesliped). Digital micrographs, collected by an Olympus D70 camera (Olympus, Hamburg, Germany) set up on a Zeiss Axioskop 2 MOT microscope (Carl Zeiss Microscopy, Thornwood, NY, USA), were analyzed in CellProfiler (v. 3.1.9) using a semiautomated protocol consisting of several modules (74). Each micrograph was split into three color channels. The green channel was converted to a grayscale image for subsequent analysis. In the next module, the adipocytes were identified as primary objects based on their typical diameter and intensity range, as determined manually for each micrograph and using a global threshold strategy, which classifies the pixels above the threshold as foreground (i.e., adipocytes) and below as background. Otsu’s algorithm was used as a thresholding method because the percentages of areas covered by the foreground varied. Objects touching the borders of the images were discarded from further analysis. When a single adipocyte was identified as two or more objects due to the intensity gradient, the Split or Merge Objects module was applied, using the distance-based merging method. Finally, the surface areas of the identified adipocytes were measured in pixels and converted to square micrometers using the scale bars of the original micrographs. Furthermore, surface areas were divided into four distinct adipocyte size classes, based on the distribution of median values and upper and lower quartiles in STD groups: class 1 (< 2,197.5 µm²), class 2 (2,197.5–4,395 µm²), class 3 (4,395–6,592 µm²), and class 4 (> 6,592 µm²).

### Adrenal gland histopathology

Paraffin-embedded adrenal glands were cut using a microtome (Leica SM2000R; Nussloch, Germany) into 5-µm-thick sections. The HE staining of tissue was followed by a collection of digital micrographs at ×100 magnification.

### Analysis of liver fat droplets, glycogen, and ferric ion

Liver tissue was cut using a cryostat (Leica CM3050S; Nussloch, Germany) into 20-µm-thick sections. Liver fat droplets were stained using Sudan Black B to show overall fat distribution and Oil Red O for quantification, as described by Vacca (75). Digital micrographs were collected at 200× magnification. Fat droplet size determination was performed using FIJI software (76). Images were split into red, green, and blue channels and converted into 8-bit images. The red color threshold was set at 0-100, and the surface area of the remaining particles was analyzed. The size of fat droplets was expressed in square micrometers.

Liver glycogen was stained using a metachromatic toluidine stain, as described by Vacca (75). Digital micrographs were collected at 400× magnification. The images were deconvoluted with a Feulgen light green vector in FIJI software, and Color 1 was used to measure integrated density value (IDV). Glycogen values are presented as positively correlated integrated color density (the number obtained from quantification was subtracted from maximal IDV, which corresponds to the total pixel number of an image multiplied by 255).

### Skeletal muscle analyses

Paraformaldehyde-fixed skeletal muscle tissue from nuchal region tissue was cut using a cryostat into 35-µm-thick sections. Skeletal muscle fat droplets were stained using Oil Red O, as described by Vacca (75). Digital micrographs were collected at 200× magnification and analyzed using FIJI software. Images were split into red, green, and blue channels. In order to characterize fat droplets, the green channel threshold was set at 0–140, and the remaining particles were segmented by *binary/watershed*. The resulting particles were analyzed with a lower limit of 25 square pixels. The fat droplet size is presented in square pixels. To determine the number of muscle fibers in a section, edges were detected in a green channel, whose threshold was set at 0–35. With interior holes included, the remaining particles were analyzed with a lower limit of 2,000 square pixels. To obtain an average count of fat droplets per fiber, the total number of droplets per section was divided by the number of muscle fibers in a given section.

To determine skeletal muscle fiber type composition, snap-frozen skeletal muscles were cut using a cryostat into 14-µm-thick sections. Slices were stained with succinate dehydrogenase, as described by Vacca (75). Digital micrographs were collected at 200× magnification and analyzed using FIJI software. Images were split into red, green, and blue channels. Only a transversely cut area of the sample was selected for further analysis. The selected region was duplicated, the background was subtracted, and segmentation was performed using the Statistical Region Merging algorithm. Q was set to 4: background and three muscle fiber types (I, IIa, IIb). To quantify areas of the specific muscle types, the image threshold was set at 0–120 for type I fibers, 121–180 for type IIa fibers, 181–240 for type IIb fibers, and 0–240 for total area. The protocol was adjusted to conform to the muscle fiber classification described by Kano et al. (77, 78).

Analysis of snap-frozen skeletal muscle tissue was used to determine lipid peroxidation (LPO), total glutathione (tGSH), and the activities of the following antioxidant enzymes: glutathione reductase (GR), glutathione *S*-transferase (GST), catalase (CAT), and superoxide dismutase (SOD).

LPO was estimated by measuring the thiobarbituric acid reactive substances (TBARS), according to the method described by Ohkawa et al. (79) The TBARS were calculated according to a standard curve prepared from 1,1,3,3-tetraethoxypropane and expressed in nanomoles per milligram of fresh tissue weight (nmol/mg FW).

tGSH content was assayed using a spectrophotometric kinetic method based on a reduction of 5,5-dithiobis(2-nitrobenzoic acid) (DTNB) to 5-thio-2-nitrobenzoic acid by glutathione (GSH), recorded at 412 nm and expressed in nmol/mg FW (80).

Protein extracts (1:10, w/v) were prepared for the antioxidant enzyme activity assay by homogenizing tissue in 100 mM phosphate buffer (pH 7.0) containing 1 mM EDTA and by centrifugation at 20,000×*g* for 15 min at 4°C. Protein concentration in extracts was estimated using the Bradford assay (81).

GR activity was determined indirectly by measuring the consumption of NADPH during GSSG reduction, demonstrated by a decrease in absorbance at 340 nm. The assay mixture (1 mL) consisted of 1 mM GSSG, 0.1 mM NADPH, and protein extract in 100 mM phosphate buffer (pH 7.5). The GR activity was calculated using a molar extinction coefficient for NADPH (ϵ = 6.220 mM/cm) and expressed in units per gram of (U/g) protein (82).

GST activity was determined by measuring the conjugation of 1-chloro-2,4-dinitro benzene (CDNB) with GSH, demonstrated by an increase in absorbance at 340 nm (83). The GST activity was calculated using a molar extinction coefficient of glutathione-1-chloro-2,4-dinitrobenzene conjugate (ϵ = 9.6 mM/cm) and expressed in U/g protein.

CAT activity was estimated spectrophotometrically using H_2_O_2_ as a substrate, as described by Aebi (84) and expressed in U/g protein.

SOD activity was determined by measuring the inhibition of cytochrome c reduction with superoxide radicals generated by the xanthine/xanthine oxidase system. The reduction rate was recorded spectrophotometrically at 550 nm (85). Results were expressed in units per milligram of proteins, where one unit of SOD activity was defined as the amount of enzyme that caused 50% inhibition of cytochrome c reduction under the assay.

### MALDI-TOF skeletal muscle analysis

MALDI-TOF IMS analysis was performed using the Shimadzu IMScope TRIO MALDI-IT-TOF MS instrument (Shimadzu, Kyoto, Japan). Fresh-frozen nuchal skeletal muscle tissue sections (25 μm thick) were mounted on an indium tin oxide (ITO)-coated glass slide, with a surface resistivity of 15–25 Ω/sq (Sigma-Aldrich, St. Louis, MO, USA). After snap washing with 20 mM ammonium acetate buffer, the sections were dried and immediately further processed. Matrix α-cyano-4-hydroxycinnamic acid (CHCA) (Sigma-Aldrich, St. Louis, MO, USA) was applied to samples using an iMLayer sublimation device (Shimadzu, Kyoto, Japan) according to the manufacturer’s instructions (10 min sublimation at 180°C). Sublimation was followed by 2 min of recrystallization at 70°C with 0.5% methanol in a vapor chamber.

Imaging in the positive ion mode was performed using m/z ranges 300–700 and 700–1,000 Da with the following setup: approximately 500 pixels with a pitch of 10 × 10 µm, a laser diameter of 10 µm, a laser intensity of 15%, 50 laser shots/pixel, and a 200-Hz laser frequency. Data analysis was performed with R software (86) ver. 4.2.0 (R Foundation for Statistical Computing, Vienna, Austria). Total-ion-current (TIC)-normalized m/z signals were used for the image generation and data analysis. Only the strong, TIC-normalized m/z signals averaged over all pixels were used in the statistical analysis; strong m/z signals were the ones for which the sum of intensities across all images (∑Im/z) was greater than 5% of the largest ∑Im/z. For the selection of significant m/z signals, a FDR-corrected KW ANOVA followed by pairwise Dunn–Bonferroni test was used. Graphical presentation of the IMS results was performed by ImageReveal ver. 1.1.010128 (Shimadzu, Kyoto, Japan). Human metabolome database (HMDB) (87) and METASPACE database (88) (https://metaspace2020.eu) were used for the tentative metabolite identification. ± 10 ppm m/z accuracy tolerance and a statistical significance cutoff of 0.05 were used in all instances.

### Immunohistochemistry

Macrophages of M1 and M2 phenotypes were immunostained on 5-µm-thick sections of visceral adipose tissue and 20-µm-thick cryosections of the liver. IR-α, ObR, IGF-1Rβ, Iba1, and GFAP were immunohistochemically stained on 35-µm-thick coronal brain cryosections.

Slide-mounted sections of visceral adipose tissue were stained using the VENTANA Benchmark Ultra IHC/ISH System (Roche, Basel, Switzerland) and anti-CD68 (clone KP-1; Roche) and anti-CD163 (clone MRQ-26; Roche) antibodies.

Liver and brain tissues were stained by free-floating immunohistochemistry developed with 3,3′-diaminobenzidine (DAB) as previously described by our group at 4°C and without detergents applied. The following antibodies were used: rabbit anti-CD197 diluted 1:1,000 (Abcam, Cambridge, UK) and rabbit anti-CD206 diluted 1:1,000 (Abcam) for liver tissue; rabbit anti-alpha subunit of IR-α diluted 1:250 (IR-α; Santa Cruz Biotechnology, Dallas, TX, USA; SC-710), rabbit anti-beta subunit of IGF-1Rβ diluted 1:250 (IGF-1Rβ; Santa Cruz Biotechnology; SC-713), rabbit anti-ObR diluted 1:50 (ObR; Santa Cruz Biotechnology; SC-8325), and biotinylated goat anti-rabbit IgG diluted 1:1,000 (Jackson ImmunoResearch Laboratories, Inc. West Grove, PA, USA) for brain tissue.

GFAP and Iba1 expressions were analyzed by free-floating fluorescent immunohistochemistry using the same protocol described above. After the incubation with secondary antibodies, the sections were incubated for 10 min at room temperature with 0.1% Sudan Black B prepared in 70% ethanol to suppress autofluorescence. Afterward, the sections were shortly rinsed with distilled water, slide-mounted, and coverslipped with Vectashield with 4′,6-diamidino-2-phenylindole (DAPI) (Vector Laboratories, Burlingame, CA, USA). Rabbit anti-GFAP diluted 1:4,000 (Dako, Agilent Technologies, Santa Clara, CA, USA), rabbit anti-Iba1 diluted 1:1,000 (Wako Chemicals, Neuss, Germany), and goat anti-rabbit IgG conjugated with Cy3 diluted 1:300 (Jackson ImmunoResearch Laboratories, Inc., West Grove, PA, USA) were used.

The digital micrographs of DAB-developed staining of visceral adipose tissue and liver tissue were collected at ×200 magnification. Brain tissue digital micrographs were collected at ×400 magnification from hypothalamic areas associated with energy maintenance: ARC, LH, PVN, and VMH. Immunopositive macrophages were counted per field of view. IR-α, IGF-1Rβ, and ObR immunopositive reactions within the areas of 0.02 mm^2^ were analyzed using FIJI software by the following steps: images were converted to 8-bit, the threshold was set to omit background (0–127 till 142), and the corresponding color area of immunopositive reaction or IDV was measured. ROIs on fluorescent micrographs (areas of five glial cells per image) were analyzed using the Color Pixel Counter plugin for FIJI with a minimum intensity value set to 30 after enhancing local contrast (block size: 127, histogram bins: 256, maximum slope: 3.00, mask: none) and adjusting the gamma value to 1.80.

### Statistical analysis

Statistical analysis was performed using Statistica 12 software (TIBCO, Palo Alto, CA, USA). The statistical significance level was set at *p* < 0.05. For significance analysis of between-group comparisons to determine the influence of sex, intervention, or their interaction, two-way ANOVA was applied followed by Bonferroni or Games–Howell test depending on the equality of variances assessed by Levene’s test. To assess the influence of sex, intervention, and duration of intervention on the ratio of the whole-group caloric intake to the whole-group body mass, repeated measures of three-way ANOVA were applied, and the Bonferroni *post-hoc* test was performed. The statistical analysis of MALDI-TOF was performed as mentioned above.

## Data availability statement

The datasets presented in this study can be found in online repositories. The names of the repository/repositories and accession number(s) can be found in the article/**Supplementary Material**.

## Ethics statement

The animal study was approved by National Scientific Ethical Committee on Animal Experimentation (Hungary, registration number: IV/3084/2016). The study was conducted in accordance with the local legislation and institutional requirements.

## Author contributions

The authors confirm contribution to the paper as follows: Study conception and design: SV, RG, MH, TT, and SG. Data collection: VI, MZ, MF, KS, FB, SB, RV, AR, DM, ZD, MBer, MBal, AI, MBak, AS-B, and SM. Analysis and interpretation of results: VI, MZ, IL, MH, MF, RS, SB, AR, DM, ZD, MBer, MBal, and SM. Draft manuscript preparation: VI, MF, MH, SB, AR, SG, RG, SV, and MZ. All authors contributed to the article and approved the submitted version.
