# Supplementary material for: Elderly rats fed with a high-fat high-sucrose diet developed sex-dependent metabolic syndrome regardless of long-term metformin and liraglutide treatment
Source: Front Endocrinol (Lausanne). 2023 Oct 20;14:1181064. doi: 10.3389/fendo.2023.1181064 (PMC10623428; doi:10.3389/fendo.2023.1181064)
Supplement: Supplementary file 1 [file DataSheet_1.zip › Extended Data/Extended Data Fig. 4.PPTX]

## Slide 1
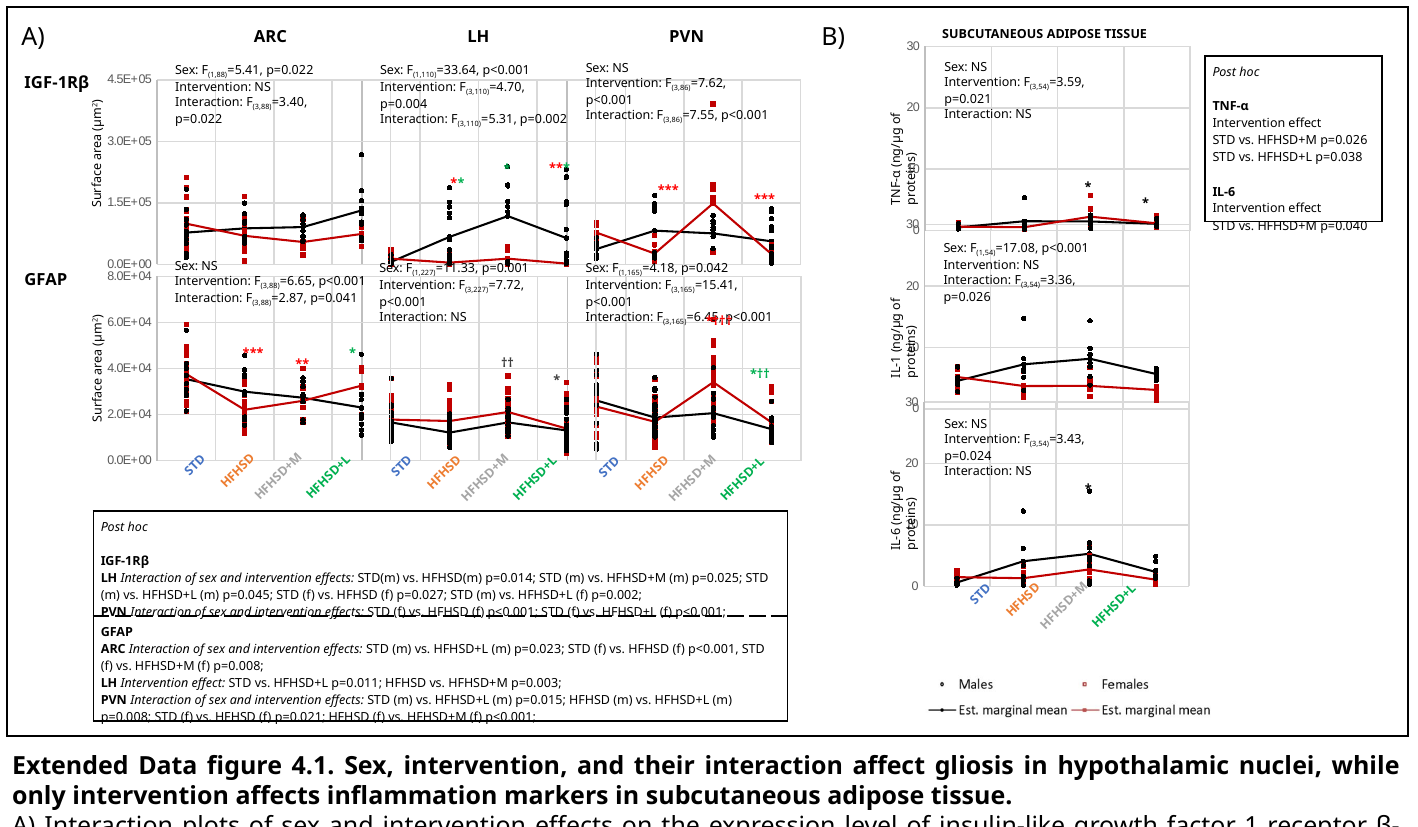

A)
B)
ARC
LH
PVN
SUBCUTANEOUS ADIPOSE TISSUE
### Chart
| Category | Mean Males | Mean Females | Males1 | Males2 | Males3 | Males4 | Males5 | Males6 | Males7 | Males8 | Females1 | Females2 | Females3 | Females4 | Females5 | Females6 | Females7 | Females8 |
|---|---|---|---|---|---|---|---|---|---|---|---|---|---|---|---|---|---|---|
| SD | 0.5457142857142857 | 0.6483333333333333 | 1.13 | 0.48 | 0.37 | 0.48 | 0.57 | 0.27 | 0.52 | None | 0.39 | 0.54 | 0.39 | 1.24 | 0.36 | 0.97 | None | None |
| HFHSD | 1.5466666666666666 | 0.57375 | 5.36 | 1.19 | 0.75 | 0.28 | 1.08 | 0.62 | None | None | 0.8 | 1.02 | 0.99 | 0.5 | 0.28 | 0.51 | 0.24 | 0.25 |
| HFHSD+M | 1.5024999999999997 | 2.2814285714285716 | 1.08 | 2.55 | 1.9 | 2.4 | 1.86 | 0.37 | 1.5 | 0.36 | 0.78 | 0.53 | 3.41 | 3.52 | 5.76 | 1.49 | 0.48 | None |
| HFHSD+L | 1.11625 | 1.1960000000000002 | 2.04 | 0.76 | 0.98 | 0.72 | 0.9 | 1.8 | 0.9 | 0.83 | 2.53 | 0.85 | 0.52 | 0.89 | 1.19 | None | None | None |Sex: NS
Intervention: F(3,54)=3.59, p=0.021
Interaction: NS
Sex: NS
Intervention: F(3,86)=7.62, p<0.001
Interaction: F(3,86)=7.55, p<0.001
Sex: F(1,88)=5.41, p=0.022
Intervention: NS
Interaction: F(3,88)=3.40, p=0.022
Sex: F(1,110)=33.64, p<0.001
Intervention: F(3,110)=4.70, p=0.004
Interaction: F(3,110)=5.31, p=0.002
| Post hoc TNF-α Intervention effect STD vs. HFHSD+M p=0.026 STD vs. HFHSD+L p=0.038 IL-6 Intervention effect STD vs. HFHSD+M p=0.040 |
| --- |
IGF-1Rβ
### Chart
| Category | MALES-AVERAGE | FEMALES-AVERAGE | M1 | M2 | M3 | M4 | M5 | M6 | M7 | M8 | M9 | M10 | M11 | M12 | M13 | M14 | M15 | M16 | M17 | M18 | M19 | M20 | M21 | M22 | M23 | M24 | M25 | M26 | M27 | M28 | M29 | M30 | M31 | M32 | M33 | M34 | M35 | M36 | F1 | F2 | F3 | F4 | F5 | F6 | F7 | F8 | F9 | F10 | F11 | F12 | F13 | F14 | F15 | F16 | F17 | F18 | F19 | F20 | F21 | F22 | F23 | F24 | F25 | F26 | F27 | F28 | F29 | F30 | F31 | F32 | F33 | F34 | F35 | F36 |
|---|---|---|---|---|---|---|---|---|---|---|---|---|---|---|---|---|---|---|---|---|---|---|---|---|---|---|---|---|---|---|---|---|---|---|---|---|---|---|---|---|---|---|---|---|---|---|---|---|---|---|---|---|---|---|---|---|---|---|---|---|---|---|---|---|---|---|---|---|---|---|---|---|---|---|
| SD | 77187.64166666666 | 99170.05882352941 | 50933.84 | 69197.99 | 183303.0 | 80799.55 | 61200.58 | 74328.19 | 26936.87 | 16720.7 | 22674.01 | 134247.0 | 109838.5 | 96071.47 | None | None | None | None | None | None | None | None | None | None | None | None | None | None | None | None | None | None | None | None | None | None | None | None | 90341.0 | 163976.0 | 165539.0 | 69542.0 | 24743.0 | 187152.0 | 134530.0 | 212556.0 | 63900.0 | 80245.0 | 31617.0 | 71407.0 | 41343.0 | 50181.0 | 57301.0 | 130080.0 | 111438.0 | None | None | None | None | None | None | None | None | None | None | None | None | None | None | None | None | None | None | None |
| HFHSD | 87635.35222222222 | 69413.35714285714 | 123329.0 | 149347.1 | 68058.78 | 51327.13 | 59684.43 | 99806.86 | 66379.36 | 66156.11 | 104629.4 | None | None | None | None | None | None | None | None | None | None | None | None | None | None | None | None | None | None | None | None | None | None | None | None | None | None | None | 76952.0 | 106241.0 | 48398.0 | 42041.0 | 30134.0 | 8011.0 | 58578.0 | 117633.0 | 37337.0 | 39431.0 | 61664.0 | 83309.0 | 97337.0 | 164721.0 | None | None | None | None | None | None | None | None | None | None | None | None | None | None | None | None | None | None | None | None | None | None |
| HFHSD+M | 90720.98300000001 | 54347.25 | 67607.23 | 120182.4 | 118805.7 | 80816.12 | 55849.45 | 108238.5 | 108685.0 | 81508.84 | 66662.32 | 98854.27 | None | None | None | None | None | None | None | None | None | None | None | None | None | None | None | None | None | None | None | None | None | None | None | None | None | None | 108983.0 | 37990.0 | 109652.0 | 21023.0 | 47738.0 | 23218.0 | 47738.0 | 38436.0 | None | None | None | None | None | None | None | None | None | None | None | None | None | None | None | None | None | None | None | None | None | None | None | None | None | None | None | None |
| HFHSD+L | 131314.523 | 73923.44444444444 | 57511.87 | 65449.61 | 98082.55 | 267345.1 | 155158.0 | 127103.1 | 179231.7 | 136851.6 | 102955.0 | 123456.7 | None | None | None | None | None | None | None | None | None | None | None | None | None | None | None | None | None | None | None | None | None | None | None | None | None | None | 63245.0 | 63906.0 | 69896.0 | 72742.0 | 96778.0 | 99978.0 | 88853.0 | 43087.0 | 66826.0 | None | None | None | None | None | None | None | None | None | None | None | None | None | None | None | None | None | None | None | None | None | None | None | None | None | None | None |
### Chart
| Category | MALES-AVERAGE | FEMALES-AVERAGE | M1 | M2 | M3 | M4 | M5 | M6 | M7 | M8 | M9 | M10 | M11 | M12 | M13 | M14 | M15 | M16 | M17 | M18 | M19 | M20 | M21 | M22 | M23 | M24 | M25 | M26 | M27 | M28 | M29 | M30 | M31 | M32 | M33 | M34 | M35 | M36 | F1 | F2 | F3 | F4 | F5 | F6 | F7 | F8 | F9 | F10 | F11 | F12 | F13 | F14 | F15 | F16 | F17 | F18 | F19 | F20 | F21 | F22 | F23 | F24 | F25 | F26 | F27 | F28 | F29 | F30 | F31 | F32 | F33 | F34 | F35 | F36 |
|---|---|---|---|---|---|---|---|---|---|---|---|---|---|---|---|---|---|---|---|---|---|---|---|---|---|---|---|---|---|---|---|---|---|---|---|---|---|---|---|---|---|---|---|---|---|---|---|---|---|---|---|---|---|---|---|---|---|---|---|---|---|---|---|---|---|---|---|---|---|---|---|---|---|---|
| SD | 5659.38 | 13553.5 | 4483.35 | 5438.82 | 1469.95 | 887.5 | 2699.48 | 1183.33 | 1580.2 | 551.23 | 1359.71 | 22883.02 | 13357.73 | 12018.24 | None | None | None | None | None | None | None | None | None | None | None | None | None | None | None | None | None | None | None | None | None | None | None | None | 18492.0 | 11832.0 | 18418.0 | 19199.0 | 26529.0 | 19348.0 | 740.0 | 851.0 | 666.0 | 814.0 | 2145.0 | 1516.0 | 10280.0 | 5029.0 | 15753.0 | 21395.0 | 35720.0 | 35236.0 | None | None | None | None | None | None | None | None | None | None | None | None | None | None | None | None | None | None |
| HFHSD | 66702.012 | 4003.285714285714 | 4152.61 | 3858.62 | 2425.42 | 10095.32 | 12609.91 | 8911.99 | 59831.42 | 23513.22 | 35120.07 | 113373.3 | 139791.1 | 186561.7 | 124312.5 | 151734.9 | 124238.1 | None | None | None | None | None | None | None | None | None | None | None | None | None | None | None | None | None | None | None | None | None | 2095.0 | 845.0 | 2866.0 | 698.0 | 6.0 | 74.0 | 4093.0 | 7702.0 | 1823.0 | 30.0 | 186.0 | 32.0 | 2270.0 | 484.0 | 2084.0 | 147.0 | 220.0 | 220.0 | 28985.0 | 10679.0 | 18530.0 | None | None | None | None | None | None | None | None | None | None | None | None | None | None | None |
| HFHSD+M | 117583.86777777776 | 13560.222222222223 | 191138.3 | 238243.9 | 193705.7 | 127735.6 | 153520.9 | 140423.6 | 4740.6 | 6431.04 | 2315.17 | None | None | None | None | None | None | None | None | None | None | None | None | None | None | None | None | None | None | None | None | None | None | None | None | None | None | None | 1079.0 | 3609.0 | 2567.0 | 558.0 | 2009.0 | 3535.0 | 32743.0 | 41934.0 | 34008.0 | None | None | None | None | None | None | None | None | None | None | None | None | None | None | None | None | None | None | None | None | None | None | None | None | None | None | None |
| HFHSD+L | 63959.85555555555 | 1589.111111111111 | 231844.0 | 211007.5 | 213798.1 | 145335.1 | 84499.73 | 152479.0 | 4316.15 | 3609.19 | 2158.07 | 1543.45 | 9076.95 | 5659.32 | 21915.61 | 27199.16 | 19757.53 | 3758.02 | 4241.73 | 9078.79 | None | None | None | None | None | None | None | None | None | None | None | None | None | None | None | None | None | None | 514.0 | 21.0 | 0.0 | 1749.0 | 1972.0 | 1265.0 | 2828.0 | 2902.0 | 3051.0 | None | None | None | None | None | None | None | None | None | None | None | None | None | None | None | None | None | None | None | None | None | None | None | None | None | None | None |
### Chart
| Category | MALES-AVERAGE | FEMALES-AVERAGE | M1 | M2 | M3 | M4 | M5 | M6 | M7 | M8 | M9 | M10 | M11 | M12 | M13 | M14 | M15 | M16 | M17 | M18 | M19 | M20 | M21 | M22 | M23 | M24 | M25 | M26 | M27 | M28 | M29 | M30 | M31 | M32 | M33 | M34 | M35 | M36 | F1 | F2 | F3 | F4 | F5 | F6 | F7 | F8 | F9 | F10 | F11 | F12 | F13 | F14 | F15 | F16 | F17 | F18 | F19 | F20 | F21 | F22 | F23 | F24 | F25 | F26 | F27 | F28 | F29 | F30 | F31 | F32 | F33 | F34 | F35 | F36 |
|---|---|---|---|---|---|---|---|---|---|---|---|---|---|---|---|---|---|---|---|---|---|---|---|---|---|---|---|---|---|---|---|---|---|---|---|---|---|---|---|---|---|---|---|---|---|---|---|---|---|---|---|---|---|---|---|---|---|---|---|---|---|---|---|---|---|---|---|---|---|---|---|---|---|---|
| SD | 37037.43 | 77440.75 | 20983.56 | 14111.54 | 23041.5 | 64518.95 | 52537.92 | 47031.11 | None | None | None | None | None | None | None | None | None | None | None | None | None | None | None | None | None | None | None | None | None | None | None | None | None | None | None | None | None | None | 73300.0 | 94769.0 | 73040.0 | 62547.0 | 70770.0 | 94472.0 | 48295.0 | 77878.0 | 62976.0 | 95998.0 | 74661.0 | 100583.0 | None | None | None | None | None | None | None | None | None | None | None | None | None | None | None | None | None | None | None | None | None | None | None | None |
| HFHSD | 81913.08916666667 | 26230.866666666665 | 19182.87 | 15397.75 | 18852.13 | 20819.29 | 28141.17 | 24776.06 | 135288.9 | 167734.4 | 142135.2 | 129744.9 | 133242.4 | 147642.0 | None | None | None | None | None | None | None | None | None | None | None | None | None | None | None | None | None | None | None | None | None | None | None | None | 23181.0 | 10976.0 | 20874.0 | 31590.0 | 13432.0 | 5730.0 | 46696.0 | 69244.0 | 48668.0 | 30101.0 | 25897.0 | 27311.0 | 14994.0 | 14259.0 | 10510.0 | None | None | None | None | None | None | None | None | None | None | None | None | None | None | None | None | None | None | None | None | None |
| HFHSD+M | 75304.93222222223 | 148438.33333333334 | 105336.3 | 89522.83 | 68016.52 | 37594.02 | 37888.01 | 37814.52 | 83383.49 | 118768.5 | 99420.2 | None | None | None | None | None | None | None | None | None | None | None | None | None | None | None | None | None | None | None | None | None | None | None | None | None | None | None | 30685.0 | 29803.0 | 29877.0 | 194971.0 | 391653.0 | 181687.0 | 163976.0 | 161744.0 | 151549.0 | None | None | None | None | None | None | None | None | None | None | None | None | None | None | None | None | None | None | None | None | None | None | None | None | None | None | None |
| HFHSD+L | 56029.82133333335 | 24693.88888888889 | 111178.0 | 128182.1 | 135995.8 | 92313.44 | 84648.56 | 81671.91 | 12016.86 | 2609.16 | 7055.77 | 40519.69 | 23627.18 | 44240.5 | 40445.27 | 9748.54 | 26194.54 | None | None | None | None | None | None | None | None | None | None | None | None | None | None | None | None | None | None | None | None | None | 33331.0 | 28517.0 | 27304.0 | 16334.0 | 15144.0 | 16223.0 | 24557.0 | 13916.0 | 46919.0 | None | None | None | None | None | None | None | None | None | None | None | None | None | None | None | None | None | None | None | None | None | None | None | None | None | None | None |TNF-α (ng/μg of proteins)
Surface area (μm2)
***
*
**
*
***
***
*
### Chart
| Category | Mean Males | Mean Females | Males1 | Males2 | Males3 | Males4 | Males5 | Males6 | Males7 | Males8 | Females1 | Females2 | Females3 | Females4 | Females5 | Females6 | Females7 | Females8 |
|---|---|---|---|---|---|---|---|---|---|---|---|---|---|---|---|---|---|---|
| SD | 4.567142857142857 | 5.181666666666667 | 6.82 | 4.02 | 4.61 | 3.95 | 4.61 | 2.83 | 5.13 | None | 2.64 | 5.25 | 6.36 | 6.86 | 3.39 | 6.59 | None | None |
| HFHSD | 7.253333333333334 | 3.71375 | 14.69 | 8.15 | 6.12 | 2.99 | 6.4 | 5.17 | None | None | 6.12 | 3.56 | 6.19 | 3.12 | 3.58 | 2.46 | 2.75 | 1.93 |
| HFHSD+M | 8.15375 | 3.7414285714285715 | 7.46 | 8.75 | 14.33 | 8.85 | 9.85 | 3.86 | 6.89 | 5.24 | 2.04 | 4.58 | 3.15 | 4.0 | 6.76 | 3.68 | 1.98 | None |
| HFHSD+L | 5.66375 | 3.08 | 6.58 | 5.89 | 5.85 | 5.35 | 6.45 | 4.59 | 4.95 | 5.65 | 2.42 | 2.05 | 1.36 | 5.93 | 3.64 | None | None | None |Sex: F(1,54)=17.08, p<0.001
Intervention: NS
Interaction: F(3,54)=3.36, p=0.026
Sex: NS
Intervention: F(3,88)=6.65, p<0.001
Interaction: F(3,88)=2.87, p=0.041
Sex: F(1,165)=4.18, p=0.042
Intervention: F(3,165)=15.41, p<0.001
Interaction: F(3,165)=6.45, p<0.001
Sex: F(1,227)=11.33, p=0.001
Intervention: F(3,227)=7.72, p<0.001
Interaction: NS
GFAP
### Chart
| Category | MALES-AVERAGE | FEMALES-AVERAGE | M1 | M2 | M3 | M4 | M5 | M6 | M7 | M8 | M9 | M10 | M11 | M12 | M13 | M14 | M15 | M16 | M17 | M18 | M19 | M20 | M21 | M22 | M23 | M24 | M25 | M26 | M27 | M28 | M29 | M30 | M31 | M32 | M33 | M34 | M35 | M36 | F1 | F2 | F3 | F4 | F5 | F6 | F7 | F8 | F9 | F10 | F11 | F12 | F13 | F14 | F15 | F16 | F17 | F18 | F19 | F20 | F21 | F22 | F23 | F24 | F25 | F26 | F27 | F28 | F29 | F30 | F31 | F32 | F33 | F34 | F35 | F36 |
|---|---|---|---|---|---|---|---|---|---|---|---|---|---|---|---|---|---|---|---|---|---|---|---|---|---|---|---|---|---|---|---|---|---|---|---|---|---|---|---|---|---|---|---|---|---|---|---|---|---|---|---|---|---|---|---|---|---|---|---|---|---|---|---|---|---|---|---|---|---|---|---|---|---|---|
| SD | 35396.666666666664 | 37631.6875 | 40072.0 | 42047.0 | 28748.0 | 33329.0 | 21583.0 | 56628.0 | 36889.0 | 30923.0 | 28351.0 | None | None | None | None | None | None | None | None | None | None | None | None | None | None | None | None | None | None | None | None | None | None | None | None | None | None | None | 29711.0 | 35181.0 | 21192.0 | 47822.0 | 31002.0 | 31901.0 | 24190.0 | 49070.0 | 28319.0 | 45710.0 | 59186.0 | 49611.0 | 42203.0 | 25788.0 | 39951.0 | 41270.0 | None | None | None | None | None | None | None | None | None | None | None | None | None | None | None | None | None | None | None | None |
| HFHSD | 29951.75 | 22080.727272727272 | 37343.0 | 45665.0 | 39451.0 | 18932.0 | 17739.0 | 36434.0 | 15426.0 | 28624.0 | None | None | None | None | None | None | None | None | None | None | None | None | None | None | None | None | None | None | None | None | None | None | None | None | None | None | None | None | 25734.0 | 13482.0 | 21302.0 | 21552.0 | 20570.0 | 22437.0 | 23236.0 | 20880.0 | 18999.0 | 11654.0 | 32936.0 | 35158.0 | 24106.0 | 23921.0 | 35061.0 | 14620.0 | 24801.0 | 28450.0 | 14759.0 | 17641.0 | 22697.0 | 11780.0 | None | None | None | None | None | None | None | None | None | None | None | None | None | None |
| HFHSD+M | 27305.75 | 25969.0 | 27840.0 | 28598.0 | 32110.0 | 34346.0 | 16455.0 | 35918.0 | 17603.0 | 25576.0 | None | None | None | None | None | None | None | None | None | None | None | None | None | None | None | None | None | None | None | None | None | None | None | None | None | None | None | None | 17740.0 | 17782.0 | 16675.0 | 25538.0 | 23171.0 | 26831.0 | 40114.0 | 32172.0 | 31544.0 | 26264.0 | 27828.0 | None | None | None | None | None | None | None | None | None | None | None | None | None | None | None | None | None | None | None | None | None | None | None | None | None |
| HFHSD+L | 23087.6 | 32540.8 | 22761.0 | 10986.0 | 15813.0 | 19952.0 | 46166.0 | 13281.0 | 28800.0 | 18273.0 | 28362.0 | 26482.0 | None | None | None | None | None | None | None | None | None | None | None | None | None | None | None | None | None | None | None | None | None | None | None | None | None | None | 40386.0 | 38574.0 | 31670.0 | 32072.0 | 20002.0 | None | None | None | None | None | None | None | None | None | None | None | None | None | None | None | None | None | None | None | None | None | None | None | None | None | None | None | None | None | None | None |
### Chart
| Category | MALES-AVERAGE | FEMALES-AVERAGE | M1 | M2 | M3 | M4 | M5 | M6 | M7 | M8 | M9 | M10 | M11 | M12 | M13 | M14 | M15 | M16 | M17 | M18 | M19 | M20 | M21 | M22 | M23 | M24 | M25 | M26 | M27 | M28 | M29 | M30 | M31 | M32 | M33 | M34 | M35 | M36 | F1 | F2 | F3 | F4 | F5 | F6 | F7 | F8 | F9 | F10 | F11 | F12 | F13 | F14 | F15 | F16 | F17 | F18 | F19 | F20 | F21 | F22 | F23 | F24 | F25 | F26 | F27 | F28 | F29 | F30 | F31 | F32 | F33 | F34 | F35 | F36 |
|---|---|---|---|---|---|---|---|---|---|---|---|---|---|---|---|---|---|---|---|---|---|---|---|---|---|---|---|---|---|---|---|---|---|---|---|---|---|---|---|---|---|---|---|---|---|---|---|---|---|---|---|---|---|---|---|---|---|---|---|---|---|---|---|---|---|---|---|---|---|---|---|---|---|---|
| SD | 16606.47619047619 | 17826.322580645163 | 8360.0 | 24159.0 | 17221.0 | 8951.0 | 20141.0 | 10512.0 | 15127.0 | 19452.0 | 16759.0 | 14324.0 | 14342.0 | 11123.0 | 20872.0 | 12224.0 | 13059.0 | 15216.0 | 15223.0 | 23552.0 | 11809.0 | 35778.0 | 20532.0 | None | None | None | None | None | None | None | None | None | None | None | None | None | None | None | 22941.0 | 26859.0 | 28176.0 | 35803.0 | 16608.0 | 11505.0 | 23913.0 | 26215.0 | 17259.0 | 13197.0 | 24548.0 | 22739.0 | 16037.0 | 15293.0 | 13774.0 | 12120.0 | 10454.0 | 10012.0 | 9699.0 | 13331.0 | 17111.0 | 18003.0 | 24501.0 | 11326.0 | 15489.0 | 13691.0 | 19847.0 | 22207.0 | 11970.0 | 19047.0 | 8941.0 | None | None | None | None | None |
| HFHSD | 12197.266666666666 | 17211.555555555555 | 8901.0 | 10303.0 | 5834.0 | 11973.0 | 18655.0 | 5657.0 | 15104.0 | 14447.0 | 7696.0 | 20242.0 | 16660.0 | 17353.0 | 13750.0 | 18959.0 | 16186.0 | 14503.0 | 10780.0 | 15654.0 | 9824.0 | 11194.0 | 9501.0 | 12436.0 | 8532.0 | 10950.0 | 9861.0 | 7344.0 | 11966.0 | 11439.0 | 11729.0 | 8485.0 | None | None | None | None | None | None | 12340.0 | 10520.0 | 13261.0 | 16260.0 | 13479.0 | 18706.0 | 7462.0 | 7501.0 | 9163.0 | 8704.0 | 13229.0 | 9869.0 | 19080.0 | 11019.0 | 15874.0 | 9976.0 | 8503.0 | 16399.0 | 26198.0 | 21345.0 | 19733.0 | 13538.0 | 12877.0 | 22122.0 | 32957.0 | 21013.0 | 23660.0 | 20058.0 | 26079.0 | 19697.0 | 30991.0 | 20416.0 | 18655.0 | 23618.0 | 21254.0 | 24060.0 |
| HFHSD+M | 16594.714285714286 | 21146.821428571428 | 18829.0 | 10652.0 | 12771.0 | 19586.0 | 26626.0 | 19002.0 | 19522.0 | 17027.0 | 14232.0 | 22680.0 | 18312.0 | 12766.0 | 21580.0 | 15522.0 | 12619.0 | 13295.0 | 18888.0 | 13985.0 | 14277.0 | 14174.0 | 12144.0 | None | None | None | None | None | None | None | None | None | None | None | None | None | None | None | 15003.0 | 14601.0 | 21871.0 | 11846.0 | 21258.0 | 31179.0 | 16949.0 | 27026.0 | 10506.0 | 19444.0 | 26943.0 | 16420.0 | 20788.0 | 29915.0 | 11797.0 | 18728.0 | 20689.0 | 25706.0 | 11681.0 | 14121.0 | 36776.0 | 24054.0 | 18581.0 | 19438.0 | 22962.0 | 23788.0 | 29520.0 | 30521.0 | None | None | None | None | None | None | None | None |
| HFHSD+L | 13110.033333333333 | 13927.258064516129 | 6438.0 | 11375.0 | 14570.0 | 6111.0 | 10170.0 | 9302.0 | 5417.0 | 12792.0 | 9465.0 | 12287.0 | 10232.0 | 7333.0 | 15465.0 | 4271.0 | 26423.0 | 20667.0 | 8101.0 | 9633.0 | 11059.0 | 11419.0 | 9569.0 | 7292.0 | 6861.0 | 22721.0 | 22201.0 | 11438.0 | 23207.0 | 22874.0 | 26626.0 | 17982.0 | None | None | None | None | None | None | 11089.0 | 12762.0 | 9514.0 | 6481.0 | 5338.0 | 3079.0 | 6611.0 | 4382.0 | 4979.0 | 5340.0 | 7994.0 | 12574.0 | 10293.0 | 9538.0 | 16168.0 | 14131.0 | 12995.0 | 13967.0 | 6286.0 | 11076.0 | 7568.0 | 13214.0 | 29283.0 | 15374.0 | 23374.0 | 27495.0 | 25042.0 | 25513.0 | 20848.0 | 34081.0 | 25356.0 | None | None | None | None | None |
### Chart
| Category | MALES-AVERAGE | FEMALES-AVERAGE | M1 | M2 | M3 | M4 | M5 | M6 | M7 | M8 | M9 | M10 | M11 | M12 | M13 | M14 | M15 | M16 | M17 | M18 | M19 | M20 | M21 | M22 | M23 | M24 | M25 | M26 | M27 | M28 | M29 | M30 | M31 | M32 | M33 | M34 | M35 | M36 | F1 | F2 | F3 | F4 | F5 | F6 | F7 | F8 | F9 | F10 | F11 | F12 | F13 | F14 | F15 | F16 | F17 | F18 | F19 | F20 | F21 | F22 | F23 | F24 | F25 | F26 | F27 | F28 | F29 | F30 | F31 | F32 | F33 | F34 | F35 | F36 |
|---|---|---|---|---|---|---|---|---|---|---|---|---|---|---|---|---|---|---|---|---|---|---|---|---|---|---|---|---|---|---|---|---|---|---|---|---|---|---|---|---|---|---|---|---|---|---|---|---|---|---|---|---|---|---|---|---|---|---|---|---|---|---|---|---|---|---|---|---|---|---|---|---|---|---|
| SD | 26141.714285714286 | 23470.809523809523 | 30442.0 | 29018.0 | 30511.0 | 31837.0 | 29350.0 | 46268.0 | 39240.0 | 24040.0 | 37317.0 | 32908.0 | 16213.0 | 5066.0 | 7336.0 | 6438.0 | None | None | None | None | None | None | None | None | None | None | None | None | None | None | None | None | None | None | None | None | None | None | 29798.0 | 10096.0 | 20258.0 | 24540.0 | 19909.0 | 11734.0 | 10756.0 | 20782.0 | 10138.0 | 11680.0 | 20428.0 | 36688.0 | 22337.0 | 29321.0 | 32433.0 | 15308.0 | 42544.0 | 26769.0 | 15516.0 | 37412.0 | 44440.0 | None | None | None | None | None | None | None | None | None | None | None | None | None | None | None |
| HFHSD | 18695.264705882353 | 16847.59259259259 | 30450.0 | 15047.0 | 10715.0 | 11175.0 | 17380.0 | 27593.0 | 24384.0 | 17810.0 | 16839.0 | 18624.0 | 10289.0 | 20175.0 | 16667.0 | 18757.0 | 36132.0 | 12936.0 | 13996.0 | 31288.0 | 17834.0 | 11514.0 | 22176.0 | 16224.0 | 23243.0 | 19891.0 | 16860.0 | 21690.0 | 23516.0 | 14317.0 | 16458.0 | 10377.0 | 24055.0 | 12358.0 | 17726.0 | 17143.0 | None | None | 11465.0 | 22682.0 | 8629.0 | 7500.0 | 9173.0 | 5701.0 | 22235.0 | 6051.0 | 13267.0 | 35315.0 | 22261.0 | 9925.0 | 22601.0 | 22881.0 | 27914.0 | 26814.0 | 12899.0 | 15218.0 | 23474.0 | 17195.0 | 7809.0 | 25329.0 | 13672.0 | 15164.0 | 11369.0 | 16418.0 | 21924.0 | None | None | None | None | None | None | None | None | None |
| HFHSD+M | 20610.071428571428 | 34061.09090909091 | 13274.0 | 11325.0 | 19384.0 | 15219.0 | 10243.0 | 13045.0 | 18993.0 | 29293.0 | 22962.0 | 26623.0 | 40369.0 | 18832.0 | 26838.0 | 22141.0 | None | None | None | None | None | None | None | None | None | None | None | None | None | None | None | None | None | None | None | None | None | None | 28762.0 | 33394.0 | 32804.0 | 36650.0 | 61458.0 | 50015.0 | 38246.0 | 39475.0 | 35825.0 | 37905.0 | 40938.0 | 51926.0 | 32588.0 | 42463.0 | 44931.0 | 24904.0 | 25385.0 | 17830.0 | 22104.0 | 18885.0 | 15866.0 | 16990.0 | None | None | None | None | None | None | None | None | None | None | None | None | None | None |
| HFHSD+L | 13639.777777777777 | 16511.0 | 25847.0 | 12391.0 | 18511.0 | 14749.0 | 12648.0 | 7743.0 | 13449.0 | 11428.0 | 13001.0 | 17257.0 | 11870.0 | 12065.0 | 8238.0 | 11214.0 | 10538.0 | 9085.0 | 17368.0 | 18114.0 | None | None | None | None | None | None | None | None | None | None | None | None | None | None | None | None | None | None | 13580.0 | 12579.0 | 12859.0 | 11563.0 | 9218.0 | 7727.0 | 10959.0 | 15199.0 | 16431.0 | 12736.0 | 11428.0 | 12879.0 | 29775.0 | 25566.0 | 29711.0 | 31966.0 | None | None | None | None | None | None | None | None | None | None | None | None | None | None | None | None | None | None | None | None |IL-1 (ng/μg of proteins)
*†††
*
***
††
**
Surface area (μm2)
*††
*
### Chart
| Category | Mean Males | Mean Females | Males1 | Males2 | Males3 | Males4 | Males5 | Males6 | Males7 | Males8 | Females1 | Females2 | Females3 | Females4 | Females5 | Females6 | Females7 | Females8 |
|---|---|---|---|---|---|---|---|---|---|---|---|---|---|---|---|---|---|---|
| SD | 0.64 | 1.5099999999999998 | 1.36 | 0.57 | 0.45 | 0.63 | 0.57 | 0.21 | 0.69 | None | 0.72 | 1.83 | 0.72 | 2.36 | 0.89 | 2.54 | None | None |
| HFHSD | 4.1 | 1.3399999999999999 | 12.27 | 6.16 | 1.65 | 0.21 | 3.36 | 0.95 | None | None | 1.87 | 3.33 | 2.21 | 1.36 | 0.68 | 0.43 | 0.42 | 0.42 |
| HFHSD+M | 5.317500000000001 | 2.7728571428571427 | 3.37 | 15.5 | 7.11 | 4.78 | 6.27 | 0.88 | 4.24 | 0.39 | 0.43 | 1.23 | 3.61 | 4.94 | 6.45 | 2.23 | 0.52 | None |
| HFHSD+L | 2.3575 | 1.098 | 2.61 | 1.45 | 1.73 | 1.31 | 4.07 | 4.89 | 1.41 | 1.39 | 1.75 | 0.6 | 0.27 | 1.54 | 1.33 | None | None | None |Sex: NS
Intervention: F(3,54)=3.43, p=0.024
Interaction: NS
STD
HFHSD
HFHSD+L
HFHSD+M
STD
HFHSD
HFHSD+L
HFHSD+M
STD
HFHSD
HFHSD+L
HFHSD+M
*
IL-6 (ng/μg of proteins)
| Post hoc IGF-1Rβ LH Interaction of sex and intervention effects: STD(m) vs. HFHSD(m) p=0.014; STD (m) vs. HFHSD+M (m) p=0.025; STD (m) vs. HFHSD+L (m) p=0.045; STD (f) vs. HFHSD (f) p=0.027; STD (m) vs. HFHSD+L (f) p=0.002; PVN Interaction of sex and intervention effects: STD (f) vs. HFHSD (f) p<0.001; STD (f) vs. HFHSD+L (f) p<0.001; |
| --- |
| GFAP ARC Interaction of sex and intervention effects: STD (m) vs. HFHSD+L (m) p=0.023; STD (f) vs. HFHSD (f) p<0.001, STD (f) vs. HFHSD+M (f) p=0.008; LH Intervention effect: STD vs. HFHSD+L p=0.011; HFHSD vs. HFHSD+M p=0.003; PVN Interaction of sex and intervention effects: STD (m) vs. HFHSD+L (m) p=0.015; HFHSD (m) vs. HFHSD+L (m) p=0.008; STD (f) vs. HFHSD (f) p=0.021; HFHSD (f) vs. HFHSD+M (f) p<0.001; |
STD
HFHSD
HFHSD+L
HFHSD+M
Extended Data figure 4.1. Sex, intervention, and their interaction affect gliosis in hypothalamic nuclei, while only intervention affects inflammation markers in subcutaneous adipose tissue.
A) Interaction plots of sex and intervention effects on the expression level of insulin-like growth factor 1 receptor β-subunit (IGF-1Rβ), and glial marker glial fibrillary acidic protein (GFAP) in hypothalamic nuclei: arcuate nucleus (ARC), lateral nucleus of hypothalamus (LH), and paraventricular nucleus (PVN). Two-way ANOVA and Games-Howell post hoc test for between-group comparisons. B) Interaction plots of intervention and sex effects on the TNF-α, IL-1, and IL-6 from the subcutaneous adipose tissue. Two-way ANOVA and Games-Howell post hoc test for between-group comparisons, black symbol – experimental groups including both sexes, green symbol – male groups, red symbol – female groups, *compared to STD, †compared to HFHSD, */†p < 0.05, **/††p < 0.01, ***/†††p < 0.001. Group sample size was as followed: N(STD males)= 7, N(HFHSD males)= 6, N(HFHSD+M males)= 8, N(HFHSD+L males)= 8, N(STD females)= 7, N(HFHSD females)= 8, N(HFHSD+M females)= 7, N(HFHSD+L females)= 5. Abbreviations: NS – not significant, f – female, m – male, STD – standard diet group, HFHSD – high-fat and high-sucrose diet group, HFHSD+M – HFHSD treated with metformin, HFHSD+L – HFHSD treated with liraglutide.

## Slide 2
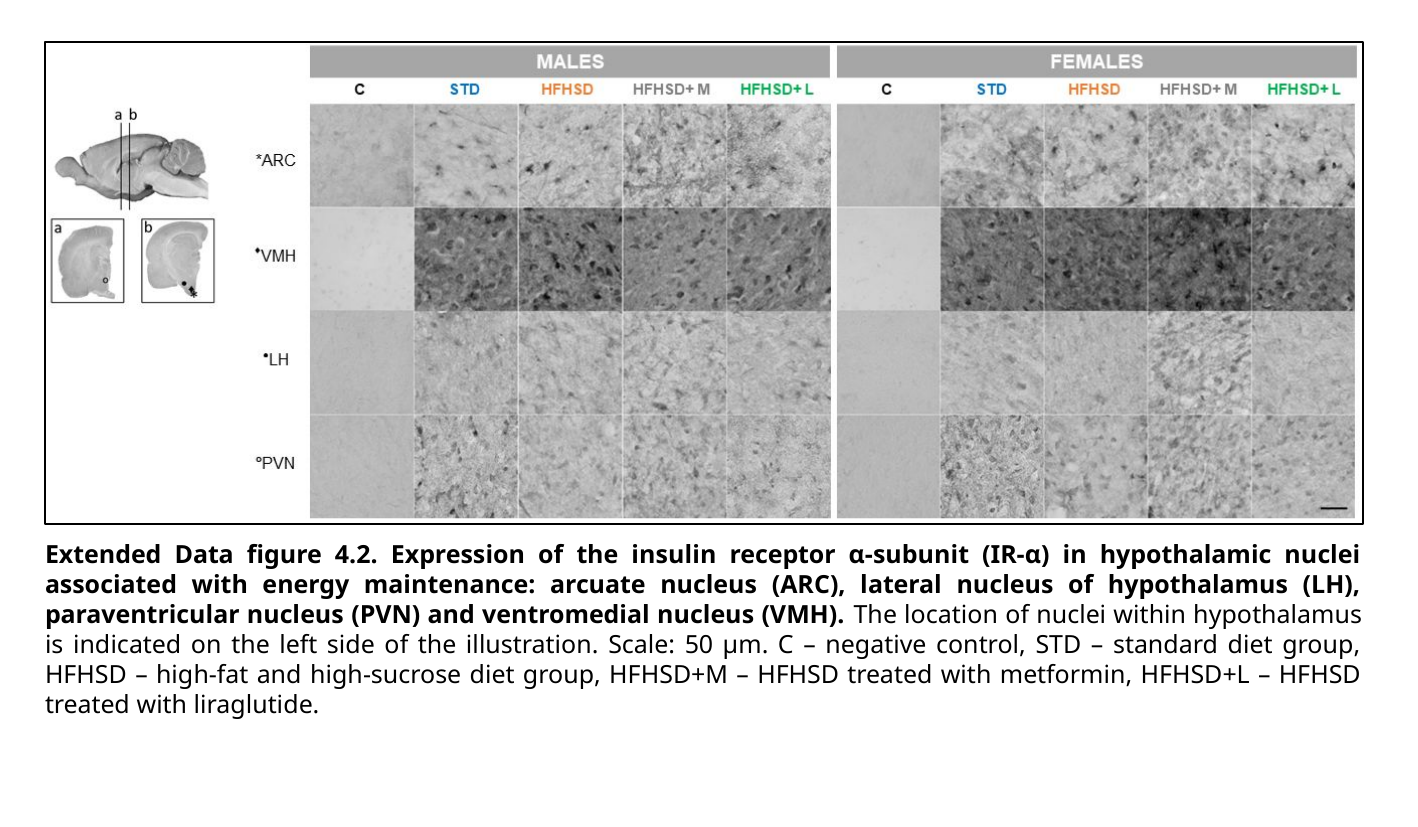

Extended Data figure 4.2. Expression of the insulin receptor α-subunit (IR-α) in hypothalamic nuclei associated with energy maintenance: arcuate nucleus (ARC), lateral nucleus of hypothalamus (LH), paraventricular nucleus (PVN) and ventromedial nucleus (VMH). The location of nuclei within hypothalamus is indicated on the left side of the illustration. Scale: 50 µm. C – negative control, STD – standard diet group, HFHSD – high-fat and high-sucrose diet group, HFHSD+M – HFHSD treated with metformin, HFHSD+L – HFHSD treated with liraglutide.

## Slide 3
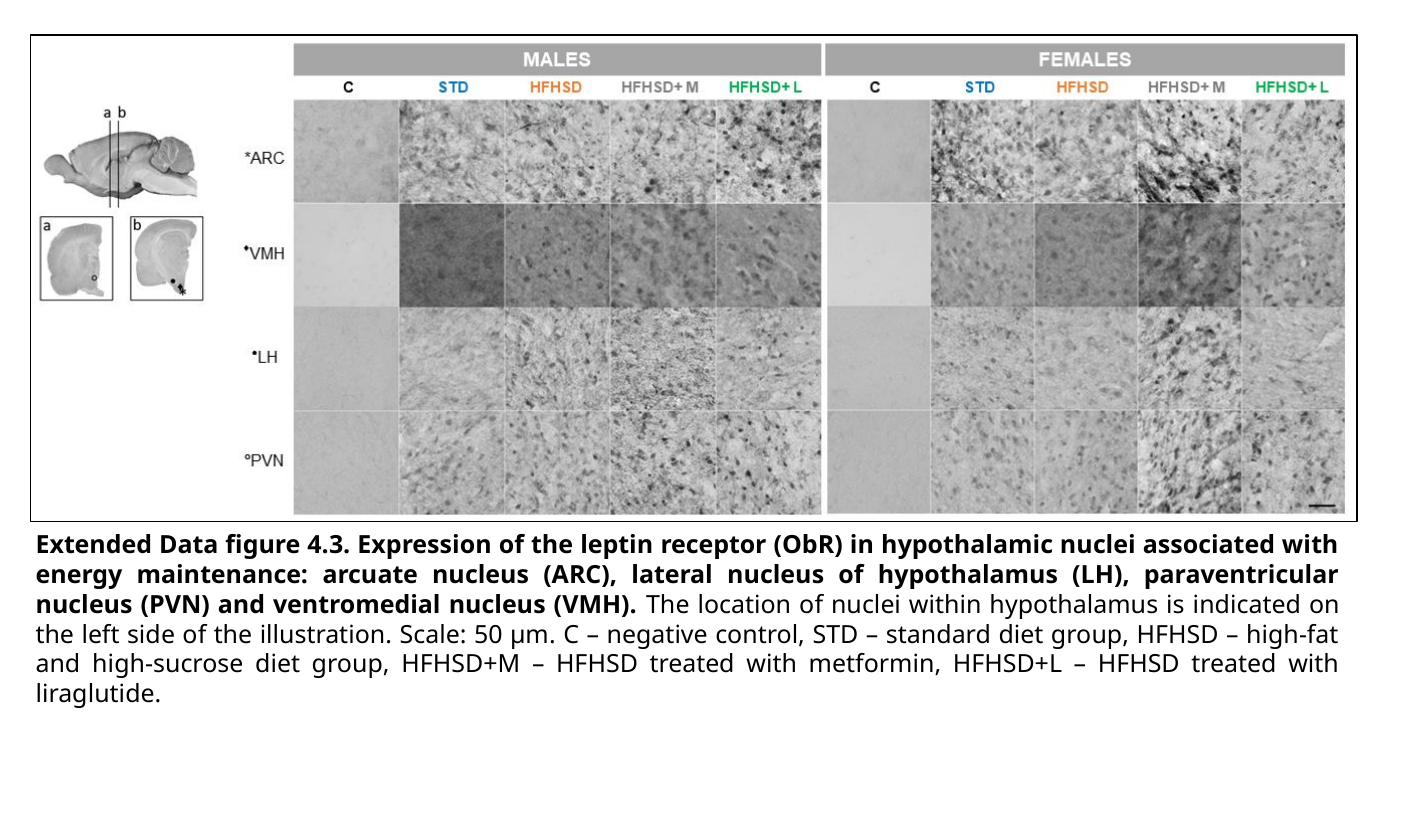

Extended Data figure 4.3. Expression of the leptin receptor (ObR) in hypothalamic nuclei associated with energy maintenance: arcuate nucleus (ARC), lateral nucleus of hypothalamus (LH), paraventricular nucleus (PVN) and ventromedial nucleus (VMH). The location of nuclei within hypothalamus is indicated on the left side of the illustration. Scale: 50 µm. C – negative control, STD – standard diet group, HFHSD – high-fat and high-sucrose diet group, HFHSD+M – HFHSD treated with metformin, HFHSD+L – HFHSD treated with liraglutide.

## Slide 4
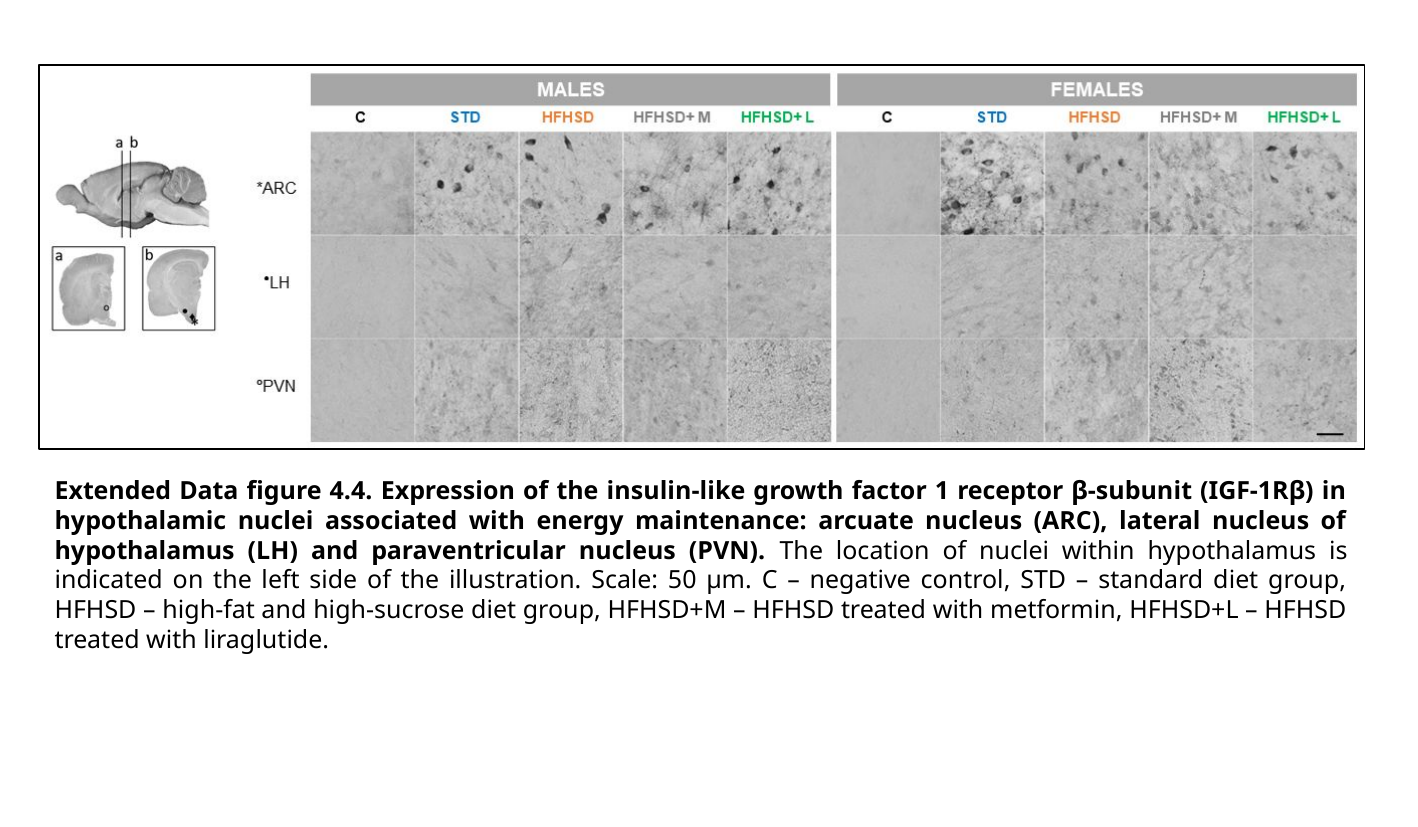

Extended Data figure 4.4. Expression of the insulin-like growth factor 1 receptor β-subunit (IGF-1Rβ) in hypothalamic nuclei associated with energy maintenance: arcuate nucleus (ARC), lateral nucleus of hypothalamus (LH) and paraventricular nucleus (PVN). The location of nuclei within hypothalamus is indicated on the left side of the illustration. Scale: 50 µm. C – negative control, STD – standard diet group, HFHSD – high-fat and high-sucrose diet group, HFHSD+M – HFHSD treated with metformin, HFHSD+L – HFHSD treated with liraglutide.

## Slide 5
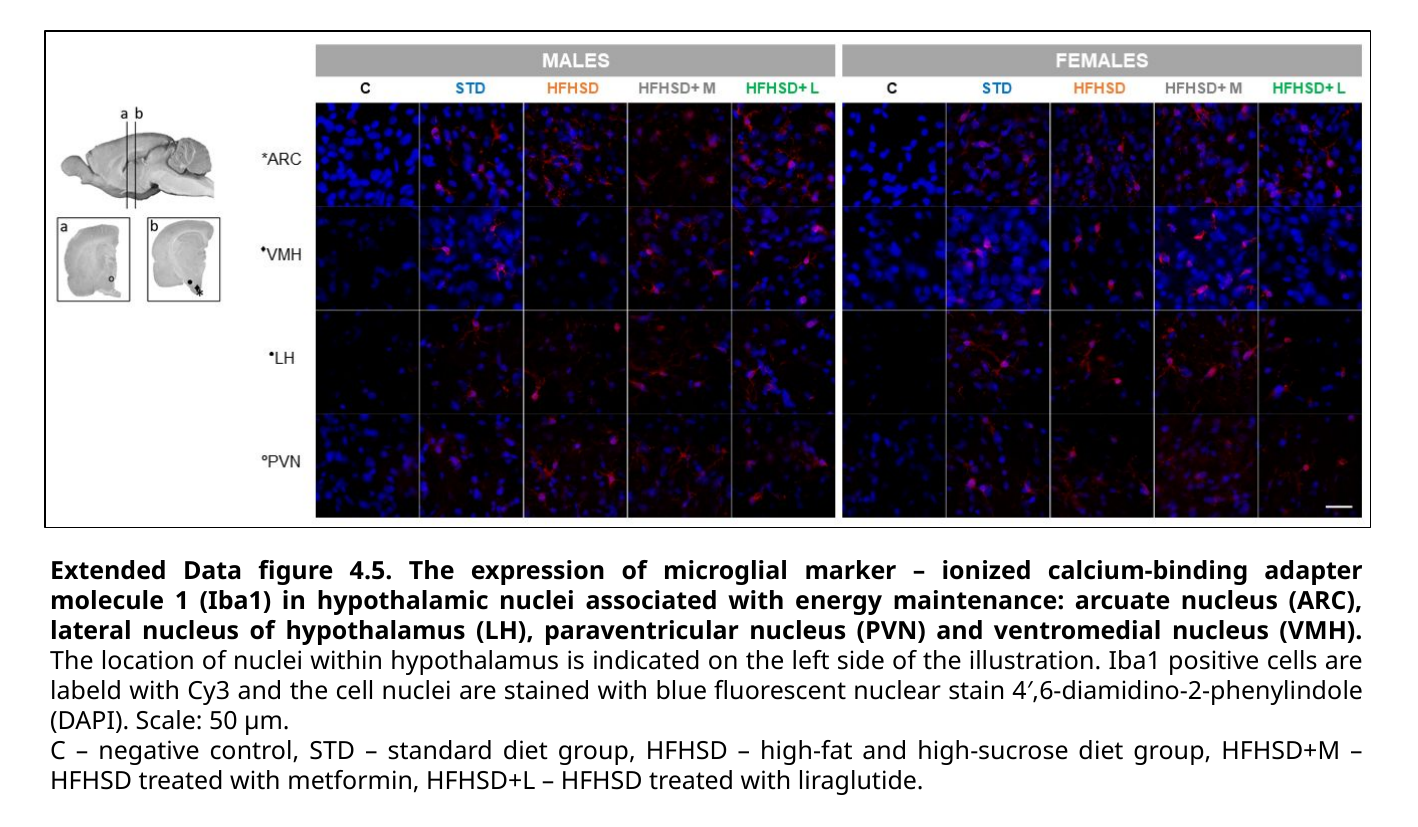

Extended Data figure 4.5. The expression of microglial marker – ionized calcium-binding adapter molecule 1 (Iba1) in hypothalamic nuclei associated with energy maintenance: arcuate nucleus (ARC), lateral nucleus of hypothalamus (LH), paraventricular nucleus (PVN) and ventromedial nucleus (VMH). The location of nuclei within hypothalamus is indicated on the left side of the illustration. Iba1 positive cells are labeld with Cy3 and the cell nuclei are stained with blue fluorescent nuclear stain 4′,6-diamidino-2-phenylindole (DAPI). Scale: 50 µm.
C – negative control, STD – standard diet group, HFHSD – high-fat and high-sucrose diet group, HFHSD+M – HFHSD treated with metformin, HFHSD+L – HFHSD treated with liraglutide.

## Slide 6
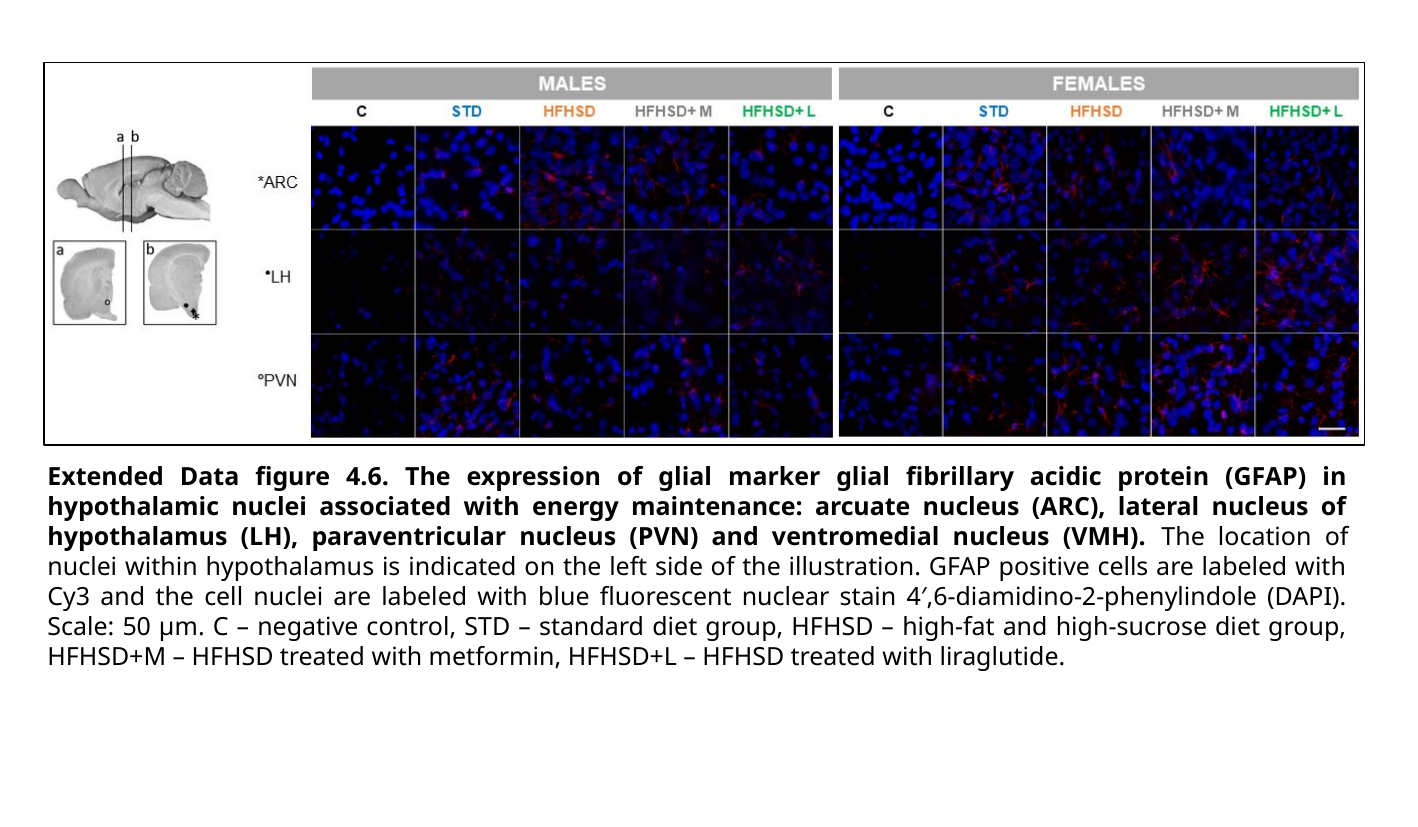

Extended Data figure 4.6. The expression of glial marker glial fibrillary acidic protein (GFAP) in hypothalamic nuclei associated with energy maintenance: arcuate nucleus (ARC), lateral nucleus of hypothalamus (LH), paraventricular nucleus (PVN) and ventromedial nucleus (VMH). The location of nuclei within hypothalamus is indicated on the left side of the illustration. GFAP positive cells are labeled with Cy3 and the cell nuclei are labeled with blue fluorescent nuclear stain 4′,6-diamidino-2-phenylindole (DAPI). Scale: 50 µm. C – negative control, STD – standard diet group, HFHSD – high-fat and high-sucrose diet group, HFHSD+M – HFHSD treated with metformin, HFHSD+L – HFHSD treated with liraglutide.

## Slide 7
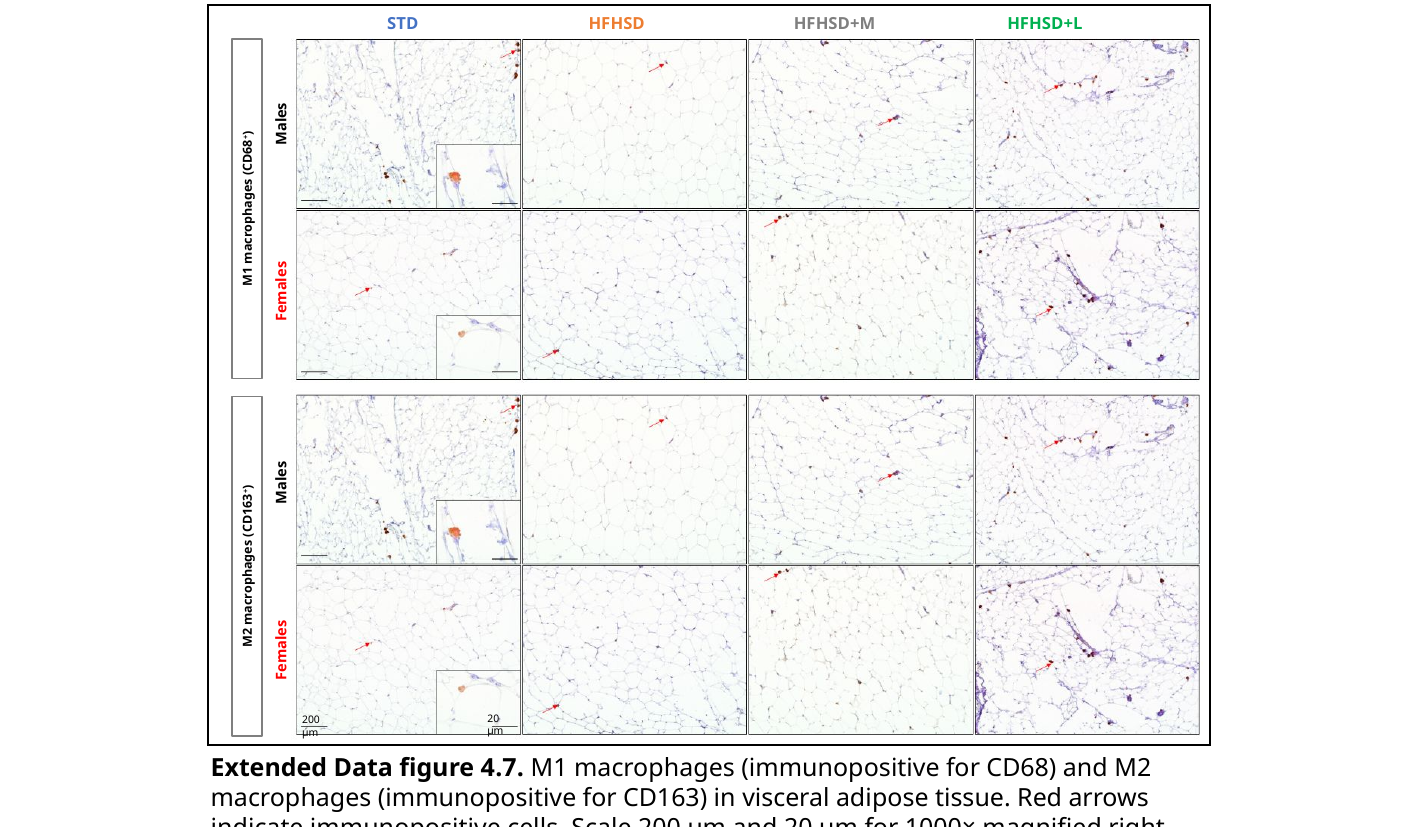

STD HFHSD HFHSD+M HFHSD+L
M1 macrophages (CD68+)
M2 macrophages (CD163+)
Males
Females
Males
Females
20 μm
200 μm
Extended Data figure 4.7. M1 macrophages (immunopositive for CD68) and M2 macrophages (immunopositive for CD163) in visceral adipose tissue. Red arrows indicate immunopositive cells. Scale 200 μm and 20 μm for 1000× magnified right corner images. STD – standard diet group, HFHSD – high-fat and high-sucrose diet group, HFHSD+M – HFHSD treated with metformin, HFHSD+L – HFHSD treated with liraglutide.
