# Supplementary material for: Elderly rats fed with a high-fat high-sucrose diet developed sex-dependent metabolic syndrome regardless of long-term metformin and liraglutide treatment
Source: Front Endocrinol (Lausanne). 2023 Oct 20;14:1181064. doi: 10.3389/fendo.2023.1181064 (PMC10623428; doi:10.3389/fendo.2023.1181064)
Supplement: Supplementary file 1 [file DataSheet_1.zip › Extended Data/Extended Data Fig. 5.PPTX]

## Slide 1
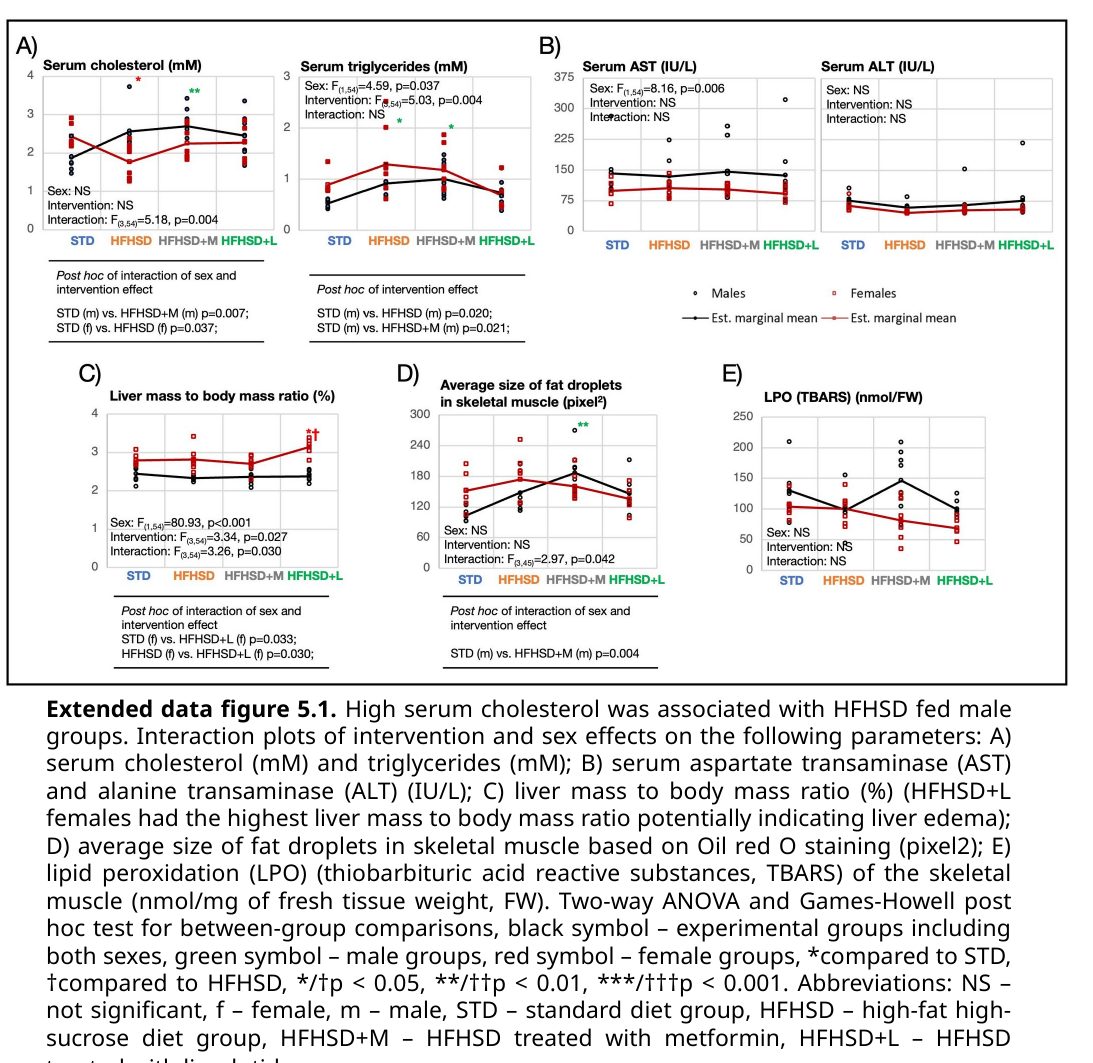

Extended data figure 5.1. High serum cholesterol was associated with HFHSD fed male groups. Interaction plots of intervention and sex effects on the following parameters: A) serum cholesterol (mM) and triglycerides (mM); B) serum aspartate transaminase (AST) and alanine transaminase (ALT) (IU/L); C) liver mass to body mass ratio (%) (HFHSD+L females had the highest liver mass to body mass ratio potentially indicating liver edema); D) average size of fat droplets in skeletal muscle based on Oil red O staining (pixel2); E) lipid peroxidation (LPO) (thiobarbituric acid reactive substances, TBARS) of the skeletal muscle (nmol/mg of fresh tissue weight, FW). Two-way ANOVA and Games-Howell post hoc test for between-group comparisons, black symbol – experimental groups including both sexes, green symbol – male groups, red symbol – female groups, *compared to STD, †compared to HFHSD, */†p < 0.05, **/††p < 0.01, ***/†††p < 0.001. Abbreviations: NS – not significant, f – female, m – male, STD – standard diet group, HFHSD – high-fat high-sucrose diet group, HFHSD+M – HFHSD treated with metformin, HFHSD+L – HFHSD treated with liraglutide.

## Slide 2
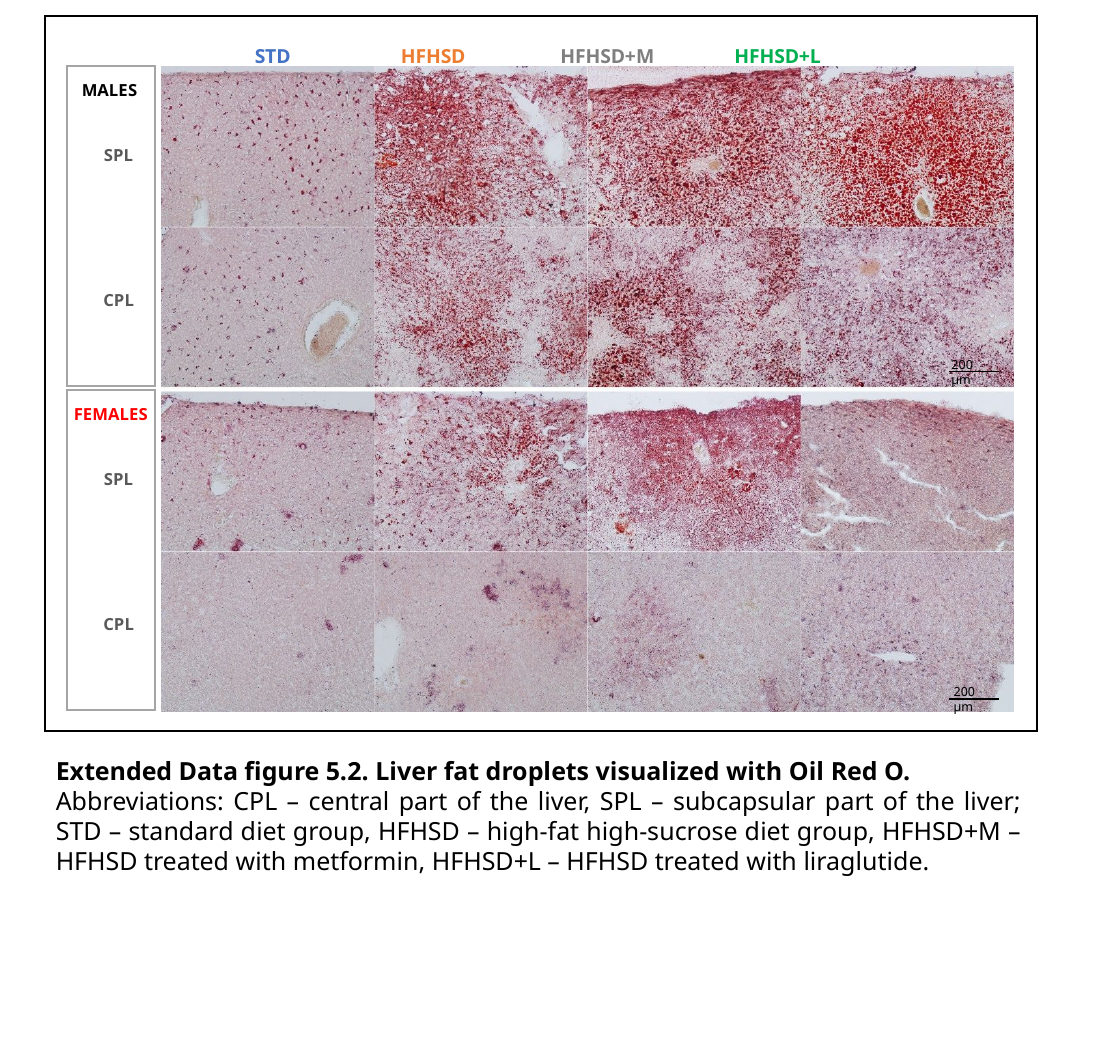

STD HFHSD HFHSD+M HFHSD+L
MALES
SPL
CPL
200 μm
FEMALES
SPL
CPL
200 μm
Extended Data figure 5.2. Liver fat droplets visualized with Oil Red O.
Abbreviations: CPL – central part of the liver, SPL – subcapsular part of the liver; STD – standard diet group, HFHSD – high-fat high-sucrose diet group, HFHSD+M – HFHSD treated with metformin, HFHSD+L – HFHSD treated with liraglutide.

## Slide 3
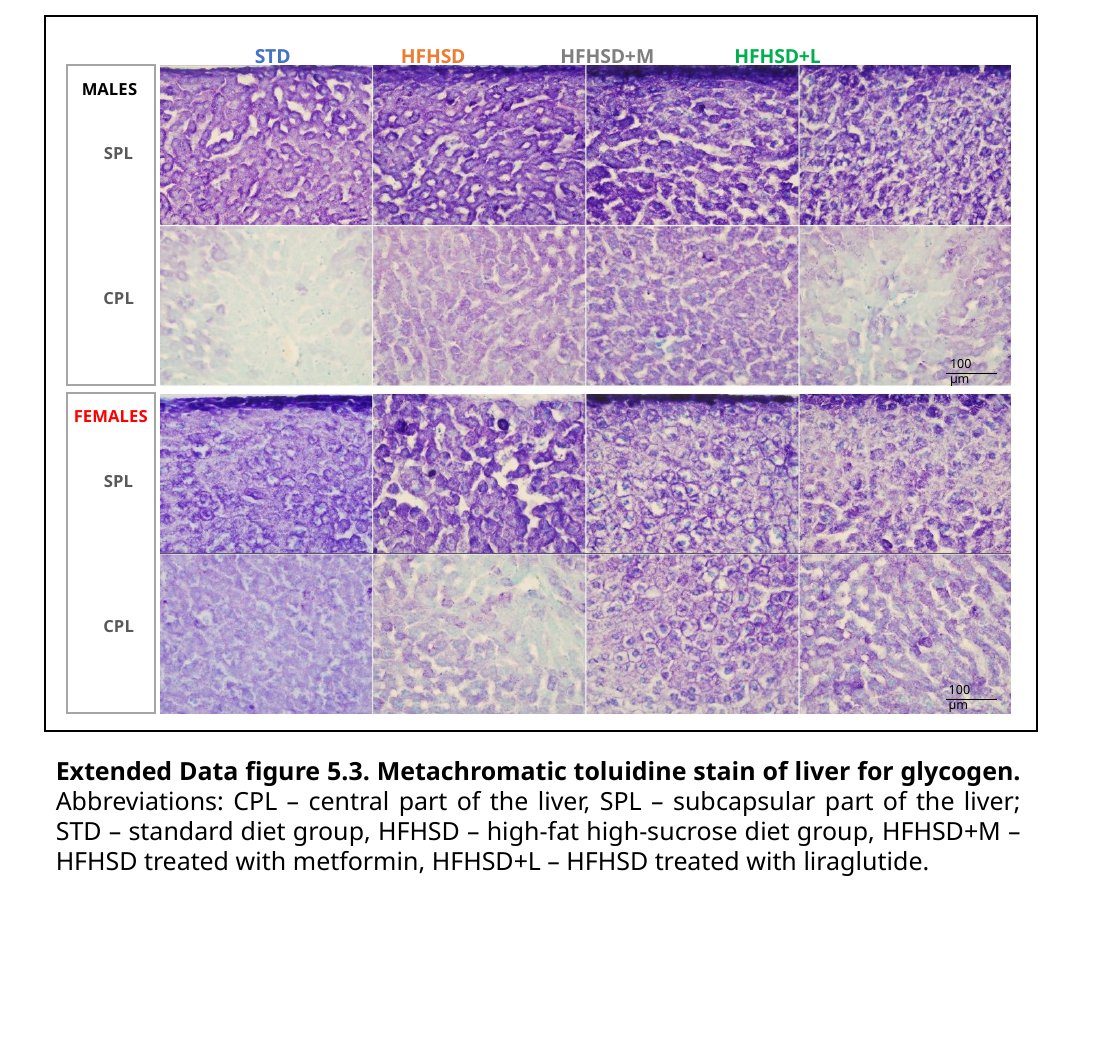

STD HFHSD HFHSD+M HFHSD+L
MALES
SPL
CPL
100 μm
FEMALES
SPL
CPL
100 μm
Extended Data figure 5.3. Metachromatic toluidine stain of liver for glycogen. Abbreviations: CPL – central part of the liver, SPL – subcapsular part of the liver; STD – standard diet group, HFHSD – high-fat high-sucrose diet group, HFHSD+M – HFHSD treated with metformin, HFHSD+L – HFHSD treated with liraglutide.

## Slide 4
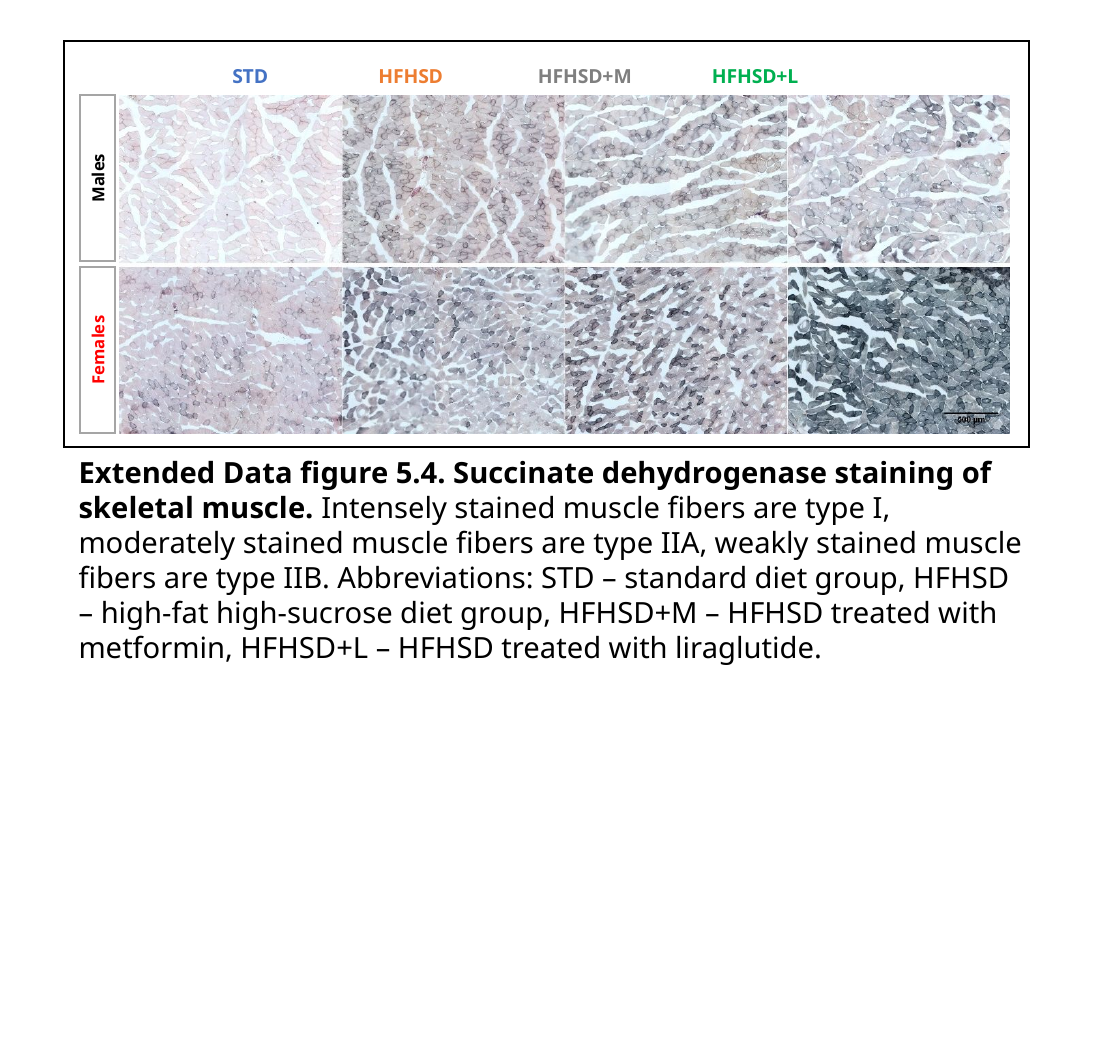

STD HFHSD HFHSD+M HFHSD+L
Males
Females
Extended Data figure 5.4. Succinate dehydrogenase staining of skeletal muscle. Intensely stained muscle fibers are type I, moderately stained muscle fibers are type IIA, weakly stained muscle fibers are type IIB. Abbreviations: STD – standard diet group, HFHSD – high-fat high-sucrose diet group, HFHSD+M – HFHSD treated with metformin, HFHSD+L – HFHSD treated with liraglutide.

## Slide 5
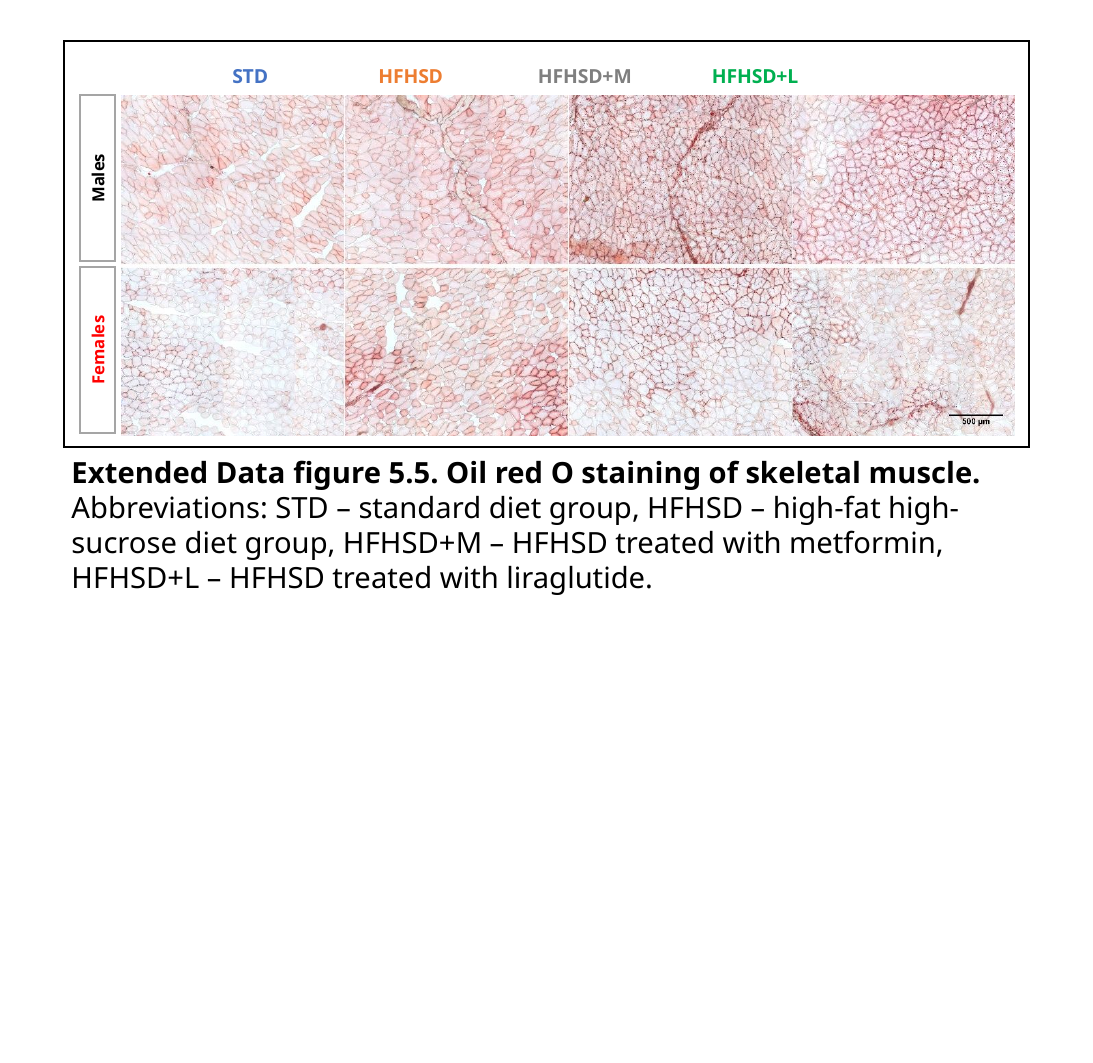

STD HFHSD HFHSD+M HFHSD+L
Males
Females
Extended Data figure 5.5. Oil red O staining of skeletal muscle. Abbreviations: STD – standard diet group, HFHSD – high-fat high-sucrose diet group, HFHSD+M – HFHSD treated with metformin, HFHSD+L – HFHSD treated with liraglutide.
