# Supplementary material for: Elderly rats fed with a high-fat high-sucrose diet developed sex-dependent metabolic syndrome regardless of long-term metformin and liraglutide treatment
Source: Front Endocrinol (Lausanne). 2023 Oct 20;14:1181064. doi: 10.3389/fendo.2023.1181064 (PMC10623428; doi:10.3389/fendo.2023.1181064)
Supplement: Supplementary file 1 [file DataSheet_1.zip › Extended Data/Extended Data Fig. 6.PPTX]

## Slide 1
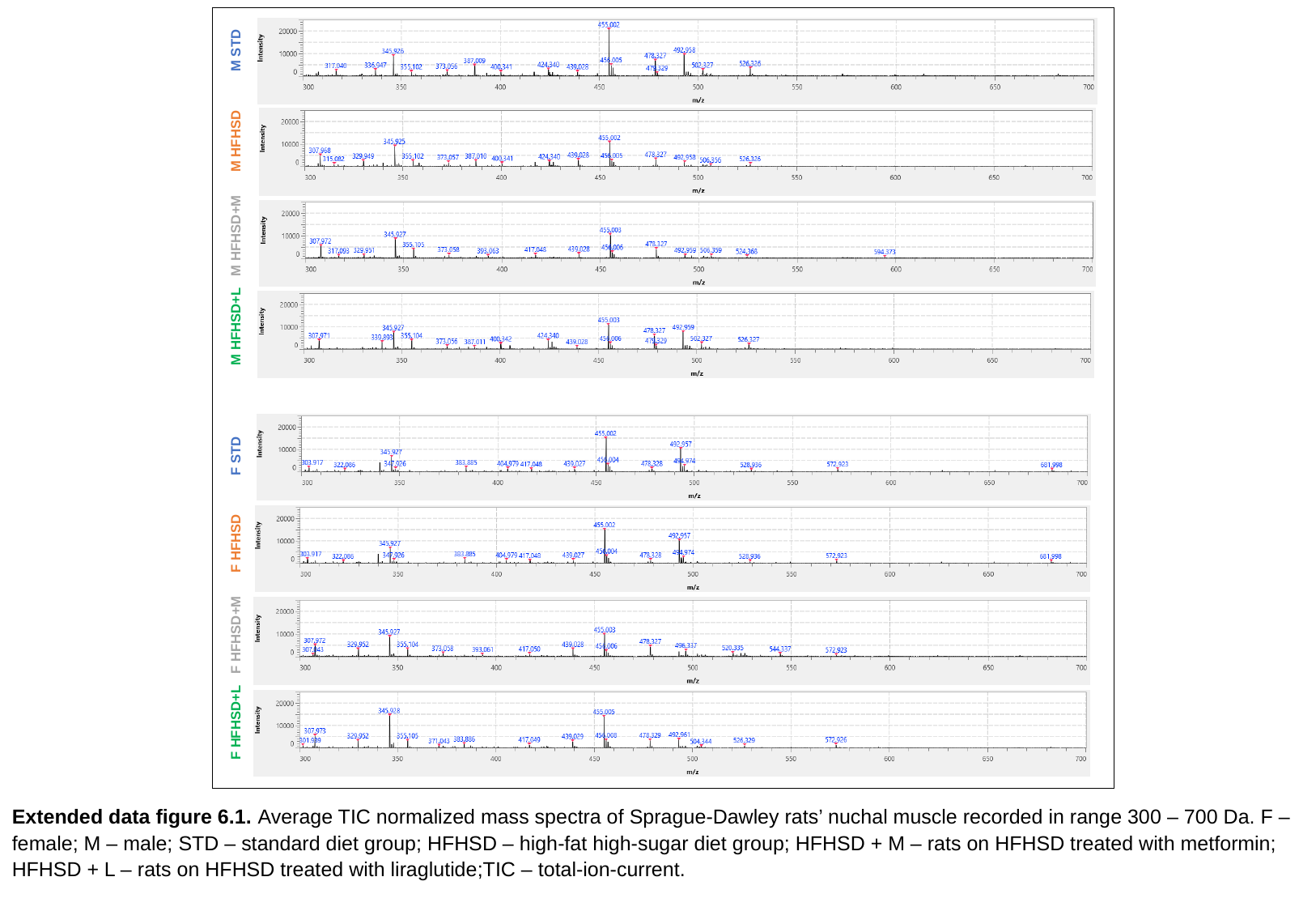

M HFHSD+L M HFHSD+M M HFHSD M STD
F HFHSD+L F HFHSD+M F HFHSD F STD
Extended data figure 6.1. Average TIC normalized mass spectra of Sprague-Dawley rats’ nuchal muscle recorded in range 300 – 700 Da. F – female; M – male; STD – standard diet group; HFHSD – high-fat high-sugar diet group; HFHSD + M – rats on HFHSD treated with metformin; HFHSD + L – rats on HFHSD treated with liraglutide;TIC – total-ion-current.

## Slide 2
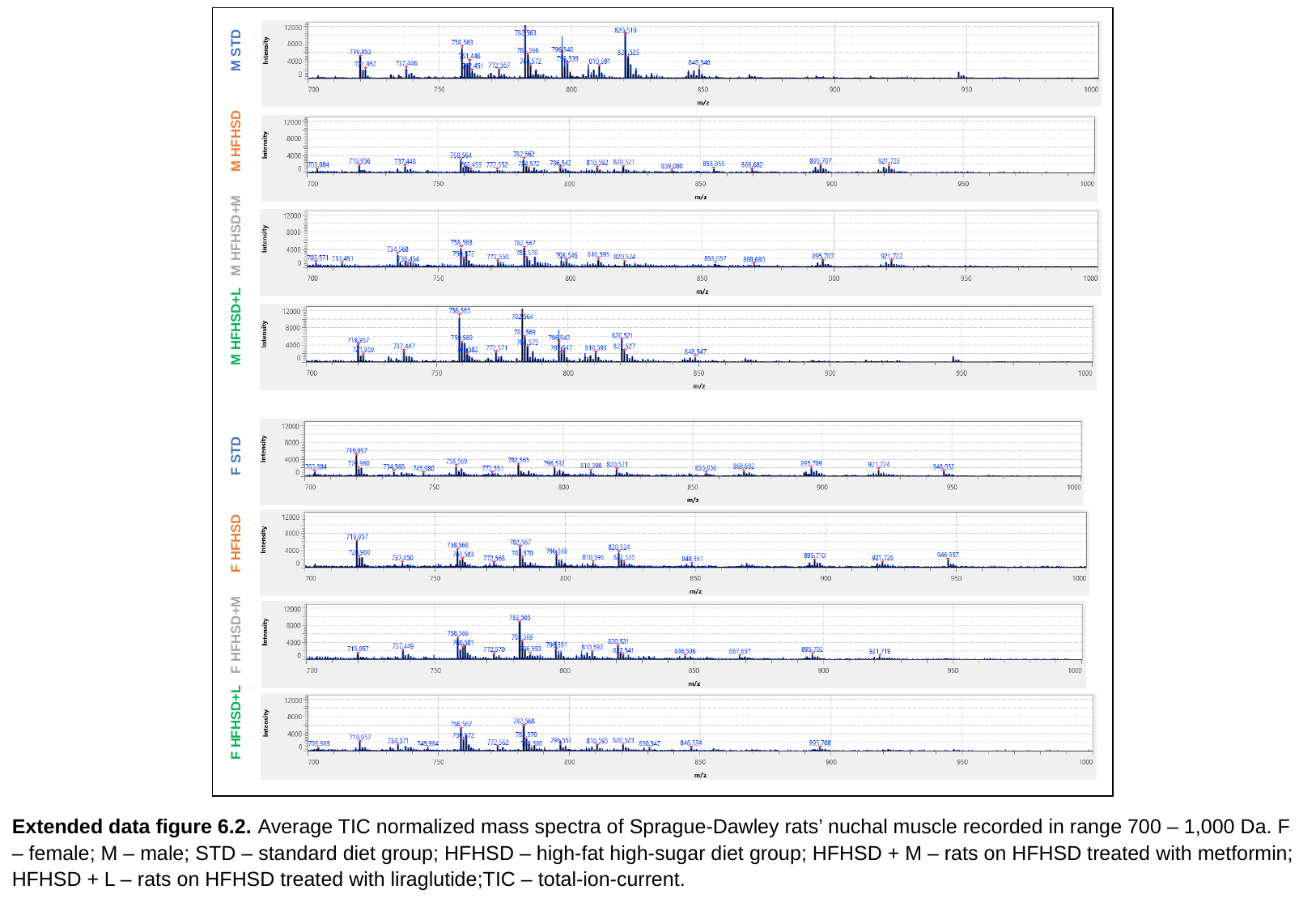

M HFHSD+L M HFHSD+M M HFHSD M STD
F HFHSD+L F HFHSD+M F HFHSD F STD
Extended data figure 6.2. Average TIC normalized mass spectra of Sprague-Dawley rats’ nuchal muscle recorded in range 700 – 1,000 Da. F – female; M – male; STD – standard diet group; HFHSD – high-fat high-sugar diet group; HFHSD + M – rats on HFHSD treated with metformin; HFHSD + L – rats on HFHSD treated with liraglutide;TIC – total-ion-current.

## Slide 3
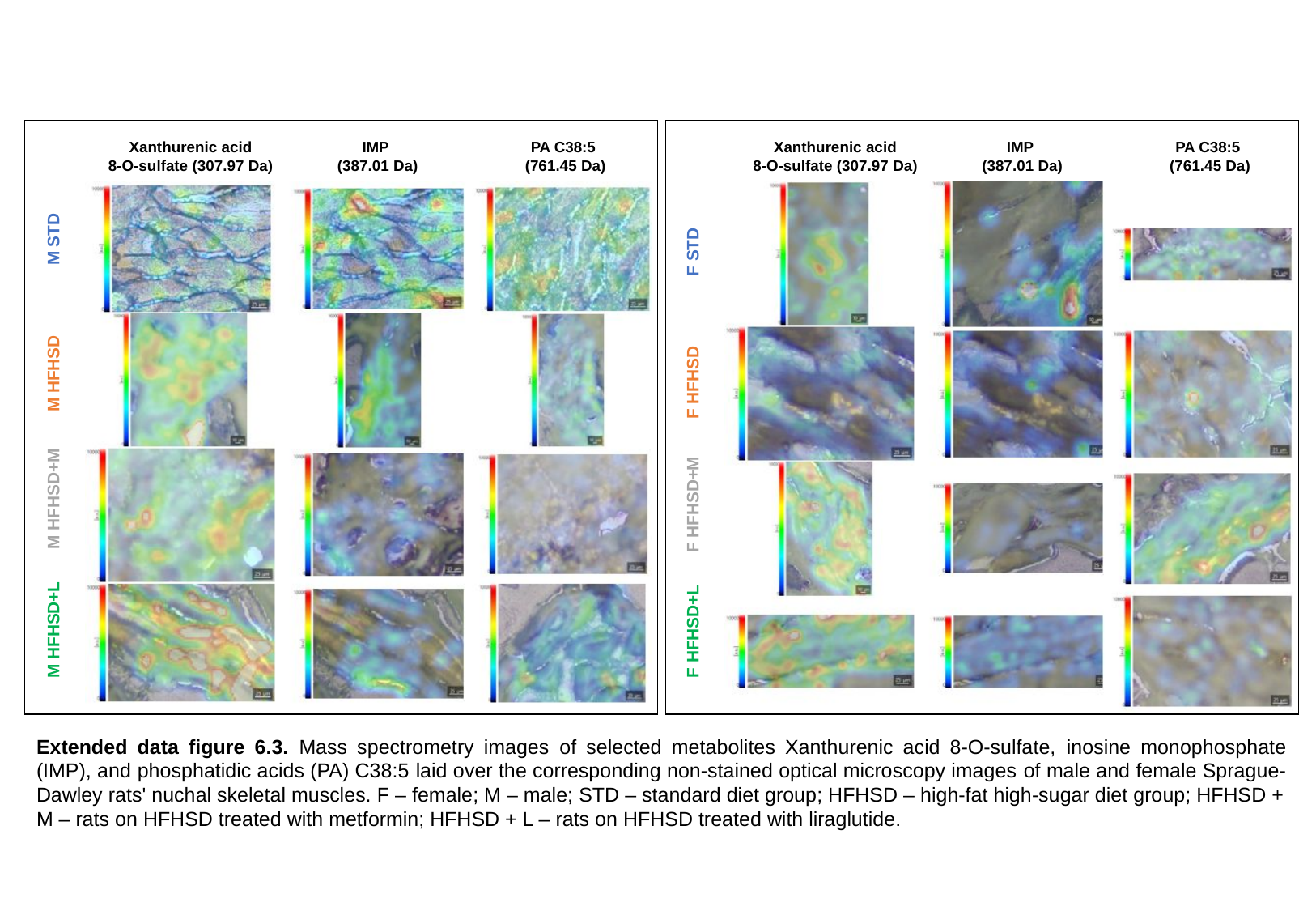

Xanthurenic acid
8-O-sulfate (307.97 Da)
IMP
(387.01 Da)
PA C38:5
(761.45 Da)
Xanthurenic acid
8-O-sulfate (307.97 Da)
IMP
(387.01 Da)
PA C38:5
(761.45 Da)
M HFHSD+L M HFHSD+M M HFHSD M STD
F HFHSD+L F HFHSD+M F HFHSD F STD
Extended data figure 6.3. Mass spectrometry images of selected metabolites Xanthurenic acid 8-O-sulfate, inosine monophosphate (IMP), and phosphatidic acids (PA) C38:5 laid over the corresponding non-stained optical microscopy images of male and female Sprague-Dawley rats' nuchal skeletal muscles. F – female; M – male; STD – standard diet group; HFHSD – high-fat high-sugar diet group; HFHSD + M – rats on HFHSD treated with metformin; HFHSD + L – rats on HFHSD treated with liraglutide.

## Slide 4
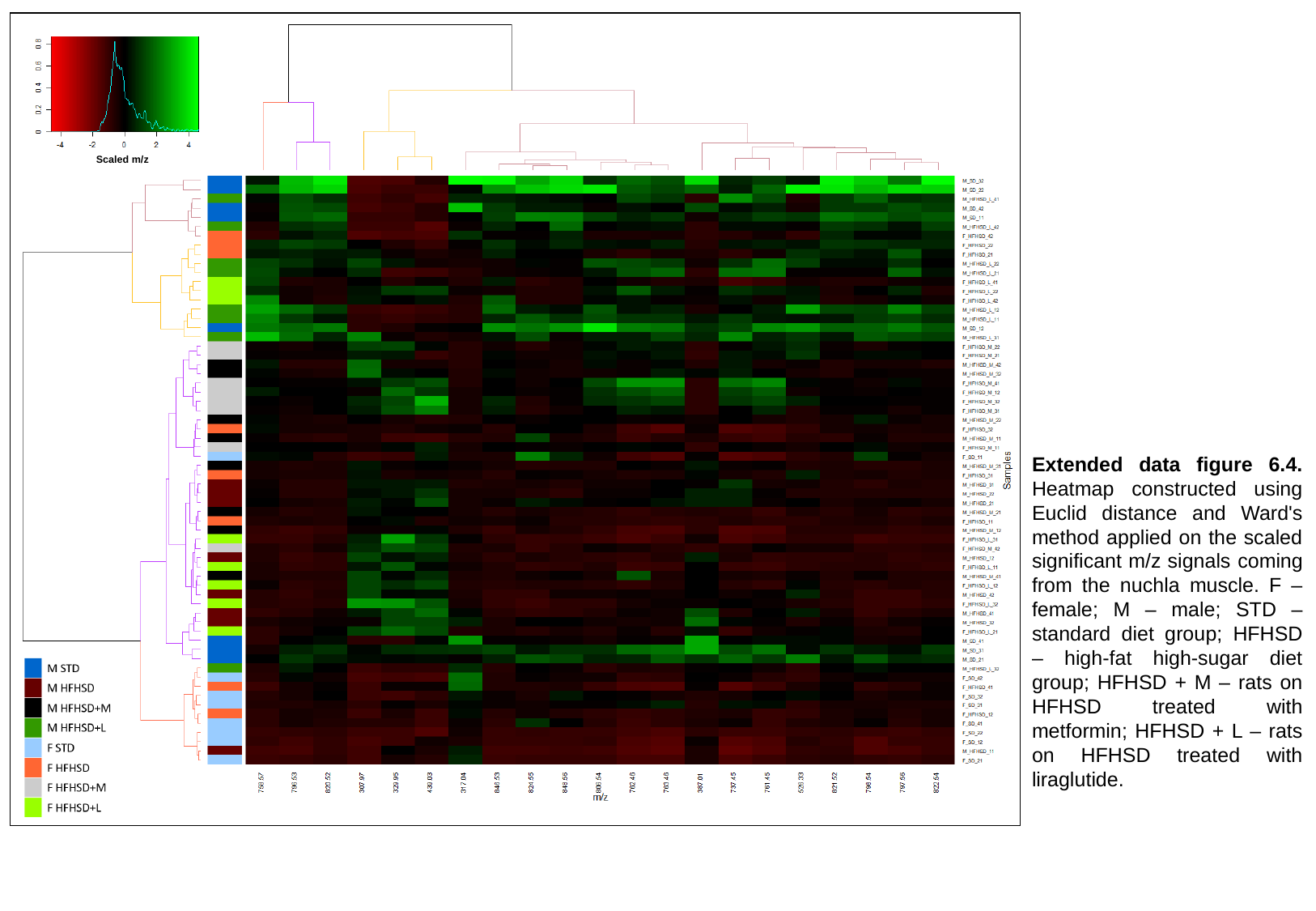

Scaled m/z
Extended data figure 6.4. Heatmap constructed using Euclid distance and Ward's method applied on the scaled significant m/z signals coming from the nuchla muscle. F – female; M – male; STD – standard diet group; HFHSD – high-fat high-sugar diet group; HFHSD + M – rats on HFHSD treated with metformin; HFHSD + L – rats on HFHSD treated with liraglutide.
