# Supplementary material for: Elderly rats fed with a high-fat high-sucrose diet developed sex-dependent metabolic syndrome regardless of long-term metformin and liraglutide treatment
Source: Front Endocrinol (Lausanne). 2023 Oct 20;14:1181064. doi: 10.3389/fendo.2023.1181064 (PMC10623428; doi:10.3389/fendo.2023.1181064)
Supplement: Supplementary file 1 [file DataSheet_1.zip › Extended Data/Extended Data Figure Captions.DOCX]

**Extended Data Figure Legend**

**Extended Data Fig. 1. Schematic presentation of the study**.

Abbreviations: STD – standard diet group, HFHSD – high-fat high-sucrose diet group, HFHSD+M – HFHSD treated with metformin, HFHSD+L – HFHSD treated with liraglutide, W – experimental week, GT – glucose tolerance test, BC – blood collecting, SC – sacrifice, ITT – insulin tolerance test

**Extended Data Fig. 2. High-fat high-sucrose diet and antidiabetic drugs liraglutide and metformin do not influence the body mass in old rats, yet they affect the caloric intake in a sex-specific manner.**

**A)** Body mass measurements per experimental week (mean ± SD). ↓ – introduction of the antidiabetic drugs; **B)** Interaction plots of intervention and sex effects on the percentage of visceral adipocytes in a particular size classes (I-IV): two-way ANOVA and Bonferroni *post hoc* tests for between-group comparisons. **C)** Interaction plots of sex, intervention, and duration of intervention on the ratio of the whole-group caloric intake and the whole-group body mass (kcal/g) before the treatment with metformin and liraglutide (week 1 – 5), during the early treatment (week 6 – 10), and long-term treatment period (week 10 – 18). Three-way ANOVA and Bonferroni *post hoc* test: green symbol – male groups, red symbol – female groups, *compared to STD, ^†^compared to HFHSD; black symbol – experimental groups including both sexes, *^/†^p < 0.05, **^/††^p < 0.01, ***^/†††^p < 0.001. Abbreviations: NS – not significant. f – female, m – male, STD – standard diet group, HFHSD – high-fat and high-sucrose diet group, HFHSD+M – HFHSD treated with metformin, HFHSD+L – HFHSD treated with liraglutide.

**Extended data figure 3.** **High-fat high-sucrose diet increased glucose variability and normoglycemia set point.**

**A)** Area under the curves (AUCs) of blood glucose values measured at glucose tolerance test (GTT) in experimental weeks 0, 5, 12, and 18. **B)** Additional characteristics of model function G(t) based on measurements from glucose tolerance test (GTT) in experimental weeks 0, 5, 12, and 18: coefficient of determination (R^2^), coefficient of oscillation amplitude decline (α), the basic period of function (h) (T), initial speed of blood glucose increase (G'(0)), the blood glucose concentration at which maximal speed of glucose concentration decrease is attained (mg/dL) (G_I_), maximal speed of glucose concentration decrease (G'_I_), the moment at which G'_I_ is attained (h) (t_I_), estimated variance (EV). **C)** Interaction plots of intervention and sex effects on the plasma corticosterone (ng/ml), plasma adiponectin (µg/mL), Insulin Receptor Substrate 1 (IRS-1) phosphorylation in the liver and adipose tissue (arbitrary units, AU). Two-way ANOVA and Games-Howell *post hoc* test for between-group comparisons. **D)** Interaction plot of intervention and sex effect on the area under the curve (AUC) of blood glucose values measured at insulin tolerance test (ITT) in experimental week 18. **E)** Characteristics of model function based on insulin tolerance test (ITT): coefficient of determination (R^2^), fasting blood glucose (mg/dL) (*H(0)*), maximal glucose (mg/dL) (*H_max_*), the moment at which *H_min_* is reached (h) (*t_min_*), 2-h blood glucose at ITT (mg/dL) (*G(2)*), blood glucose setpoint (asymptote) (mg/dL) (*H_0_*), and area under the curve (*AUC*). Two-way ANOVA and Games-Howell *post hoc* test for between-group comparisons. black symbol – experimental groups including both sexes, green symbol – male groups, red symbol – female groups, *compared to STD, ^†^compared to HFHSD, *^/†^p < 0.05, **^/††^p < 0.01, ***^/†††^p < 0.001. Abbreviations: NS – not significant. f – female, m – male, STD – standard diet group, HFHSD – high-fat high-sucrose diet group, HFHSD+M – HFHSD treated with metformin, HFHSD+L – HFHSD treated with liraglutide.

**Extended Data figure 4.1. Sex, intervention, and their interaction affect gliosis in hypothalamic nuclei, while only intervention affects inflammation markers in subcutaneous adipose tissue.**

**A)** Interaction plots of sex and intervention effects on the expression level of insulin-like growth factor 1 receptor β-subunit (IGF-1Rβ), and glial marker glial fibrillary acidic protein (GFAP) in hypothalamic nuclei: arcuate nucleus (ARC), lateral nucleus of hypothalamus (LH), and paraventricular nucleus (PVN). Two-way ANOVA and Games-Howell *post hoc* test for between-group comparisons. **B)** Interaction plots of intervention and sex effects on the TNF-α, IL-1, and IL-6 from the subcutaneous adipose tissue. Two-way ANOVA and Games-Howell *post hoc* test for between-group comparisons, black symbol – experimental groups including both sexes, green symbol – male groups, red symbol – female groups, *compared to STD, ^†^compared to HFHSD, *^/†^p < 0.05, **^/††^p < 0.01, ***^/†††^p < 0.001. Abbreviations: NS – not significant, f – female, m – male, STD – standard diet group, HFHSD – high-fat and high-sucrose diet group, HFHSD+M – HFHSD treated with metformin, HFHSD+L – HFHSD treated with liraglutide.

**Extended Data figure 4.2. Expression of the insulin receptor α-subunit (IR-α) in hypothalamic nuclei associated with energy maintenance: arcuate nucleus (ARC), lateral nucleus of hypothalamus (LH), paraventricular nucleus (PVN) and ventromedial nucleus (VMH).** The location of nuclei within hypothalamus is indicated on the left side of the illustration. Scale: 50 µm. C – negative control, STD – standard diet group, HFHSD – high-fat and high-sucrose diet group, HFHSD+M – HFHSD treated with metformin, HFHSD+L – HFHSD treated with liraglutide.

**Extended Data figure 4.3. Expression of the leptin receptor (ObR) in hypothalamic nuclei associated with energy maintenance: arcuate nucleus (ARC), lateral nucleus of hypothalamus (LH), paraventricular nucleus (PVN) and ventromedial nucleus (VMH).** The location of nuclei within hypothalamus is indicated on the left side of the illustration. Scale: 50 µm. C – negative control, STD – standard diet group, HFHSD – high-fat and high-sucrose diet group, HFHSD+M – HFHSD treated with metformin, HFHSD+L – HFHSD treated with liraglutide.

**Extended Data figure 4.4. Expression of the insulin-like growth factor 1 receptor β-subunit (IGF-1Rβ) in hypothalamic nuclei associated with energy maintenance: arcuate nucleus (ARC), lateral nucleus of hypothalamus (LH) and paraventricular nucleus (PVN).** The location of nuclei within hypothalamus is indicated on the left side of the illustration. Scale: 50 µm. C – negative control, STD – standard diet group, HFHSD – high-fat and high-sucrose diet group, HFHSD+M – HFHSD treated with metformin, HFHSD+L – HFHSD treated with liraglutide.

**Extended Data figure 4.5. The expression of microglial marker – ionized calcium-binding adapter molecule 1 (Iba1) in hypothalamic nuclei associated with energy maintenance: arcuate nucleus (ARC), lateral nucleus of hypothalamus (LH), paraventricular nucleus (PVN) and ventromedial nucleus (VMH).** The location of nuclei within hypothalamus is indicated on the left side of the illustration. Iba1 positive cells are labeld with Cy3 and the cell nuclei are stained with blue fluorescent nuclear stain 4′,6-diamidino-2-phenylindole (DAPI). Scale: 50 µm. C – negative control, STD – standard diet group, HFHSD – high-fat and high-sucrose diet group, HFHSD+M – HFHSD treated with metformin, HFHSD+L – HFHSD treated with liraglutide.

**Extended Data figure 4.6. The expression of glial marker glial fibrillary acidic protein (GFAP) in hypothalamic nuclei associated with energy maintenance: arcuate nucleus (ARC), lateral nucleus of hypothalamus (LH), paraventricular nucleus (PVN) and ventromedial nucleus (VMH).** The location of nuclei within hypothalamus is indicated on the left side of the illustration. GFAP positive cells are labeled with Cy3 and the cell nuclei are labeled with blue fluorescent nuclear stain 4′,6-diamidino-2-phenylindole (DAPI). Scale: 50 µm. C – negative control, STD – standard diet group, HFHSD – high-fat and high-sucrose diet group, HFHSD+M – HFHSD treated with metformin, HFHSD+L – HFHSD treated with liraglutide.

**Extended Data figure 4.7. M1 macrophages (immunopositive for CD68) and M2 macrophages (immunopositive for CD163) in visceral adipose tissue.** Red arrows indicate immunopositive cells. Scale 200 μm and 20 μm for 1000× magnified right corner images. STD – standard diet group, HFHSD – high-fat and high-sucrose diet group, HFHSD+M – HFHSD treated with metformin, HFHSD+L – HFHSD treated with liraglutide.

**Extended data figure 5.1. High serum cholesterol was associated with HFHSD fed male groups.** Interaction plots of intervention and sex effects on the following parameters: **A)** serum cholesterol (mM) and triglycerides (mM); **B)** serum aspartate transaminase (AST) and alanine transaminase (ALT) (IU/L); **C)** liver mass to body mass ratio (%) (HFHSD+L females had the highest liver mass to body mass ratio potentially indicating liver edema); **D)** average size of fat droplets in skeletal muscle based on Oil red O staining (pixel^2^); **E)** lipid peroxidation (LPO) (thiobarbituric acid reactive substances, TBARS) of the skeletal muscle (nmol/mg of fresh tissue weight, FW). Two-way ANOVA and Games-Howell *post hoc* test for between-group comparisons, black symbol – experimental groups including both sexes, green symbol – male groups, red symbol – female groups, *compared to STD, ^†^compared to HFHSD, *^/†^p < 0.05, **^/††^p < 0.01, ***^/†††^p < 0.001. Abbreviations: NS – not significant, f – female, m – male, STD – standard diet group, HFHSD – high-fat high-sucrose diet group, HFHSD+M – HFHSD treated with metformin, HFHSD+L – HFHSD treated with liraglutide.

**Extended Data figure 5.2. Liver fat droplets visualized with Oil Red O.**

Abbreviations: CPL – central part of the liver, SPL – subcapsular part of the liver; STD – standard diet group, HFHSD – high-fat high-sucrose diet group, HFHSD+M – HFHSD treated with metformin, HFHSD+L – HFHSD treated with liraglutide.

**Extended Data figure 5.3. Metachromatic toluidine stain of liver for glycogen.** Abbreviations: CPL – central part of the liver, SPL – subcapsular part of the liver; STD – standard diet group, HFHSD – high-fat high-sucrose diet group, HFHSD+M – HFHSD treated with metformin, HFHSD+L – HFHSD treated with liraglutide

**Extended Data figure 5.4. Succinate dehydrogenase staining of skeletal muscle.** Intensely stained muscle fibers are type I, moderately stained muscle fibers are type IIA, weakly stained muscle fibers are type IIB. Abbreviations: STD – standard diet group, HFHSD – high-fat high-sucrose diet group, HFHSD+M – HFHSD treated with metformin, HFHSD+L – HFHSD treated with liraglutide.

**Extended Data figure 5.5. Oil red O staining of skeletal muscle.** Abbreviations: STD – standard diet group, HFHSD – high-fat high-sucrose diet group, HFHSD+M – HFHSD treated with metformin, HFHSD+L – HFHSD treated with liraglutide.

**Extended data figure 6.1.** Average TIC normalized mass spectra of Sprague-Dawley rats’ nuchal muscle recorded in range 300 – 700 Da. F – female; M – male; STD – standard diet group; HFHSD – high-fat high-sugar diet group; HFHSD + M – rats on HFHSD treated with metformin; HFHSD + L – rats on HFHSD treated with liraglutide;TIC – total-ion-current.

**Extended data figure 6.2.** Average TIC normalized mass spectra of Sprague-Dawley rats’ nuchal muscle recorded in range 700 – 1,000 Da. F – female; M – male; STD – standard diet group; HFHSD – high-fat high-sugar diet group; HFHSD + M – rats on HFHSD treated with metformin; HFHSD + L – rats on HFHSD treated with liraglutide;TIC – total-ion-current.

**Extended data figure 6.3.** Mass spectrometry images of selected metabolites Xanthurenic acid 8-O-sulfate, inosine monophosphate (IMP), and phosphatidic acids (PA) C38:5 laid over the corresponding non-stained optical microscopy images of male and female Sprague-Dawley rats' nuchal skeletal muscles. F – female; M – male; STD – standard diet group; HFHSD – high-fat high-sugar diet group; HFHSD + M – rats on HFHSD treated with metformin; HFHSD + L – rats on HFHSD treated with liraglutide.

**Extended data figure 6.4.** Heatmap constructed using Euclid distance and Ward's method applied on the scaled significant m/z signals coming from the nuchla muscle. F – female; M – male; STD – standard diet group; HFHSD – high-fat high-sugar diet group; HFHSD + M – rats on HFHSD treated with metformin; HFHSD + L – rats on HFHSD treated with liraglutide.
