# Supplementary figures and images for: Elderly rats fed with a high-fat high-sucrose diet developed sex-dependent metabolic syndrome regardless of long-term metformin and liraglutide treatment
Source: Front Endocrinol (Lausanne). 2023 Oct 20;14:1181064. doi: 10.3389/fendo.2023.1181064 (PMC10623428; doi:10.3389/fendo.2023.1181064)

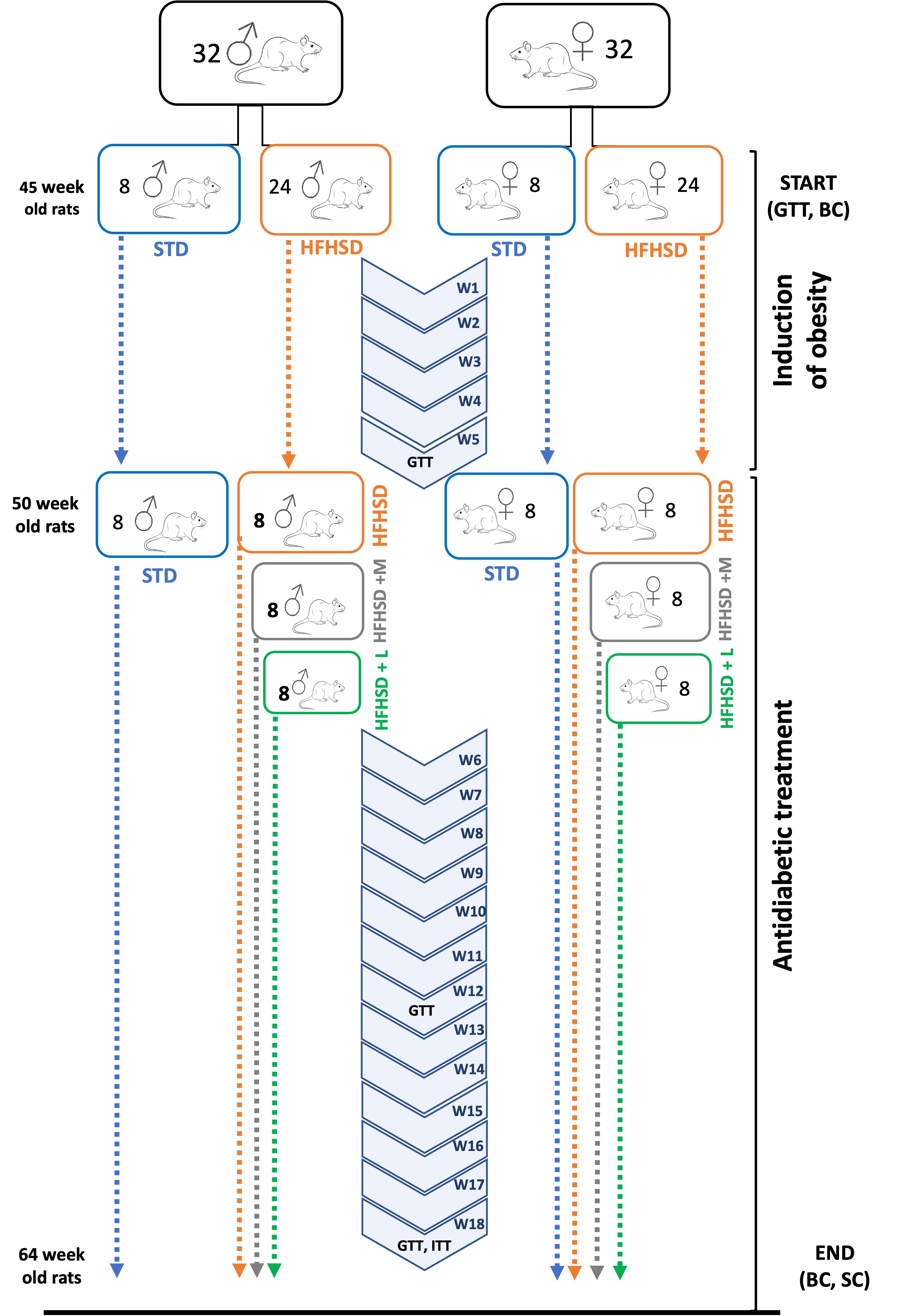

Supplement: Supplementary file 1 [file DataSheet_1.zip › Extended Data/Extended Data Fig. 1.JPEG]

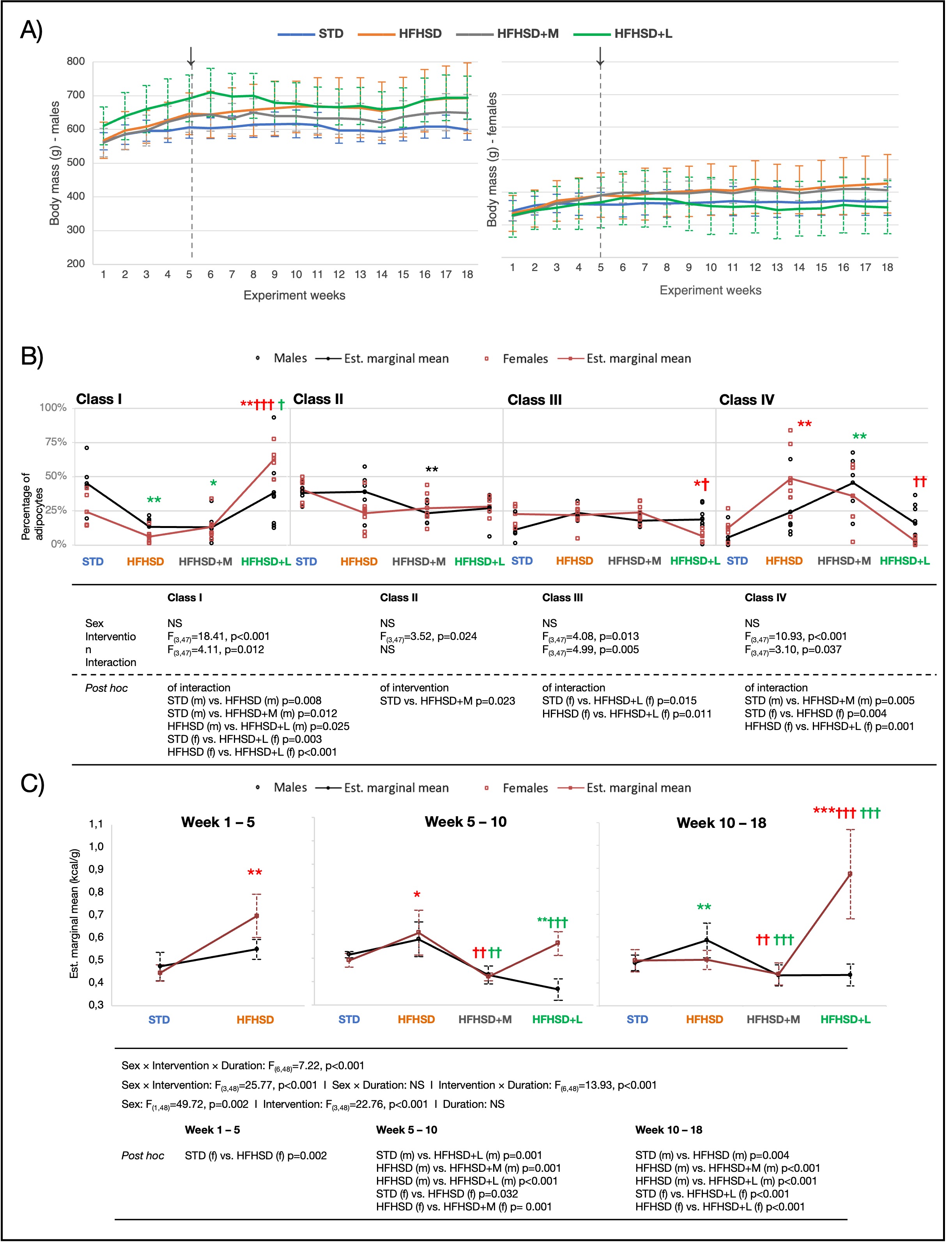

Supplement: Supplementary file 1 [file DataSheet_1.zip › Extended Data/Extended Data Fig. 2.JPEG]

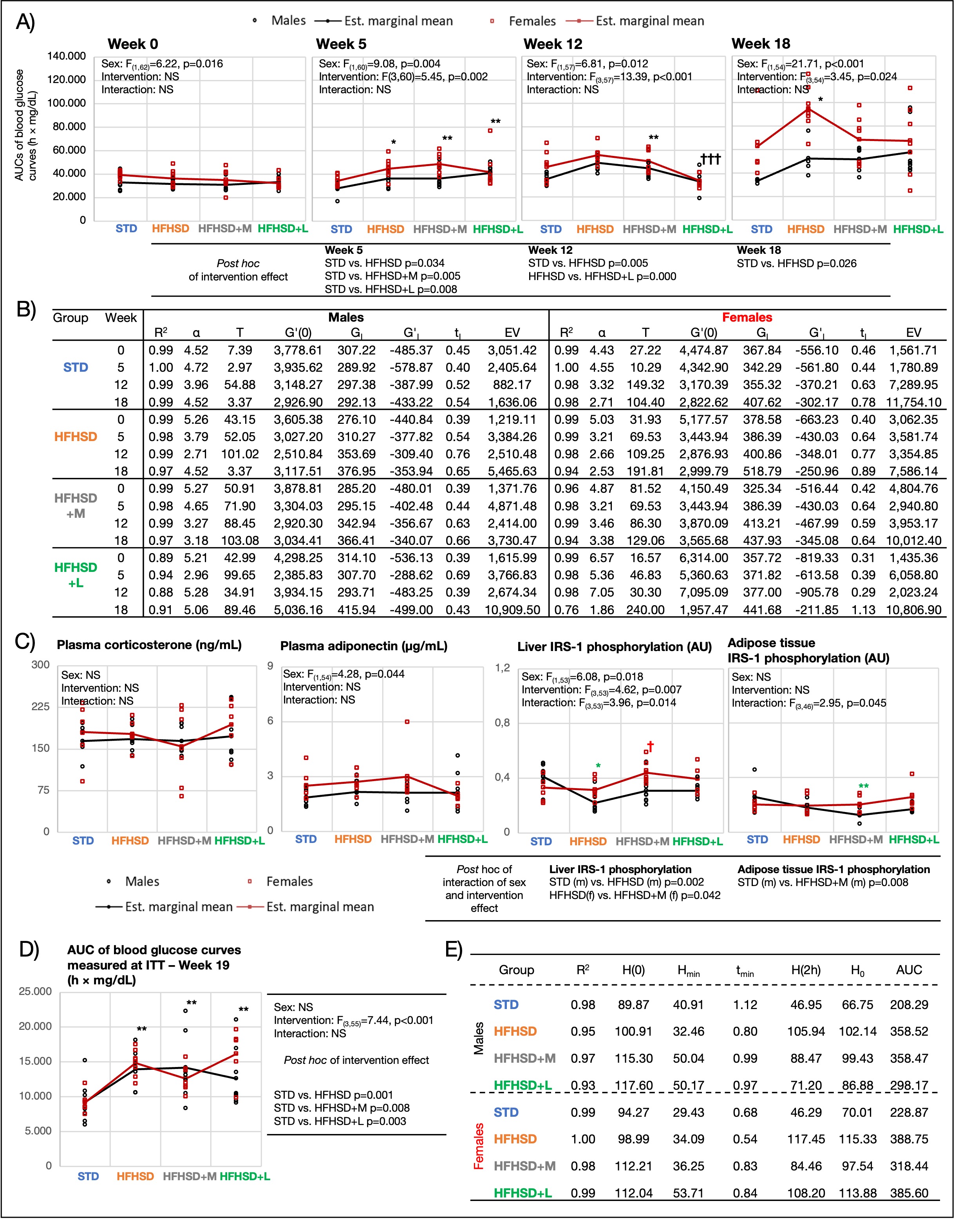

Supplement: Supplementary file 1 [file DataSheet_1.zip › Extended Data/Extended Data Fig. 3.JPEG]
